# Exploring the Parameters Controlling Product Selectivity in Electrochemical CO_2_ Reduction in Competition with Hydrogen Evolution Employing Manganese Bipyridine Complexes

**DOI:** 10.1021/acscatal.2c05951

**Published:** 2023-02-16

**Authors:** Wanwan Hong, Mahika Luthra, Joakim B. Jakobsen, Monica R. Madsen, Abril C. Castro, Hans Christian D. Hammershøj, Steen U. Pedersen, David Balcells, Troels Skrydstrup, Kim Daasbjerg, Ainara Nova

**Affiliations:** † Carbon Dioxide Activation Center (CADIAC), Interdisciplinary Nanoscience Center, Department of Chemistry, Aarhus University, Gustav Wieds Vej 14, 8000 Aarhus C, Denmark; ‡ Hylleraas Centre for Quantum Molecular Sciences, Department of Chemistry, University of Oslo, 0315 Oslo, Norway; § Interdisciplinary Nanoscience Center, Department of Chemistry, Aarhus University, Langelandsgade 140, 8000 Aarhus C, Denmark; ∥ Novo Nordisk Foundation (NNF) CO_2_ Research Center, Interdisciplinary Nanoscience Center, Department of Chemistry, Aarhus University, Gustav Wieds Vej 10C, 8000 Aarhus C, Denmark; ⊥ Centre for Materials Science and Nanotechnology, Department of Chemistry, University of Oslo, 0315 Oslo, Norway; †† Carbon Dioxide Activation Center (CADIAC), Novo Nordisk Foundation (NNF) CO_2_ Research Center, Interdisciplinary Nanoscience Center, Department of Chemistry, Aarhus University, Gustav Wieds Vej 14, 8000 Aarhus C, Denmark

**Keywords:** carbon dioxide reduction, hydrogen evolution reaction, manganese bipyridine complexes, density functional theory calculations, mechanisms, electrocatalysis

## Abstract

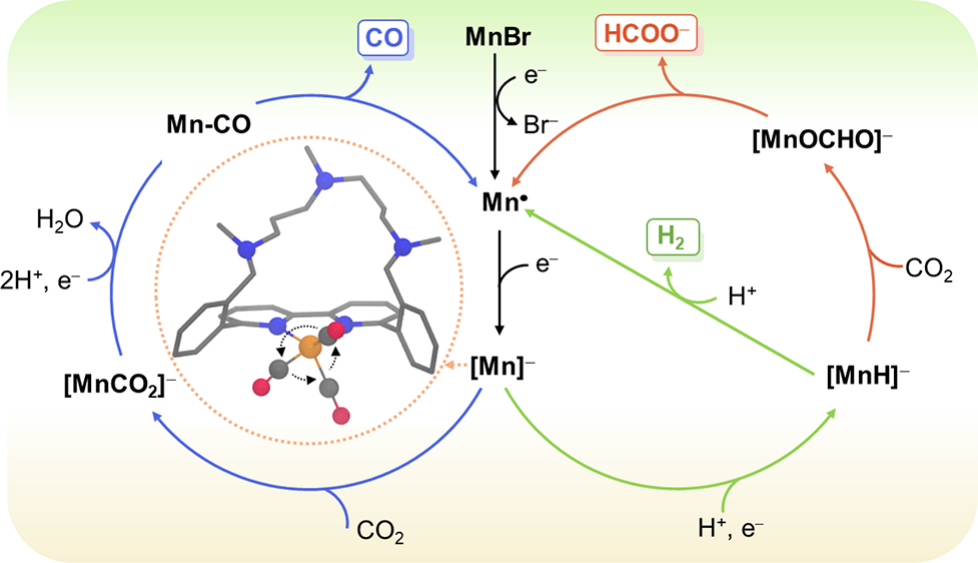

Selective reduction of CO_2_ is an efficient solution for producing nonfossil-based chemical feedstocks and simultaneously alleviating the increasing atmospheric concentration of this greenhouse gas. With this aim, molecular electrocatalysts are being extensively studied, although selectivity remains an issue. In this work, a combined experimental–computational study explores how the molecular structure of Mn-based complexes determines the dominant product in the reduction of CO_2_ to HCOOH, CO, and H_2_. In contrast to previous Mn(bpy-R)(CO)_3_Br catalysts containing alkyl amines in the vicinity of the Br ligand, here, we report that bpy-based macrocycles locking these amines at the side opposite to the Br ligand change the product selectivity from HCOOH to H_2_. *Ab initio* molecular dynamics simulations of the active species showed that free rotation of the Mn(CO)_3_ moiety allows for the approach of the protonated amine to the reactive center yielding a Mn-hydride intermediate, which is the key in the formation of H_2_ and HCOOH. Additional studies with DFT methods showed that the macrocyclic moiety hinders the insertion of CO_2_ to the metal hydride favoring the formation of H_2_ over HCOOH. Further, our results suggest that the minor CO product observed experimentally is formed when CO_2_ adds to Mn on the side opposite to the amine ligand before protonation. These results show how product selectivity can be modulated by ligand design in Mn-based catalysts, providing atomistic details that can be leveraged in the development of a fully selective system.

## Introduction

Catalysis is crucial for modern society and life in general. Catalytic processes occur in the mitochondria, where glucose is converted into energy, in refineries, where crude oil is converted into fuels, in the polymer industry, facilitating the production of plastics for computers, houses, and clothes, and in the pharmaceutical industry, where vital medicine is produced. In fact, 85% of all products manufactured have been produced with the assistance of a catalyst, and in 90% of all chemical processes, at least one catalyst is employed.^1^ Despite the huge number of existing catalysts, it is still of high priority to develop new and more efficient ones that with low cost, low energy consumption, and low environmental impact selectively convert building blocks into target compounds.^2^ Electrocatalysts show great promise in this respect, as they can be powered by renewable energy sources while operating at ambient temperature and pressure.^3^ For instance, electrocatalysts are widely used in the electrolysis of water, where water is decomposed into oxygen and hydrogen gas, of which the latter is a promising carbon-neutral fuel that does not emit harmful exhaust gases.^4,5^ Furthermore, research regarding electrochemical CO_2_ reduction is advancing with the prospect of decreasing CO_2_ pollution while converting the greenhouse gas into value-added fuels and chemicals.^6−8^

**Figure 1:**
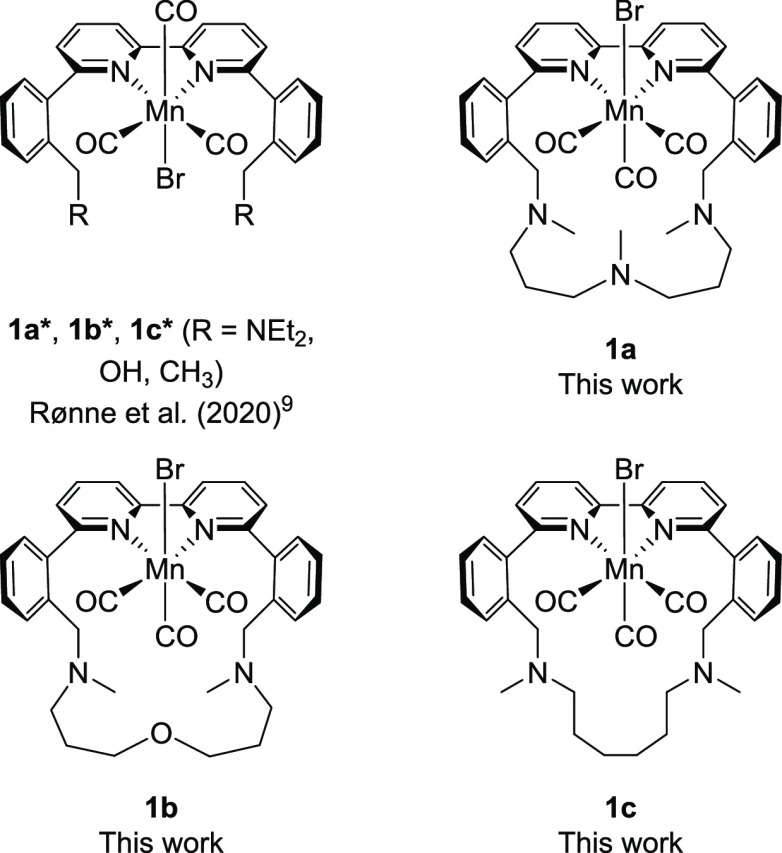
Chemical structures of reference complexes (1a*, 1b*, and 1c*) reported in our previous work^9^ and complexes **1a**, **1b**, and **1c** reported herein.

**Scheme 1:**
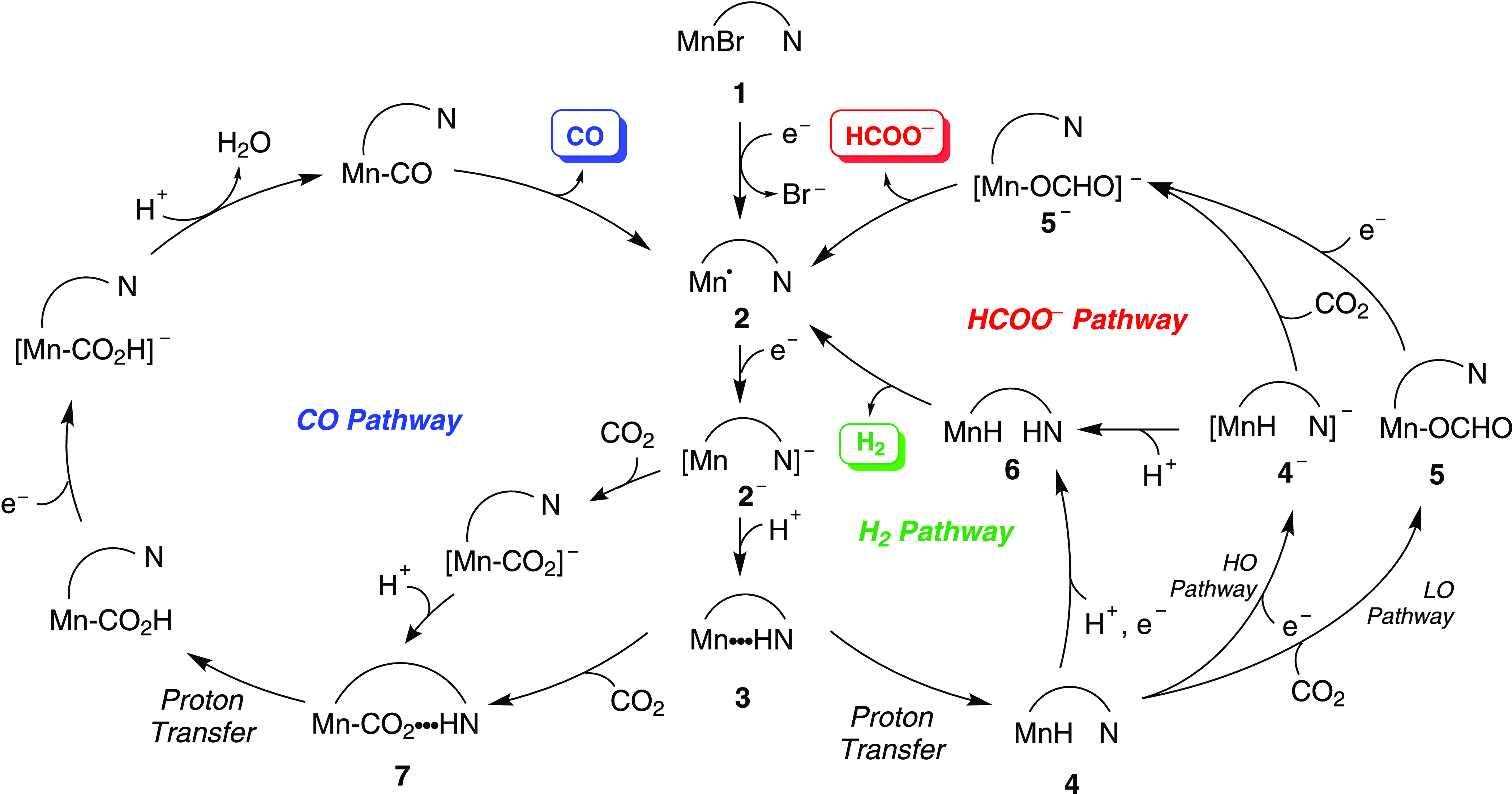
Pathways for CO (blue), H_2_ (green), and HCOO^–^ (red) Formation with Mn Bipyridine Complexes Bearing Amines in the Secondary Coordination Sphere Previously Reported and Proposed in This Work The arc connects the amine-bearing pendants in the secondary coordination sphere, while only one of the sides is included for simplicity. LO and HO stand for low overpotential and high overpotential, respectively.^9−11^

A recurring challenge within catalysis is to obtain a high selectivity toward a single product. Within the family of metal-based molecular catalysts, a lot of research has focused on secondary coordination sphere effects and how different functional groups influence the selectivity of the complexes.^12−16^ Recently, we showed how the selectivity of CO_2_ reduction by Mn bipyridine (bpy) catalysts (1a*–c*, Figure 1) changes from CO, when hydroxyl (1b*) or alkyl groups (1c*) are close to the metal center, to HCOOH, when amines (1a*) are placed in the vicinity of the metal.^9^ As presented in Scheme 1, the amines function as proton shuttles, transferring protons from the ligand to the metal center, thereby facilitating the formation of a Mn hydride, **4**, which is the key intermediate in the HCOO^–^/HCOOH and H_2_ pathways. Additionally, the presence of the amine functionalities is also known to stabilize the carboxylate intermediate, **7**, by forming an intramolecular hydrogen bond, which then favors CO formation (blue pathway, Scheme 1).^17−19^ Since amines accelerate all three pathways, the selectivity originates from other contributing factors, such as the nature of the metal,^20^ geometrical structure,^21^ hydricity,^22^ and p*K*_a_.^23^ In the case of Mn complexes, CO has been the dominant product for the nonamine-containing complexes (1b* and 1c*).

Since the abovementioned pathways always compete with each other, especially when there is a bifurcation between the H_2_ and HCOO^–^ pathways from a common Mn hydride intermediate, we explored how the structure affects the selectivity of the catalysts within the Mn bpy family. The structure has already been shown to affect the catalytic activity, as an unsubstituted Mn bpy complex forms less reactive dimers upon reduction,^24^ which is avoided by introducing bulky substituents on the ligands.^25^ However, the fundamental understanding of how the molecular structure and product distribution are linked is still missing. To investigate this correlation more carefully, three different Mn tricarbonyl complexes with macrocyclic bipyridine ligands were synthesized (**1a**–**c**, Figure 1). Using our previously reported complex 1a*, bearing tertiary amines in the secondary coordination sphere, as a reference catalyst, here, we aim to explore the effect of locking all the amine groups in a relatively enclosed structure by introducing a macrocyclic linker. Furthermore, we examine if the central heteroatom of the linker (N or O, for complexes **1a** and **1b**, respectively) affects the product distribution, while the size of the macrocyclic ring is evaluated with the one-atom shorter complex **1c**.

With these complexes in hand, the different possible pathways proposed in Scheme 1 are scrutinized to get a clearer mechanistic understanding of the factors affecting the product distribution. Specifically, the order of competing protonation and CO_2_ insertion reactions is considered, and the energy barriers of these steps are evaluated by density functional theory (DFT) calculations. Furthermore, information about the flexibility of the molecular structures is gained through *ab initio* molecular dynamics (AIMD). This fundamental study builds on an electrochemical assessment, taking complex **1a** as a starting point, in which the key intermediates formed during reduction are identified using infrared spectroelectrochemistry (IR-SEC). Finally, based on the mechanistic insight obtained from our combined experimental and computational studies, we propose a general guide for rational ligand design.

## Results and Discussion

### Synthesis and Characterization of Complexes

**Scheme 2:**
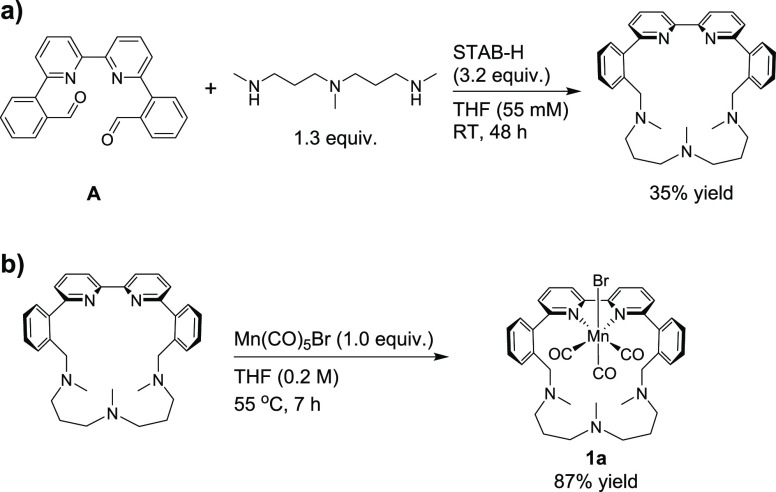
Synthetic Protocol Employed Toward Complex **1a**

The three macrocyclic complexes (**1a**–**c**) were synthesized as exemplified for **1a** in Scheme 2. The synthesis was envisioned to proceed through a double reductive amination from dialdehyde **A** using a small excess of the corresponding di- or triamines. To minimize the formation of larger ring sizes or polymers, the reaction was performed using a low concentration of dialdehyde **A** (55 mM) in tetrahydrofuran (THF), and a syringe pump was employed to slowly add the amines over five hours (Scheme 2a). After stirring for 48 h, full conversion of dialdehyde **A** was obtained in all three cases, and the macrocyclic ligands could be obtained in 13–48% yield after purification. Complexes **1a**–**c** were obtained by metalation of the respective ligands using Mn(CO)_5_Br in THF at 55 °C yielding the complexes as yellow solids in good yields (Scheme 2b). The complexes were analyzed by ^1^H NMR, ^13^C NMR, ATR-IR, and high-resolution mass spectrometry (HR-MS). Furthermore, the ^1^H NMR and ^13^C NMR chemical shifts of complex **1a** were calculated and found to be in good agreement with the experimental values (Table S1).

**Figure 2:**
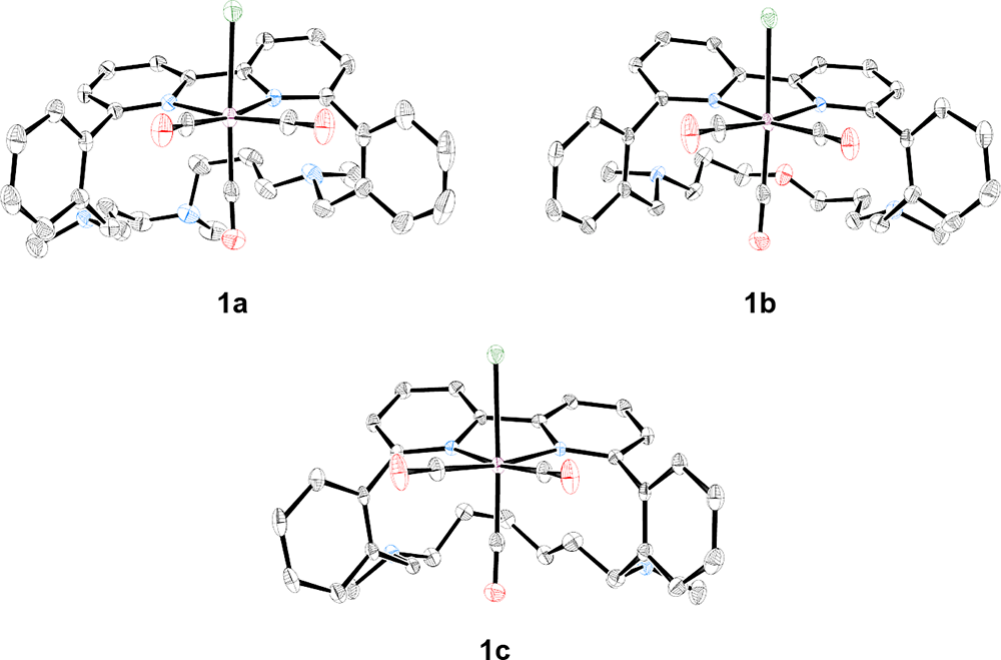
Molecular structures of **1a**, **1b**, and **1c** obtained from single-crystal X-ray diffraction studies. Mn = maroon, Br = green, C = gray, N = blue, and O = red; thermal ellipsoids at 50% probability. Hydrogen atoms are omitted for clarity.

Figure 2 shows the single-crystal X-ray diffraction structure of **1a**, and its crystallographic data are listed in the Supporting Information (Section 2). In the solid state, the Mn center adopts a facial octahedral geometry, having the modified bpy ligand and two carbonyl ligands in the equatorial plane, while the bromide is placed in the axial position together with the third carbonyl group pointing in opposite directions. Interestingly, the Mn–Br bond is in the direction opposite to the aliphatic amine ligand. The bpy plane is slightly distorted (N–C–C–N dihedral angle, φ = 11.6°), while the adjacent phenyl rings are rotated out of the plane 60–80°. The linker between the two phenyl rings is situated below the plane of the bpy ring system. This is an interesting observation as it implies that no amines are in close vicinity to the Mn center, which we previously have shown is important for the product selectivity.^9^ The single-crystal X-ray diffraction structures of **1b** and **1c** are shown in Figure 2, and crystallographic data are listed in the Supporting Information (Section 2). The coordination sphere of Mn is facial octahedral for both complexes, and the general observations mentioned above for **1a** also hold true for **1b** and **1c**. Only one isomer with the Br in the opposite direction to the ligand (*exo*) is observed for **1a–c** in solution, which is also supported by DFT calculations (TPSSh-D3/def2SVP//TPSSh-D3/def2TZVP, def2TZVPD, see Section 3 in the Supporting Information for details). It was found that the *exo* isomers of **1a**, **1b**, and **1c** were 8.4, 8.2, and 7.4 kcal mol^–1^, respectively, more stable than the corresponding *endo* isomers, characterized by Br and amine pointing in the same direction (Scheme S1).

### AIMD Simulations

**Figure 3:**
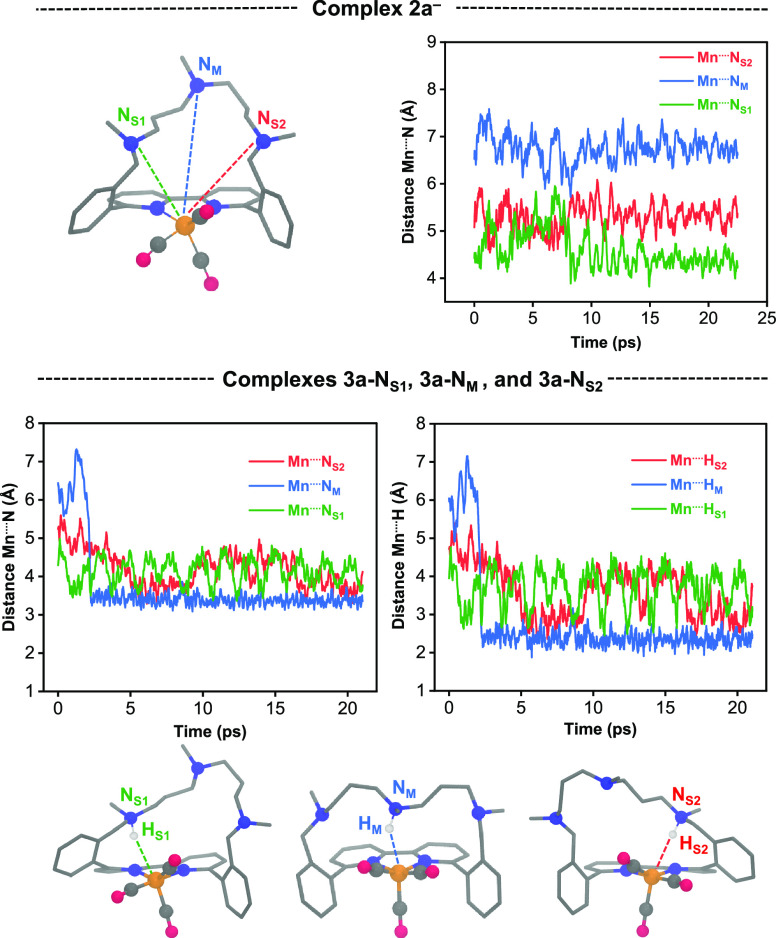
Time evolution of (top) the Mn···N distances (Å) for **2a^–^** and (bottom) the Mn···N(H) and Mn···H(N) distances for the three protonated isomers of the corresponding **3a-N_X_** (X = M, S1, or S2) complexes. The three amine N atoms of the chelating ligand are labeled as N_M_ (middle one) and N_S1_ and N_S2_ (the two on the sides).

Formation of the hydride intermediate in the H_2_ pathway (Scheme 1) requires the proximity of the chelating-ligand amines to the metal center, which seems to be excluded by the crystal structure of **1a** (Figure 2). However, this reaction is preceded by the two-electron reduction of **1a** and the concomitant elimination of bromide, yielding an anion species **2a^–^**, and the protonation of the amine moiety of the latter to form **3a** (Scheme 1 and Figure 3). Hence, we studied the fluxional behavior of the ligand in these two species by means of DFT AIMD (PBE-D3/DZVP) as implemented in the CP2K program^26−28^ followed by static DFT calculations.

Figure 3 shows the time evolution (within a production trajectory of 25 ps) of the Mn···H distances for complex **2a^–^**, involving the three amine N atoms of the ligand: the middle one (N_M_) and the two on the sides (N_S1_ and N_S2_). The side N atoms were initially considered different because the ligand is asymmetric in the crystal structure. However, their dynamic behavior should be equivalent at longer trajectories.

The AIMD simulation of **2a^–^** reflects the dynamic structure of the chelating ligand, in which the variation of the Mn···N distances has wide amplitudes [1.86 (N_M_), 2.13 (N_S1_), and 1.54 Å (N_S2_)] and large average values [6.74 (N_M_), 4.61 (N_S1_), and 5.29 Å (N_S2_)]. These distances are indeed long and similar to those observed in the crystal structure of **1a** [7.06 (N_M_), 4.73 (N_S2_), and 5.10 Å (N_S2_)]. Further, the N_M_ atom, which corresponds to the most flexible amine, is located at the position furthest from the metal center. Interestingly, this behavior is reversed when **2a^–^** is protonated to **3a** (Figure 3); i.e., the shortest average Mn···N(H) distance is observed when the proton binds to the middle N_M_ atom, yielding an average value of 3.39 Å (measured after the sudden change at ∼2 ps). For all three protonated isomers, **3a-N_M_**, **3a-N_S1_**, and **3a-N_S2_**, the time evolution of the Mn···N(H) distances is strongly correlated to that of the corresponding Mn···H(N).

Therefore, the shortest average Mn···H(N) distance was also observed for N_M_, with a small value of 2.38 Å, suggesting the presence of a hydrogen bond between Mn and the H–N moiety. This is consistent with the dramatic shortening of the average Mn···N_M_ distance by 3.35 Å upon protonating **2a^–^**, and the narrowing of its amplitude to 0.78 Å. In contrast, the Mn···N_S_(H) and Mn···H(N_S_) distances in **3a-N_S1/2_** fluctuate, as indicated by their large amplitudes in the range of 2.31 and 3.09 Å. This indicates that even though the formation of a hydrogen bond is feasible (shortest Mn···H(N_S1_) distance = 2.30 Å), this interaction is weaker than that in **3a-N_M_** (shortest Mn···H(N_M_) distance = 1.87 Å) (Table S2).

The fluxional behavior of **2a^–^** and **3a-N_X_** (X = M, S1, and S2) can be ascribed to the rotation of the Mn(CO)_3_ core (Figure S1). The AIMD simulation of **2a^–^** shows that this rotation causes an oscillation of the metal axial vacancy in between two positions: one pointing to the triamine bridge (*endo*) and the other to the opposite direction (*exo*). In the *endo* form, the interaction between the N atoms and the vacancy is repulsive because both moieties have lone pairs (Figure S2). Conversely, in the **3a-N_M_** complex, this interaction becomes attractive, which yields a Mn···H–N hydrogen bond. The *exo* to *endo* flip can be observed during the initial 5 ps of the AIMD of **3a-N_M_** (Figure 3), in which both the Mn···N_M_(H) and Mn···H(N_M_) distances undergo a sudden shortening of ∼3 Å.

The calculations thus show that, despite the long distance observed in the crystal structure of **1a**, the middle amine can approach the metal center in the crucial intermediate **3a**, facilitating the formation of the hydride complex **4a**. The less flexible side positions could also allow the formation of a hydride, but it seems less preferred.

### Electrochemical and Spectroscopic Evaluation

**Figure 4:**
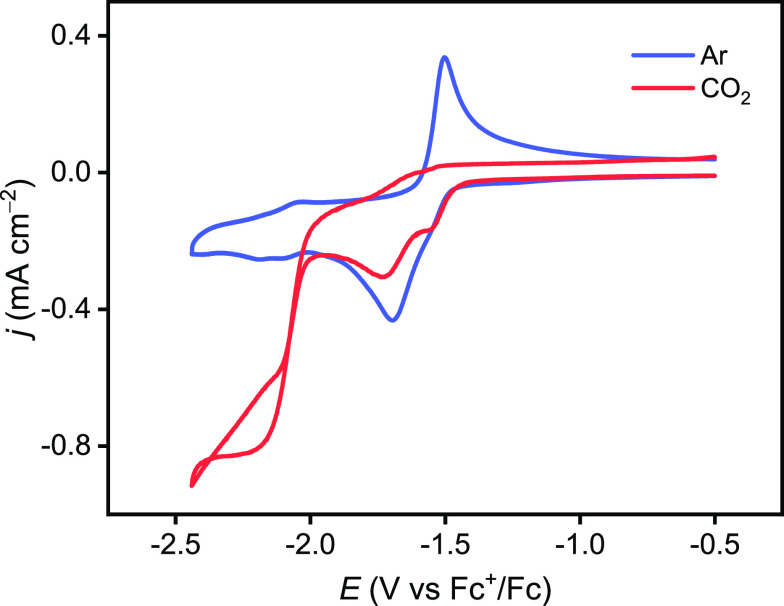
Cyclic voltammograms recorded on 1.5 mM **1a** at a GC electrode (diameter = 1 mm) using ν = 0.1 V s^–1^ in Ar- or CO_2_-saturated 0.1 M Bu_4_NBF_4_/MeCN.

Electrochemical experiments were conducted to determine how the macrocyclic bipyridine ligands influence the electrocatalytic properties of the manganese complexes. Cyclic voltammograms of complexes **1a**–**c** are recorded in 0.1 M Bu_4_NBF_4_/MeCN solutions under first Ar- and then CO_2_-saturated conditions (Figure 4 and Figures S3 and S4). Analogously to our findings for complex 1a*–c*,^9^ all three complexes present one reduction wave at around −1.69 V vs Fc^+^/Fc under Ar atmosphere. According to the previous mechanistic interpretation,^9,25^ this reduction wave is the result of a consecutive electron transfer–chemical reaction–electron transfer (ECE) mechanism where the first one-electron reduction of **Mn(bpy-R)(CO)_3_Br** (complex **1** in Scheme 1) is followed by the dissociation of Br^–^, generating a neutral intermediate **2**.^11^ The second electron is transferred from the electrode to **2** instantly since **2** is formed very close to the electrode surface due to the rapid dissociation of Br^–^ and is easier to reduce than **1** itself. Thus, the overall two-electron reduction gives rise to the anionic state **2^–^**,^29^ as shown in Scheme 1. The process is diffusion-controlled with unaltered electron stoichiometry according to the Randles–Ševčík equation (Figure S5).^25^

**Scheme 3:**
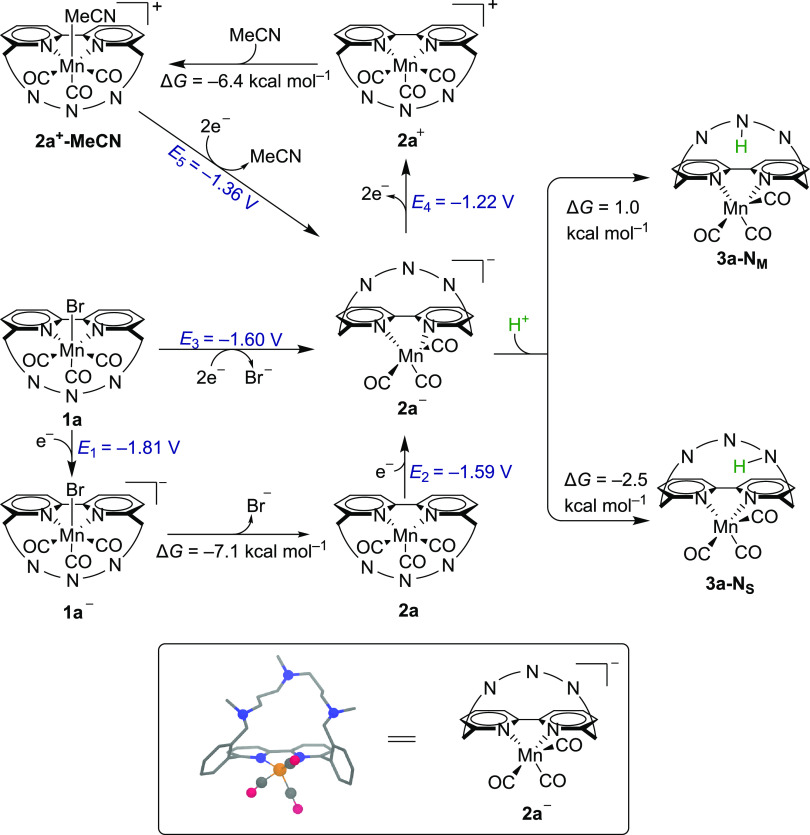
Computational Study on the Reduction Path of **1a** Comprising Two Kinds of Amine Moieties in the Ligand All potential values are calculated relative to Fc^+^/Fc (see the Supporting Information for details). Protonation steps assume that the proton source is CF_3_CH_2_OH/CO_2_.

The above interpretation was also confirmed by DFT calculations (see the Supporting Information for further details) taking **1a** as an example (Scheme 3). The first reduction from **1a** to **1a^–^** has been calculated to take place at *E*_1_ = −1.81 V vs Fc^+^/Fc, after which Br^–^ is easily expelled to form **2a** with Δ*G* = −7.1 kcal mol^–1^. The analysis of the spin density in **1a^–^** (Table S3) shows that the electron is localized at the bpy ligand as found in similar complexes.^30−32^ The second reduction generating the catalytically active intermediate **2a^–^** has been computed to occur at *E*_2_ = −1.59 V vs Fc^+^/Fc. Taken together, these three processes yield a two-electron reduction taking place at *E*_3_ = −1.60 V vs Fc^+^/Fc. This overall potential *E*_3_ is positively shifted compared to *E*_1_ due to the follow-up dissociation of Br^–^, which is in good accordance with the experimental potential of −1.69 V vs Fc^+^/Fc.

**Scheme 4:**
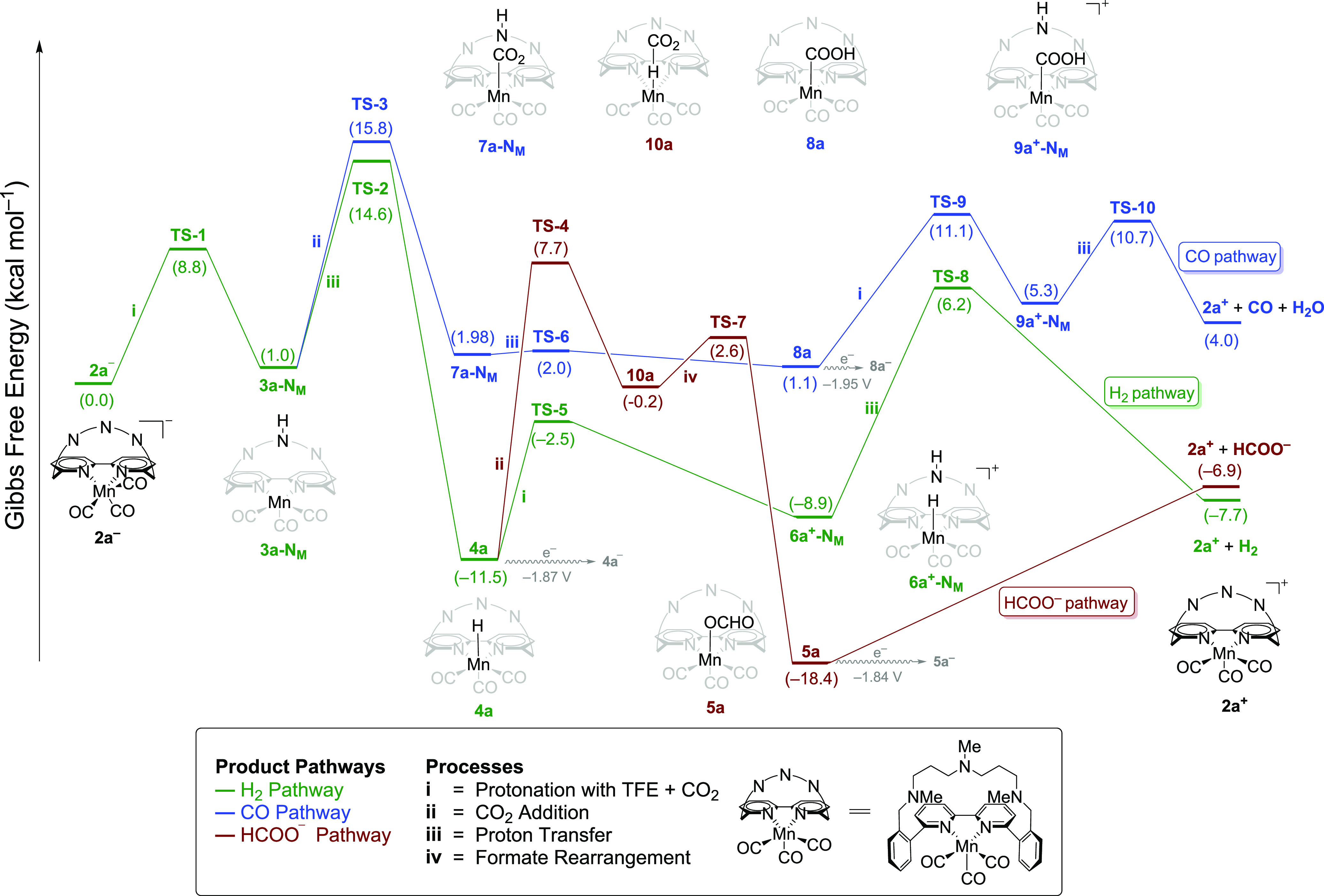
DFT-Calculated Energy Profile for the Reduction of CO_2_ to Three Different Products: H_2_ (Green), HCOO^–^ (Red), and CO (Blue) Using the *endo* Profile and the Middle (N_M_) Amine Moiety as a Proton Shuttle^,^^,^^,^ The *endo* profile refers to the pentacoordinate complex with the ligand in the same direction as the metal vacant site. All energies are considered at a redox potential of −1.60 V vs Fc^+^/Fc. Wiggly arrows show the calculated reduction potentials for **4a**, **5a**, and **8a**. Energy profiles after reduction are displayed in Scheme 5 (at −1.87 V vs Fc^+^/Fc) and Scheme S6 (at −1.95 V vs Fc^+^/Fc). Numbers in the energy profile indicate relative Gibbs free energy in kcal mol^–1^. Some intermediates have been omitted for clarity. The complete energy profiles are presented in Scheme S9.

**Scheme 5:**
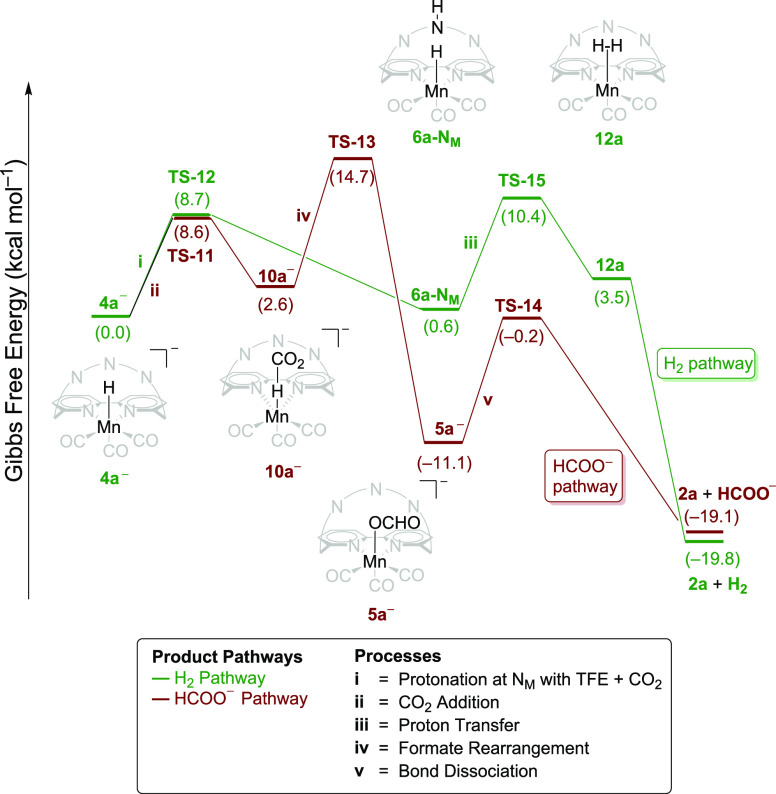
DFT-Calculated Energy Profile Starting from **4a^–^** to Form H_2_ (Green) and HCOO^–^ (Red) Using the *endo* Profile and the Middle (N_M_) Amine Moiety as a Proton Shuttle^,^ All energies are relative to a redox potential of −1.87 V vs Fc^+^/Fc. Numbers in the energy profile indicate relative Gibbs free energy in kcal mol^–1^.

Infrared spectroelectrochemistry analysis of **1a**, in the absence of CO_2_ and a proton source, further uncovers the formation of anionic species **2a^–^** since the CO stretches shift from 2021, 1936, and 1906 cm^–1^ (**1a**) to 1909 and 1807 cm^–1^ during the voltammetric sweeping (Figure S6), in alignment with previous reports.^25,33,34^ In addition, the stretch at 1982 cm^–1^ and the bump at 1884 cm^–1^ stem from manganese hydride **4a** (*vide infra* in Schemes 4 and 5).^9,35^ Its appearance is a consequence of the amine-bearing ligand, which shuttles protons from residual water in the electrolyte to the metal center to generate **4a**. Hence, we assign the faint peak at around −2.10 V vs Fc^+^/Fc in Figure 4 under Ar to the reduction of **4a**.^36,37^ A similar mechanism was outlined by us for complex 1a*.^9^

Now addressing the CO_2_-saturated electrolyte ([CO_2_] ≈ 0.28 M),^38^ significant changes occur to the cyclic voltammetric response. Notably, the oxidation waves of the anionic species disappear (see Figure 4 and Figures S3 and S4). A prepeak appears at −1.55 V vs Fc^+^/Fc for **1a** and **1c**, which is associated with the reduction of the solvent-coordinated cation originating from partial solvolysis of **1a** and **1c**.^24^ This behavior was also reported for analogous Mn complexes.^35,37^ In addition, DFT calculations are in agreement with this assignment. We found that the solvent-coordinated complex, **2a^+^-MeCN**, is also generated through a two-electron oxidation process of **2a^–^** at *E*_4_ = −1.22 V vs Fc^+^/Fc followed by the exergonic coordination of MeCN with Δ*G* = − 6.4 kcal mol^–1^, which is reduced at a potential of *E*_5_ = −1.36 V vs Fc^+^/Fc (Scheme 3). At the same time, a dramatic current enhancement is detected at −2.10 V vs Fc^+^/Fc. This is in accordance with a high-overpotential pathway, in which the hydride species **4** is reduced and subsequently enters the catalytic cycle.^9^ The trace crossing in Figure 4 and Figures S3 and S4 indicates an acceleration of the catalytic behavior on the reverse scan (see Section 5 in the Supporting Information for a detailed explanation).^39^ The specific catalytic effect of residual water (∼0.034 M) in combination with CO_2_ (Figure 4) is discussed thoroughly in Section 6 of the Supporting Information. Upon sequential addition of either 2,2,2-trifluoroethanol (TFE, p*K*_a_ = 35.4 in MeCN,^40^Figure S7) or 2-propanol (*i*PrOH, p*K*_a_ ≈ 42 in MeCN,^37^Figure S8), the catalytic current continues increasing, until it levels off or even drops once very high concentrations of TFE (2.0 M) or *i*PrOH (1.0 M) are employed. Thus, under these conditions, the CO_2_ reduction reaction is independent of the proton concentration but limited by the regeneration of the catalyst, as often observed for catalytic reactions.^9,25^

Upon introducing CO_2_ to the IR-SEC experiments of **1a**, a mixture of **2a^–^** (1909 and 1807 cm^–1^) and **4a** (1982 and 1884 cm^–1^) is observed during reduction (Figure S9), where the significant increase in the amount of **4a** formed can be attributed to the acidification of the solution induced by CO_2_, i.e., CO_2_ increases the proton-donating ability of residual water. Introducing a proton source (TFE or *i*PrOH) makes the signals assigned to **2a^–^** vanish. In contrast, the signals from **4a** are strong, highlighting how proton sources facilitate the hydride generation (Figures S10 and S11). As depicted in Scheme 1, **4** is a vital intermediate in the catalytic cycle, where it either is further protonated to release H_2_ or combines with CO_2_ to generate a formato complex **5**.^22,41,42^ A first assessment of the possible CO_2_ reduction products can be gained from the IR-SEC spectra recorded in the region of 1750–1500 cm^–1^ with either TFE or *i*PrOH as a proton source. The bands at 1691 (Figure S12) and 1660 cm^–1^ (Figure S13) are assigned to the C=O stretches of trifluoroethyl- and isopropyl carbonates, respectively, while the signal at 1609 cm^–1^ in both cases is attributed to the formation of HCOO^–^, which is produced when CO_2_ inserts into the Mn–H bond.^9,37,43^ In addition, the signal at 1609 cm^–1^ shows a weaker intensity when TFE is present compared with that in the case of *i*PrOH, which could be related to the diminished generation of HCOO^–^.

**Figure 5:**
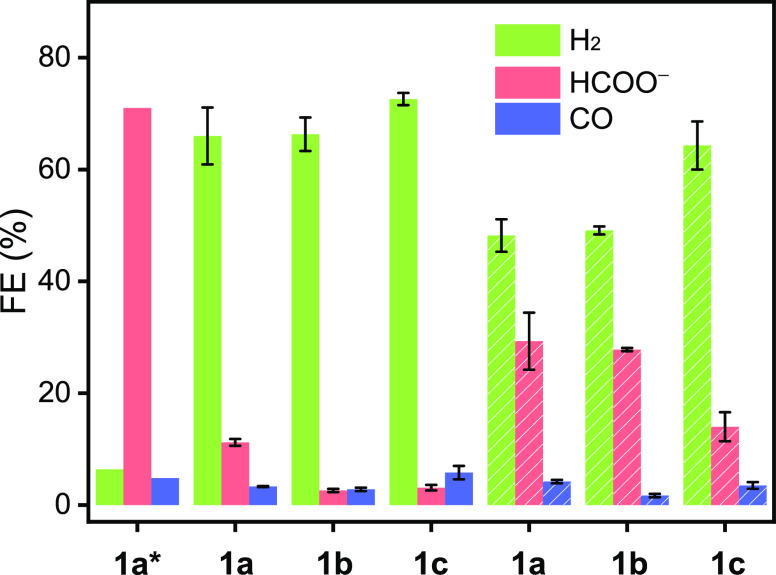
Product distributions obtained after 1 h of electrolysis (at −2.25 V vs Fc^+^/Fc) of 1.5 mM 1a*(9) or **1a**–**c** in CO_2_-saturated 0.2 M Bu_4_NBF_4_/MeCN containing either 2.0 M TFE (fully colored) or 1.0 M *i*PrOH (shaded colored) as a proton source.

To gain further insights, we turned to controlled potential electrolysis (CPE) to analyze the product distribution on a longer time scale than cyclic voltammetry and IR-SEC experiments. CPE was performed for 1 h at −2.25 V vs Fc^+^/Fc (∼150 mV more negative than the reduction of the hydride species) for **1a**–**c** using either 2.0 M TFE or 1.0 M *i*PrOH as a proton source. All experiments were carried out in a two-chamber H-cell following the same procedure as reported in our previous work.^9,20^ While the reference complex, 1a*, produces HCOOH (or HCOO^–^) as the dominant product,^9^ complexes **1a**–**c** show high selectivity for H_2_ (Figure 5 and Table S5). In general, only small amounts of HCOO^–^ and CO are produced for all three complexes when TFE is employed as a proton donor; the amounts of HCOO^–^ increase slightly when using the weaker proton donor, *i*PrOH. This observation can be explained by the competing pathways described in Scheme 1, where **4** either reacts with CO_2_ to form HCOO^–^ or H^+^ to produce H_2_, of which the latter option is favored when stronger proton donors are used.^14,35,37,44^ This result also suggests that the highest energy barriers for the formation of these two products are very similar (*vide infra*).

Specifically, complexes **1a** and **1b** with the same linker length but different central hetero atoms (N and O, respectively) show both a high Faradaic efficiency for H_2_ (FE_H2_) of ∼66% with 2.0 M TFE and ∼48% with 1.0 M *i*PrOH. The FE_HCOO-_ also displays a decent value of ∼29% for **1a** and **1b** with 1.0 M *i*PrOH. These results indicate that, even in the absence of the middle N, the side N in **1b** can act as proton shuttle favoring the formation of the intermediate **4b**. This is also consistent with the AIMD simulations using **3a-N_S1/2_** (Figure 3). Shortening the macrocyclic ring by leaving the central heteroatom out (**1c**) leads to an enhancement of FE_H2_, which reaches a maximum of 73% with 2.0 M TFE. At the same time, FE_HCOO–_ is suppressed regardless of the proton source. CO is also produced in negligible quantities (FE_CO_ = 2–6%) in all cases.

In a control experiment using no catalyst, we noted that only ∼2.9 μmol of H_2_ was produced in the presence of 2.0 M TFE during the 1 h CPE at -2.25 V vs Fc^+^/Fc. This is significantly less than the yield of H_2_ generated in the presence of **1a** (Table S6), thus ruling out the possibility that background reactions contribute to the high FE_H2_. As mentioned earlier, **4** can be detected by IR-SEC both for complex 1a* with an open structure^9^ and **1a** with a closed macrocyclic structure (Figures S10 and S11), making the HCOO^–^/H_2_-generating pathways viable. To account for the change in product selectivity with 1a* and **1a**–**c**, the mechanism for the formation of H_2_, CO, and HCOO^–^ was studied by DFT calculations.

### DFT Mechanistic Studies

The mechanism of CO_2_ reduction starts with the anionic intermediate **2a^–^**, whose lowest energy isomer was selected from AIMD simulations and optimized using static DFT calculations in solvent (Scheme S2). Three scenarios were considered for the formation of the three reaction products (H_2_, CO, and HCOO^–^): two starting with the protonation of the amines (N_M_ and N_S_), which favors the formation of a Mn hydride (**4a**) in an *endo* conformation (see Scheme 4 and Schemes S3 and S4), and one starting with the direct CO_2_ addition or protonation of **2a^–^** in an *exo* conformation (with CO_2_, or H, and the amines in opposite sides, see Scheme S5).

Scheme 4 outlines the reaction energy profiles for the formation of H_2_, CO, and HCOO^–^ considering the protonation of the middle N as the first step, which is the one that provides the lowest energy barrier for the formation of H_2_. This profile has been poised at a calculated potential of −1.60 V vs Fc^+^/Fc, which is the potential required to reduce **1a** to **2a^–^**, as shown in Scheme 3. As shown previously by us,^9^ the amine is protonated by TFE with the assistance of CO_2_ yielding **3a-N_M_** and CF_3_CH_2_OCOO^–^, through an energy barrier of 8.8 kcal mol^–1^. From here, proton transfer to the metal center to form **4a** is preferred over direct CO_2_ addition to the Mn center to form **7a-N_M_** by 1.2 kcal mol^–1^. Furthermore, CO_2_ addition appears to be endergonic and reversible, whereas the proton transfer is exergonic and irreversible. Once the reactive **4a** is generated, there are two competing pathways: (i) CO_2_ insertion into the Mn–H bond leading to the Mn–OCHO formato complex (**5a**) for final HCOO^–^ generation (HCOO^–^ pathway in red)^8,42^ and (ii) protonation of the amine moiety facilitating H_2_ production (H_2_ pathway in green).^22,45^ The energy barrier of CO_2_ insertion (**TS-4**) to form **10a** is 19.2 kcal mol^–1^; **10a**, in which the formate is bonded to Mn by the hydrogen atom (Mn–HCO_2_) is 18.2 kcal mol^–1^ less stable than the one bonded via the oxygen atom (**5a**). Initially, one TS connecting **10a** and **5a** was found at 8.8 kcal mol^–1^, with Mn···H and Mn···O distances of 2.49 and 3.27 Å, respectively. However, upon considering the possibility of connecting **5a** and **2a^+^**, we found **TS-7**, which also connects **10a** and **5a** but with an energy of 2.6 kcal mol^–1^, and longer Mn···H and Mn···O distances (3.14 and 4.04 Å, respectively). An alternative mechanism for the direct formation of **5a** from **3a-N_M_**, as it has been suggested for enzymes,^46−48^ was also considered without success.

Following the H_2_ pathway, the energy difference between **TS-5** (−2.5 kcal mol^–1^) and **4a** (−11.5 kcal mol^–1^), which is 9.0 kcal mol^–1^, shows that the barrier for protonation of the amine to form **6a^+^-N_M_** is lower than that of CO_2_ insertion. Liberation of H_2_, however, requires the proton transfer from the amine to the hydride, which has an energy barrier of 15.1 kcal mol^–1^ (**TS-8**). Overall, the formation of H_2_ and HCOO^–^ has similar barriers, as shown by the energies of **TS-8** (6.2 kcal mol^–1^) and **TS-4** (7.7 kcal mol^–1^), respectively, with generation of H_2_ being slightly preferred. This result is consistent with the experimental findings that H_2_ formation is preferred over HCOO^–^ (FE_H2_ = 66% vs FE_HCOO–_ = 29%). On the other hand, the formation of CO (blue pathway) continues by proton transfer (**TS-6**) from the Mn–CO_2_ intermediate (**7a-N_M_**) to form a hydroxyl–carbonyl complex, **8a**, which is a well-known intermediate in CO_2_ to CO reduction processes.^10,49^ Subsequent protonation of **8a** (1.1 kcal mol^–1^) followed by proton transfer, forming a tetracarbonyl adduct and final liberation of CO and H_2_O (4.0 kcal mol^–1^), has a barrier of 10 kcal mol^–1^ but is endergonic by ∼3 kcal mol^–1^. The CO pathway has consistently higher energies when compared to the H_2_ and HCOO^–^ pathways.

Another interesting observation from Scheme 4 is that the H_2_ pathway is reversible relative to **4a**. Since the reaction takes place under electrochemical conditions, **4a** can be reduced to **4a^–^**, with a calculated redox potential of −1.87 V vs Fc^+^/Fc (Scheme 4), which is consistent with the appearance of the high current density increment in the cyclic voltammograms under CO_2_ (−2.10 V vs Fc^+^/Fc, Figure 4), where **4a^–^** is generated as the bifurcation between the H_2_ and HCOO^–^ competing pathways. Hence, the pathways for HCOO^–^ and H_2_ formation were also calculated from this reduced intermediate, **4a^–^** (Scheme 5). It is seen that the energy barriers relative to **4a^–^** for H_2_ formation (**TS-15** = 10.4 kcal mol^–1^) and CO_2_ insertion (**TS-13** = 14.7 kcal mol^–1^) are lowered by 7.3 and 4.5 kcal mol^–1^, respectively, when compared to Scheme 4, where these barriers are 17.7 (**TS-8**) and 19.2 kcal mol^–1^ (**TS-4**) relative to **4a**. Consistently, the barrier for H_2_ formation is lowered more than that for HCOO^–^ generation, leading H_2_ to be the dominant product. Additionally, it was seen that product formation in Scheme 5 was exergonic and irreversible, pointing to a reaction that takes place via **4a^–^**. This finding is in line with the high overpotential pathway reported for complex 1a*.^9^

The results shown in Scheme 4 were compared with those considering the protonation of the side amine (Schemes S3 and S4). The main differences observed are as follows: (i) Although the protonation of the side amine is thermodynamically favored over the middle amine (Δ*G* = −2.5 and 1.0 kcal mol^–1^, respectively), proton transfer to the Mn center is preferably assisted by the middle amine, with a lower energy barrier (Δ*G*^‡^ = 21.2 kcal mol^–1^ vs Δ*G*^‡^ = 13.6 kcal mol^–1^)—this finding is consistent with the AIMD simulations, which showed that the middle proton gets closer to the Mn center than the protons on the sides; (ii) the barrier for CO_2_ coordination (CO pathway, **TS-18**) is lower than that for the amine-to-Mn proton transfer (H_2_ pathway, **TS-17**) when the side amine is protonated, whereas the opposite holds true for the middle amine; (iii) after the formation of **4a**, both the middle and side amines have similar barriers for the H_2_ and HCOO^–^ pathways, with a slight preference for the former.

Finally, we compared the energy profiles shown in Scheme 4 with those obtained with the *exo* isomer formed by the rotation of the Mn(CO)_3_ core in the AIMD calculations (Scheme S5). Compared with the *endo* isomer, where the formation of **4a** is assisted by the N_M_ amine, the *exo* isomer must go through a direct protonation of the metal to form **4a_ex_** with an energy barrier of 17.2 kcal mol^–1^, which is 9.7 kcal mol^–1^ lower than the direct protonation of the metal in the *endo* isomer (Scheme S9). Conversely, for the CO pathway, a direct addition of CO_2_ to Mn takes place in the *exo* isomer with an energy barrier of 14.9 kcal mol^–1^ (**TS-27**, Scheme S5), which is favored over the direct addition of CO_2_ to Mn in the *endo* isomer by 3.2 kcal mol^–1^ (**TS-33**, Scheme S6). As a result, while the formation of **4a** (Δ*G*^‡^ = 14.6 kcal mol^–1^) is favored over direct CO_2_ addition (Δ*G*^‡^ = 18.1 kcal mol^–1^) in the *endo* case, the opposite happens in the *exo* profile, with a preference for CO_2_ addition (Δ*G*^‡^ = 14.9 kcal mol^–1^) over hydride formation (Δ*G*^‡^ = 17.2 kcal mol^–1^). These results suggest that the cyclic ligand, once protonated, forms a cagelike structure that protects the Mn center from CO_2_ addition, hindering the formation of CO. Despite this, a minor concentration of CO is formed due to the addition of CO_2_ in the side opposite to the ligand before protonation (*exo* isomer). This mechanistic picture is in line with the results obtained experimentally in the CPE measurements.

The preference for H_2_ formation over HCOO^–^ can be ascribed to the same effect. In **4a**, the cagelike structure of the ligand hinders the addition of CO_2_. Indeed, the opposite selectivity is observed with the open system 1a*. To corroborate this hypothesis, we compared the energy barriers obtained for the protonation of hydride **4a** and CO_2_ addition for **1a** and 1a* (Scheme S7). These calculations showed that both processes have lower energy barriers in the open system 1a*. The protonation of 4a* has an energy barrier that is 3.9 kcal mol^–1^ lower than that of complex **4a,** while the CO_2_ addition barrier is significantly lower (by 14.5 kcal mol^–1^), consistent with a change in selectivity from mainly HCOO^–^ for 1a* to mainly H_2_ for **1a** (Figure 5). This result was further supported by controlled potential electrolysis of 1a* and **1a** in the presence of 2.0 M TFE under Ar atmosphere (i.e., without CO_2_), which yielded H_2_ as the only product. Similar results were obtained for **1b** and **1c** as electrocatalysts under these experimental conditions (Table S7). Hence, the less efficient production of HCOO^–^ by **1a–c** is, most likely, attributed to the low accessibility of CO_2_. The macrocyclic ligand around the metal center induces steric hindrance for the insertion of CO_2_ into the metal hydride. At the same time, protons can still be delivered to it by the amine-bearing ligands, explaining the preference for H_2_ generation over CO_2_ reduction. Along this line, it would be expected that the hindrance associated with CO_2_ insertion to hydride will be incremented for **1c** when a more rigid ring is formed by removing the middle nitrogen atom. Indeed, this is reflected experimentally in the lowest FE for HCOO^–^ (3%) obtained for **1c** among **1a**–**c**.

## Conclusions

Rational design of molecular complexes is crucial for the development of highly selective and efficient catalysts, but the factors affecting these parameters are not uncovered completely yet. This work addressed how the geometric structure of Mn-based complexes affects the competing protonation and CO_2_ insertion steps in the electrocatalytic CO_2_ reduction reaction. Depending on the sequence of these steps, different catalytic intermediates are formed, leading to the formation of CO, when CO_2_ reacts directly with the metal center, or HCOO^–^/H_2_ if a metal hydride is formed during the first step.

To investigate how spatial geometry around the catalytic site affects the formation of these products, three different **Mn(bpy-R)(CO)_3_Br** complexes (**1a–c**, Figure 1) were designed and synthesized. Via a combined theoretical and experimental study, we found several parameters that influence the selectivity of the catalysts. First, *ab initio* molecular dynamics simulations revealed that the three carbonyl groups rotate freely around the metal center after a 2e^–^ reduction of the complex, leaving the molecular structure more flexible than first anticipated. The outcome of the CO_2_ reduction reaction is thus dependent on the stability of the two major conformers, where the catalytic site points either in the same (*endo*) or in the opposite direction (*exo*) of the macrocyclic ligand. In the former case, amine protonation and subsequent proton transfer to the metal center are preferred, favoring the formation of a metal hydride, whereas CO_2_ attacks directly from the unhindered side in the latter case to generate a manganese hydroxyl–carbonyl complex, the key intermediate for CO production. Hence, the reactivity cannot be rationalized based on the experimentally derived crystal structure due to the fluxional behavior of the carbonyl groups, which to the best of our knowledge has not been reported previously for CO_2_-reducing Mn tricarbonyl complexes. A second factor that affects the mechanism and therefore also the product distribution is the position of the amine functionalities on the macrocyclic ligand. In this respect, *ab initio* molecular dynamics simulations showed that the distance between Mn and the protonated amines is shortest for the middle amine of complex **1a**, lowering the energy barrier for proton transfer to the metal center compared to the case where the side-amine was protonated. Thus, hydride formation can be enhanced by tuning the distance between the metal center and the proton shuttles. Finally, the accessibility of CO_2_ as well as protons to the active site should always be kept in consideration, as it is crucial for switching reaction pathways from one to another.

These computational findings align well with the experimental results, where H_2_ was the dominant product for all three catalysts (**1a**, **1b**, and **1c**), while only small amounts of CO and HCOO^–^ were produced due to disfavored CO_2_ addition reactions before and after hydride formation, respectively. Formation of HCOO^–^ was further suppressed with complex **1c**, where the linker was shortened by one atom, thus limiting CO_2_ insertion and favoring a second protonation after formation of the hydride. The observed product distribution is furthermore in contrast with our previous work, where complex 1a* with a similar but “open” amine ligand produced HCOO^–^ as the dominant product, substantiating the importance of considering the geometric structure when designing new molecular catalysts.

## Supporting Information Available

Experimental details; X-ray crystallographic data; supporting figures (S1–S19), schemes (S1–S9), and tables (S1–S9); computational details; synthesis procedures and spectroscopic data (PDF)
X-ray crystallographic data for **1a**–**c** in CIF format and Cartesian coordinates (XYZ)

## Supplementary Material

cs2c05951_si_001.pdf

cs2c05951_si_002.xyz

## Notes

Computational results are available in the ioChem-BD repository^50^ and can be accessed via https://doi.org/10.19061/iochem-bd-6-192.

## Abbreviations

AIMD: *ab initio* molecular dynamics
CPE: controlled potential electrolysis
DFT: density functional theory
HER: hydrogen evolution reaction
IR-SEC: infrared spectroelectrochemistry
STAB–H: sodium triacetoxyborohydride
TFE: 2,2,2-trifluoroethanol
