# Supplementary material for: Exploring the Parameters Controlling Product Selectivity in Electrochemical CO2 Reduction in Competition with Hydrogen Evolution Employing Manganese Bipyridine Complexes
Source: ACS Catal. 2023 Feb 16;13(5):3109–19. doi: 10.1021/acscatal.2c05951 (PMC9990071; doi:10.1021/acscatal.2c05951)
Supplement: Supplementary file 1 — cs2c05951_si_001.pdf [file cs2c05951_si_001.pdf]

Supporting Information

# Exploring the Parameters Controlling Product Selectivity in Electrochemical $CO_2$ Reduction in Competition with Hydrogen Evolution Employing Manganese Bipyridine Complexes

Wanwan Hong,[a,‡] Mahika Luthra,[b,‡] Joakim B. Jakobsen,[a,ǁ,‡] Monica R. Madsen,[a,†] Abril C. Castro,[b] Hans Christian D. Hammershøj,[a] Steen U. Pedersen,[c] David Balcells,[b] Troels Skrydstrup,[a,d,*] Kim Daasbjerg,[d,*] and Ainara Nova[b,e,*]

[a]Carbon Dioxide Activation Center (CADIAC), Interdisciplinary Nanoscience Center, Department of Chemistry, Aarhus University, Gustav Wieds Vej 14, 8000 Aarhus C, Denmark
[b]Hylleraas Centre for Quantum Molecular Sciences, Department of Chemistry, University of Oslo, 0315, Oslo, Norway
[c]Interdisciplinary Nanoscience Center, Department of Chemistry, Aarhus University, Langelandsgade 140, 8000 Aarhus C, Denmark
[d]Novo Nordisk Foundation (NNF) $CO_2$ Research Center, Interdisciplinary Nanoscience Center, Department of Chemistry, Aarhus University, Gustav Wieds Vej 10C, 8000 Aarhus C, Denmark
[e]Center for Materials Science and Nanotechnology, Department of Chemistry, University of Oslo, 0315, Oslo, Norway
[ǁ]Present address is Department of Chemistry, Technical University of Denmark, 2800 Kgs. Lyngby, Denmark.
[†]Present address is Unisense A/S, Langdyssen 5, 8200 Aarhus N, Denmark.
[‡]These authors contributed equally.
*Correspondence to ts@chem.au.dk (T.S.), kdaa@chem.au.dk (K.D.), and a.n.flores@kjemi.uio.no (A.N.)

## Table of Contents

1. General Considerations ........ S3
1.1 Cyclic Voltammetry ........ S3
1.2 Controlled Potential Electrolysis ........ S4
1.3 Infrared Spectroelectrochemistry ........ S4
2. X-Ray Crystallographic Data ........ S5
Complex 1a ........ S5
Complex 1b ........ S6
Complex 1c ........ S7
3. Computational Methods and Details ........ S8
3.1 *Ab-initio* Molecular Dynamics (AIMD) Simulations ........ S8
3.2 Static DFT Calculations ........ S8
3.3 Computational Study of Reduction Potentials ........ S8
4. Figures, Schemes, and Tables ........ S9
5. Trace Crossing (Figures 4, S3, and S4) ........ S32
6. Residual Water in Electrochemical System ........ S33
6.1 Quantification of Residual Water in Electrochemical Cell ........ S33
6.2 Cyclic Voltammograms Recorded in Dried Electrolyte ........ S33
6.3 Control Electrolysis with Residual Water as Proton Source under $CO_2$ ........ S34

7. Synthesis of Relevant Materials .......... S36
7.1 Synthesis of Manganese Complexes .......... S36
7.2 Synthesis of Ligands .......... S39
7.3 Synthesis of Starting Materials .......... S40
8. References .......... S43
9. Spectroscopic Data .......... S46

## 1. General Considerations

Commercially available reagents and analytical grade solvents were purchased from Fluorochem, TCI Chemicals, or Sigma-Aldrich and used without further purification unless otherwise stated. $CO_2$ with a purity of 99.999% was supplied by Air Liquide. $Bu_4NBF_4$ was synthesized by mixing $NaBF_4$ (98%) and $Bu_4NHSO_4$ in a molar ratio of 1:1 and dried on a Schlenk line before use. Anhydrous solvents were collected from an MBRAUN MB SP-800 purification system and further dried over 3 Å molecular sieves. Air-sensitive reactions were carried out using standard Schlenk and vacuum techniques or were performed in an argon-filled glovebox. Light-sensitive reactions were conducted in amber glassware or shielded from light by aluminum foil.

NMR spectra were recorded on a Bruker Ascend 400 spectrometer running at 400 MHz for $^1H$ and 101 MHz for $^{13}C$. Chemical shifts ($\delta$) are reported in parts per million (ppm) relative to residual solvent signals.[S1] Spectroscopic data from $^1H$-NMR spectroscopy are reported as follows: Chemical shift in ppm (multiplicity, coupling constant *J* (Hz), integration intensity). The multiplicity is abbreviated as follows: s, singlet; d, doublet; t, triplet; q, quartet; p, quintet; m, multiplet; bs, broad signal. $^{13}C$ NMR spectra were recorded in broadband decoupled mode.

HRMS were recorded on a Bruker Maxis Impact mass spectrometer using electrospray ionization ($ESI^+$). Isotope patterns were analyzed using Data Analysis (v4.1) by Bruker Daltonic GmbH to give the m(Sigma) values. ATR FT-IR was recorded on a Perkin Elmer Spectrum Two instrument.

Analytical thin-layer chromatography (TLC) was performed using pre-coated aluminum-backed plates (Merck Kieselgel 60 F254) or pre-coated glass-backed plates (Supelco Aluminum oxide 60 $F_{254}$, basic) and visualized by UV irradiation, $I_2$, or $KMnO_4$ stain. Flash column chromatography (FCC) was performed using silica gel (Silica gel 60, 230–400 mesh, Sigma-Aldrich) or basic alumina (Aluminum oxide 60, activity I, 70–230 mesh ASTM, Millipore).

Cyclic voltammetry and controlled potential electrolysis were conducted with a CH Instruments (660B) potentiostat. A Nicolet 6700 (Thermo Fisher Scientific) instrument was applied to record the solution state IR spectra. A CH Instrument (660B) was used to control and monitor the electrochemical processes during the Infrared spectroelectrochemistry (IR-SEC) experiments.

Gaseous products released into the headspace during the controlled potential electrolysis ($H_2$ or CO) were detected on an Agilent 7890B Gas Chromatograph equipped with TCD and FID detectors and a Agilent CP7429 split column. Quantification of gas products were calculated from calibration curves prepared by injecting 250 µL of calibration gases consisting of known concentrations of $H_2$ and CO mixed with $CO_2$. Formate was identified on a Thermo Scientific Dionex ICS-1100 ion chromatography equipped with a Dionex IonPac AS10 column for anion separation. Quantification was accomplished from a calibration curve by measuring on known concentrations of sodium formate.

Single crystal X-ray crystallographic data was obtained using an Oxford Diffraction Supernova instrument equipped with a Mo micro-focus X-ray source, an Atlas charge-coupled device detector, and a four-circle goniometer. The crystal was cooled to 100(1) K using an Oxford Cryosystems liquid nitrogen Cryostream device.

### 1.1 Cyclic Voltammetry

All cyclic voltammograms were recorded using a standard three-electrode setup in a single-compartment electrochemical cell and with 0.1 M $Bu_4NBF_4$/MeCN as electrolyte. Ohmic drop was compensated by the positive feedback compensation applied via the potentiostat. The working electrode (WE) was a homemade glassy carbon (GC) disk electrode (Sigradur G, HTW, *d* =1 mm) embedded in epoxy resin. A nonaqueous Ag/AgI electrode was applied as the pseudo reference electrode (RE), while a platinum wire was used as the counter electrode (CE). Potentials were calibrated by the

ferrocenium/ferrocene ($Fc^+$/Fc) redox couple at the end of each experiment by adding Fc into the cell as internal standard. A sweeping rate of 0.1 V $s^{-1}$ was employed unless otherwise noted.

### 1.2 Controlled Potential Electrolysis

Bulk electrolysis experiments were conducted in a three-electrode system. The amber electrolytic cell consisted of two compartments separated by a glass frit and connected by a bridge at the upper part to allow gas phases to equilibrate. Carbon paper (Toray Paper 060 from FuelCellStore) was applied as WE and wrapped by Teflon tape to demarcate the active electrode area to be 0.5 cm × 0.5 cm × 2 in the catholyte. A leak-free saturated Ag/AgCl electrode served as RE and was attached to the WE by Teflon tape to keep the same distance in all experiments. A Pt mesh was employed as CE.

Each chamber contained a magnetic stir bar and 0.2 M $Bu_4NBF_4$/MeCN. Either TFE (2.0 M) or *i*PrOH (1.0 M) was added to both chambers as proton source while the catalyst was only added to the cathodic chamber in 1.5 mM concentration. The final volume in each chamber was 4.8 mL. The WE/RE assembly was placed in the cathodic chamber, while the CE was placed in the other chamber. The cell was sealed and deoxygenated by purging $CO_2$ for 10–15 min, after which the $CO_2$ was withdrawn, and the electrochemical cell was connected to the potentiostat. The cell was placed into a water bath at room temperature during the 1 h electrolysis to avoid the overheating of the glass frit. The solution was constantly stirred during the experiment.

### 1.3 Infrared Spectroelectrochemistry

IR-SEC was performed in a homemade cell, described in detail in our previous work.[S2] A GC disk ($d$ = 4 mm) was used as WE surrounded by a Pt wire as CE. A commercial leak-free Ag/AgCl electrode was equipped as RE positioned between the WE and CE. Spectroelectrochemical experiments were carried out in 0.1 M $Bu_4NBF_4$/MeCN containing 1.5 mM catalyst and either 2.0 M TFE or 1.0 M *i*PrOH. Background spectra were obtained in solutions identical to the ones being examined but in the absence of catalyst. 50%-saturation of $CO_2$ was prepared by purging with a 1/1 gas mixture of Ar and $CO_2$ using two variable area flow meters (Key Instruments). The sweeping rate for cyclic voltammetric experiments in this part is 0.025 V $s^{-1}$. A series of IR spectra were recorded during the voltammogram, making it possible to study the potential-dependent appearance/disappearance of the key intermediates and products. If necessary, absorbance was corrected by setting it to zero at 2100 $cm^{-1}$ since, in general, no peaks appear at this point.

## 2. X-Ray Crystallographic Data

Intensities were empirically corrected for absorption using SCALE3 ABSPACK implemented in CrysAlisPRO.[S3] The unit cell parameters were determined, and the Bragg intensities were integrated using CrysAlisPRO. The structure was solved and refined with SHELXS and SHELXL, respectively, in Olex2.[S4–S6]

### Complex 1a

A single crystal suitable for X-ray crystallographic analysis was obtained by performing vapor diffusion of pentane into a concentrated solution of **1a** in THF at –35 °C.

| Item | Value |
|---|---|
| Molecular formula | $C_{36}H_{39}BrMnN_5O_3$ |
| Formula weight | 724.57 |
| Crystal system | triclinic |
| Space Group | P -1 |
| a (Å) | 9.5678(3) |
| b (Å) | 10.9055(6) |
| c (Å) | 17.6840(7) |
| α (°) | 72.077(4) |
| β (°) | 87.466(3) |
| γ (°) | 74.921(4) |
| Volume (Å$^3$) | 1693.89(14) |
| Z | 2 |
| T (K) | 100 |
| ρ (g cm$^{-1}$) | 1.421 |
| λ (Å) | 0.71073 |
| μ (mm$^{-1}$) | 1.612 |
| # measured refl | 25988 |
| # unique refl | 9249 |
| $R_{int}$ | 0.0734 |
| # parameters | 418 |
| R(F$^2$), all refl | 0.0822 |
| $R_w$(F$^2$), all refl | 0.1132 |
| Goodness of fit | 1.022 |

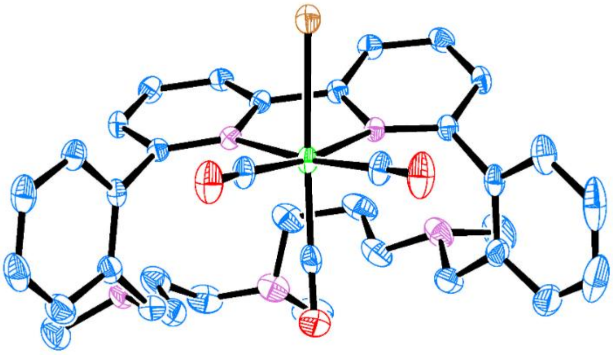

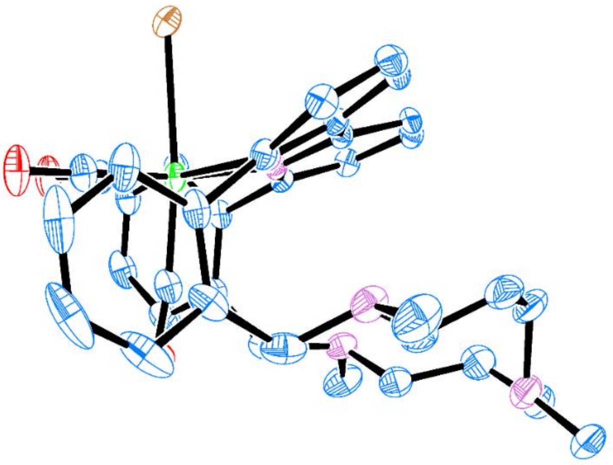

CCDC 2182168

## Complex 1b

A single crystal suitable for X-ray crystallographic analysis was obtained by performing vapor diffusion of $Et_2O$ into a solution of **1b** in $CH_2Cl_2$ at –20 °C.

| Item | Value |
|---|---|
| Molecular formula | $C_{35}H_{36}BrMnN_4O_4$ |
| Formula weight | 711.53 |
| Crystal system | triclinic |
| Space Group | P-1 |
| a (Å) | 9.6210(2) |
| b (Å) | 10.7982(2) |
| c (Å) | 17.5687(4) |
| α (°) | 73.287(2) |
| β (°) | 81.045(2) |
| γ (°) | 70.138(2) |
| Volume (Å$^3$) | 1640.71(6) |
| Z | 2 |
| T (K) | 100 |
| ρ (g cm$^{-1}$) | 1.440 |
| λ (Å) | 0.71073 |
| μ (mm$^{-1}$) | 1.664 |
| # measured refl | 85662 |
| # unique refl | 9432 |
| $R_{int}$ | 0.0450 |
| # parameters | 408 |
| R(F$^2$), all refl | 0.0410 |
| $R_w$(F$^2$), all refl | 0.0742 |
| Goodness of fit | 1.033 |

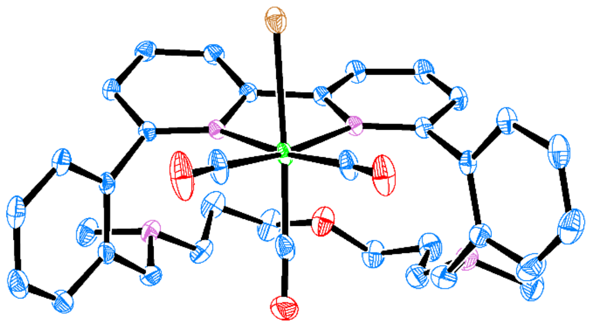

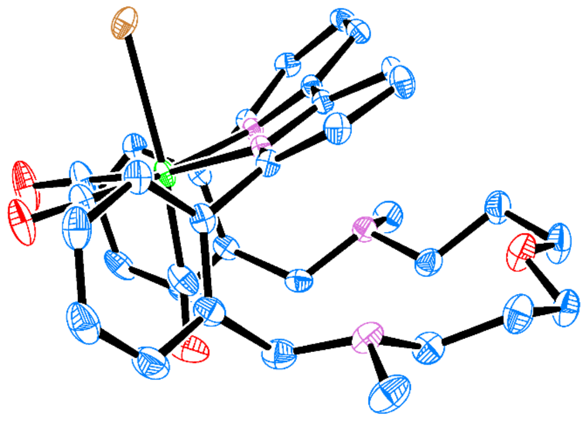

CCDC 2182240

## Complex 1c

A single crystal suitable for X-ray crystallographic analysis was obtained by performing vapor diffusion of $Et_2O$ into a solution of **1c** in $CH_2Cl_2$ at –20 °C.

| Item | Value |
|---|---|
| Molecular formula | $C_{35}H_{36}BrMnN_4O_3$ |
| Formula weight | 695.53 |
| Crystal system | triclinic |
| Space Group | P-1 |
| a (Å) | 9.4615(4) |
| b (Å) | 10.5636(6) |
| c (Å) | 18.0578(9) |
| α (°) | 77.002(4) |
| β (°) | 88.120(4) |
| γ (°) | 68.470(4) |
| Volume (Å$^3$) | 1633.42(15) |
| Z | 2 |
| T (K) | 100 |
| ρ (g cm$^{-1}$) | 1.414 |
| λ (Å) | 0.71073 |
| μ (mm$^{-1}$) | 1.668 |
| # measured refl | 25359 |
| # unique refl | 8790 |
| $R_{int}$ | 0.0450 |
| # parameters | 399 |
| R(F$^2$), all refl | 0.0532 |
| $R_w$(F$^2$), all refl | 0.0902 |
| Goodness of fit | 1.032 |

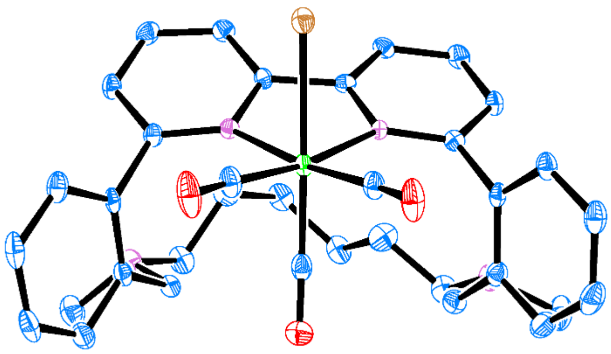

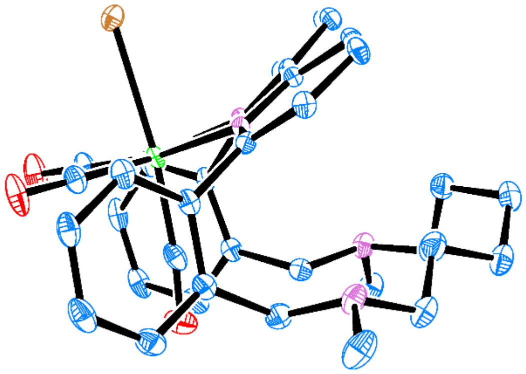

CCDC 2182232

# 3. Computational Methods and Details

## 3.1 *Ab-initio* Molecular Dynamics (AIMD) Simulations

AIMD[S7] simulations for the macrocyclic system [**2a**$^{-}$, **3a-N$_{S1}$**, **3a-N$_{S2}$**, and **3a-N$_{M}$**] were run in the gas phase according to the Born-Oppenheimer approximation using the *CP2K* program package.[S8] The simulations were performed using the Kohn-Sham DFT with PBE exchange-correlation functional,[S9] in a mixed DZVP Gaussian[S10] and auxiliary plane-wave (200 Ry cutoff) basis set. The initial model system of each intermediate, which consist of a cubic box of 25.0 Å edge, was relaxed using a microcanonical (NVE) ensemble until an average temperature of 298 K was reached. After equilibration, the simulation was run using a canonical (NVT) ensemble with a temperature of 298 K maintained with the CSVR algorithm.[S11] Core electrons were described using pseudopotentials of the Goedecker-Teter-Hutter type.[S12] Dispersion forces were considered using Grimme's D3 model.[S13] The trajectories were extended up to 25 ps with a time step of 0.25 fs.

AIMD simulations for complex **2a**$^{-}$ were also performed in an explicit acetonitrile ($CH_3CN$) solvent. The initial model system was created using the *PACKMOL* package[S14] which consists of complex **2a**$^{-}$ (optimized at the TPSSh level) surrounded by 52 $CH_3CN$ molecules in a cubic box of 16 Å edge to reproduce the appropriate density of 0.786 g $mL^{-1}$. The simulation cell was treated under periodic boundary conditions. These simulations were performed using the same methodology as in the gas-phase simulations. Similar behavior was observed in both gas-phase and explicit solvent simulations of complex **2a**$^{-}$ (Figure S1). Hence, the analysis of the protonated species (**3a-N$_{M}$**, **3a-N$_{S1}$**, and **3a-N$_{S2}$**) was carried out only in gas-phase to reduce computational cost.

## 3.2 Static DFT Calculations

To investigate the mechanism of $CO_2$ reduction, DFT calculations[S15] were performed using the Gaussian 16 program, Revision C.01.[S16] The standard reduction potentials were calculated relative to the $Fc^+$/Fc couple. The methodology leading to a better agreement with the experimental values (Table S8) was TPSSh/def2SVP.[S17–S19] Hence, this method was used for all the calculations. Single point energy calculations were performed with the TPSSh functional and def2-TZVP basis set,[S18] which is a triple-zeta basis set for the metal Mn in combination with the def2-TZVPD basis set[S18] for the rest of the atoms. All geometries were optimized including the D3 empirical dispersion correction from Grimme,[S20] which was also included in the computation of the energies. Frequency calculations[S21,S22] were performed on the optimized geometries to verify that the geometries correspond to minima or first-order saddle points (transition states) on the potential energy surface and to compute zero-point energies and thermal corrections for the reported free energies at 298 K. The effect of the solvent was evaluated in all the calculations using the continuum solvation model SMD (Solvation Model based on Density)[S23] with the settings for acetonitrile. The Gibbs free energies of all species are calculated at a standard state of 1M (sol) at 298 K.

## 3.3 Computational Study of Reduction Potentials

The standard reduction potentials were calculated as follows:

For a reduction process starting from A,

$$A(sol) + n\,e^{-}\,(g) \rightarrow A^{n-}\,(sol)$$

where n represents the number of electrons involved and $A^{n-}$ is the $n^{th}$ reduced form of A.

The reduction potential relative to the ferrocenium/ferrocene couple was calculated as

$$E^0(A/A^{n-})\ (\text{vs } Fc^+/Fc) = E^0(Fc^+/Fc) - \Delta G^0(A/A^{n-})/nF$$

where $\Delta G^0(A/A^{n-})$ is the change of Gibbs free energy for the reduction of A(aq); and F is Faraday constant (23.0605 kcal $mol^{-1}$ $V^{-1}$).

## 4. Figures, Schemes, and Tables

### Table S1. Relativistic Calculation of NMR Chemical Shifts

$^{1}H$ and $^{13}C$ NMR shielding constants were calculated with the ADF program[S24] using the KT2[S25] functional in conjunction with the ET-pVQZ[S26] all electron basis set. The conductor-like screening model (COSMO) [S27–S29] for simulating bulk solvation in dichloromethane (for $^{1}H$ NMR) or DMSO (for $^{13}C$ NMR). Scalar (SR) and spin-orbit (SO) relativistic effects were included at the two-component level using the zeroth-order regular approximation Hamiltonian (ZORA).[S30–S34] The $^{1}H$ and $^{13}C$ chemical shifts were calculated relative to tetramethylsilane (TMS) and using the GIAO method.[S35]

**Table S1. $^{1}H$ and $^{13}C$ NMR Chemical Shifts (in ppm) for Complex 1a Calculated at the ZORA-KT2/ET-pVQZ Level.**

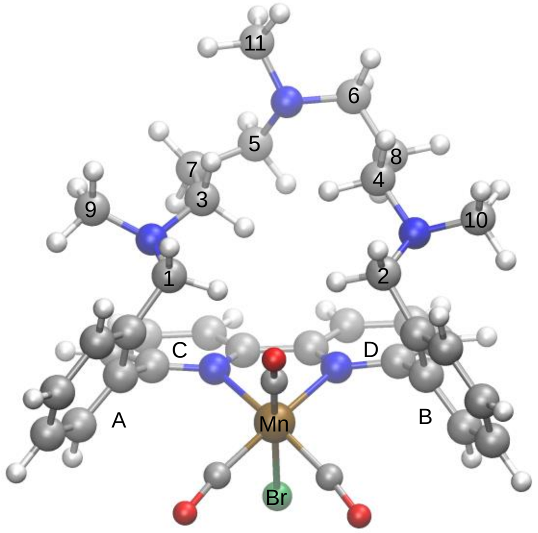


| | $^{1}H$ NMR chemical shifts[a] | $^{13}C$ NMR chemical shifts[b] |
|---|---|---|
| $C_6H_4$- [A] | 7.50, 7.57, 7.63, 7.88 | 128.4, 129.2, 130.4, 130.5, 141.3, 142.3 |
| $C_6H_4$- [B] | 7.49, 7.59, 7.62, 7.82 | 128.4, 129.8, 130.2, 130.7, 140.8, 142.6 |
| $NC_5H_3$- [C] | 7.49, 7.93, 8.28 | 120.3, 126.8, 136.4, 156.8, 166.4 |
| $NC_5H_3$- [D] | 7.57, 7.95, 8.35 | 120.5, 128.5, 135.5, 155.9, 164.8 |
| $NC(1)H_2$ | 3.05, 4.80 | 71.2 |
| $NC(2)H_2$ | 3.25, 4.40 | 70.4 |
| $NC(3)H_2$ | 1.83, 2.49 | 67.2 |
| $NC(4)H_2$ | 1.77, 2.88 | 63.4 |
| $NC(5)H_2$ | 1.77, 2.45 | 55.6 |
| $NC(6)H_2$ | 2.23, 2.37 | 59.8 |
| $C(7)H_2$ | 0.56, 1.85 | 30.8 |
| $C(8)H_2$ | 1.07, 1.29 | 30.7 |
| $NC(9)H_3$ | 1.70 ($CH_3$-) | 38.4 |
| $NC(10)H_3$ | 1.78 ($CH_3$-) | 40.0 |
| $NC(11)H_3$ | 2.14 ($CH_3$-) | 43.7 |

[a]The NMR shifts are reported relative to TMS ($\delta$ = 31.1 ppm). Values calculated using the COSMO model for $CH_2Cl_2$. [b]The NMR shifts are reported relative to TMS ($\delta$ = 186.7 ppm). Values calculated using the COSMO model for DMSO.

**Scheme S1. DFT-Calculated Thermodynamic Energies for the Isomers of 1a, 1b, and 1c.**

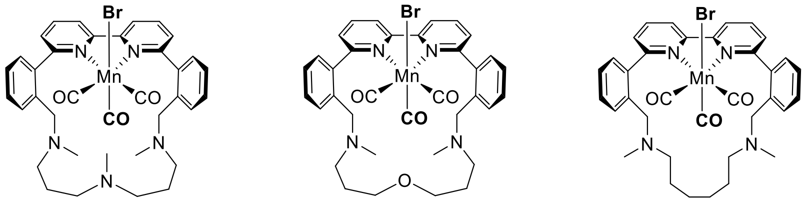


**1a-*exo*** | **1b-*exo*** | **1c-*exo***

**0.0 kcal mol$^{-1}$** | **0.0 kcal mol$^{-1}$** | **0.0 kcal mol$^{-1}$**

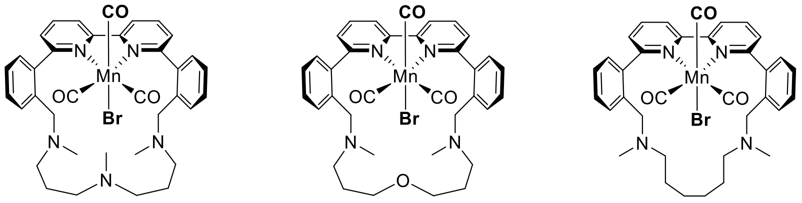


**1a-*endo*** | **1b-*endo*** | **1c-*endo***

**8.4 kcal mol$^{-1}$** | **8.2 kcal mol$^{-1}$** | **7.4 kcal mol$^{-1}$**

## Figure S1 and Table S2. Conformational Analysis for 2a⁻ and 3a-$N_X$ Using AIMD Simulations

AIMD simulations of complex **2a⁻** are consistent in both gas-phase and explicit solvation in acetonitrile. The time-evolution of the Mn···N bond distances involving the three amine N atoms of the ligand ($N_{S1}$, $N_{S2}$, and $N_M$) shows similar results in both cases, with average values of 4.61 ($N_{S1}$), 5.29 ($N_{S2}$) and 6.74 ($N_M$) for gas-phase and 4.56 ($N_{S1}$), 5.43 ($N_{S2}$), and 6.95 ($N_M$) for solvent-phase simulations **(**Table S2**)**. Likewise, a rotation of the CO ligands around the Mn(CO)$_3$ core is observed, where the *exo* form exists in the range of Φ = 65.4° to 160° and flips to the *endo* form in the range of Φ = –50° to –175° **(**Figure S1).

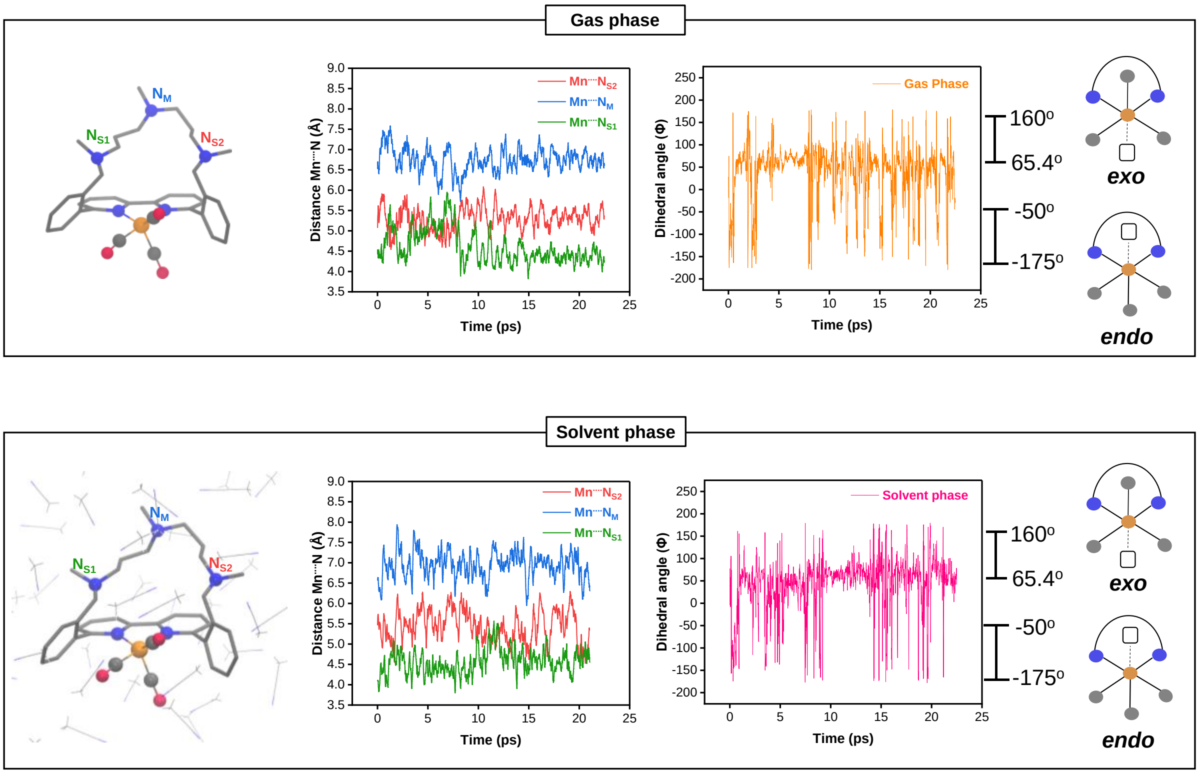


**Figure S1.** Time-evolution of the Mn···N distances (Å) and dihedral angle between axial CO and Mn-bpy for complex **2a⁻**, under (top) gas-phase and (bottom) explicit solvation in acetonitrile.

**Table S2. Analysis of Bond Distances (in Å) between Mn···N$_X$ and Mn···H$_X$ (where X = S1, S2, and M) for the Non-protonated Species 2a$^-$ (Gas-phase and Solvent-phase) and the Protonated Species 3a-N$_X$ (gas-phase) through AIMD Simulations.**

| | Gas Phase **2a$^-$** **(Mn···N$_X$)** | | | Gas Phase **3a-N$_X$** **(Mn···N$_X$)** | | | Gas Phase **3a-N$_X$** **(Mn···H$_X$)** | | | Solvent phase **2a$^-$** **(Mn···N$_X$)** | | |
|---|---|---|---|---|---|---|---|---|---|---|---|---|
| **X** | S1 | S2 | M | S1 | S2 | M | S1 | S2 | M | S1 | S2 | M |
| **Minimum** | 3.82 | 4.54 | 5.72 | 3.36 | 3.29 | 3.08 | 2.30 | 2.45 | 1.87 | 3.79 | 4.52 | 5.94 |
| **Maximum** | 5.95 | 6.08 | 7.58 | 4.98 | 5.59 | 3.86 | 5.39 | 4.76 | 3.03 | 5.56 | 6.29 | 7.93 |
| **Amplitude** | 2.13 | 1.54 | 1.86 | 1.62 | 2.31 | 0.78 | 3.09 | 2.31 | 1.16 | 1.77 | 1.77 | 1.99 |
| **Average** | 4.61 | 5.29 | 6.74 | 4.14 | 4.19 | 3.39 | 3.58 | 3.68 | 2.38 | 4.56 | 5.43 | 6.95 |

**Figure S2. Molecular Orbitals Visualization of 2a$^-$ and 3a-N$_M$.**

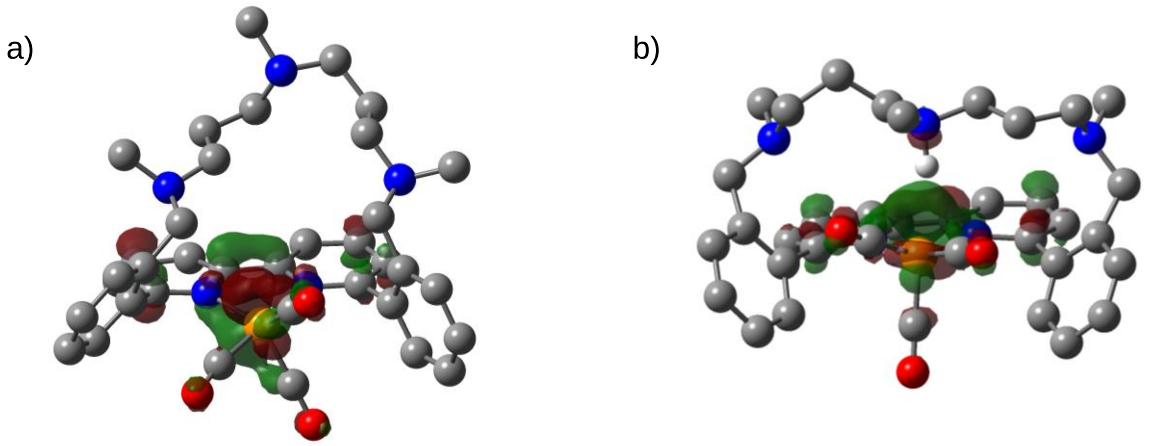


**Figure S2.** Schematic representation for the HOMO orbitals for (a) **2a$^-$** system and (b) protonated system at the middle nitrogen (**3a-N$_M$**). (Isovalue: 0.05)

**Figure S3-S5. Cyclic voltammograms of 1a, 1b, and 1c.**

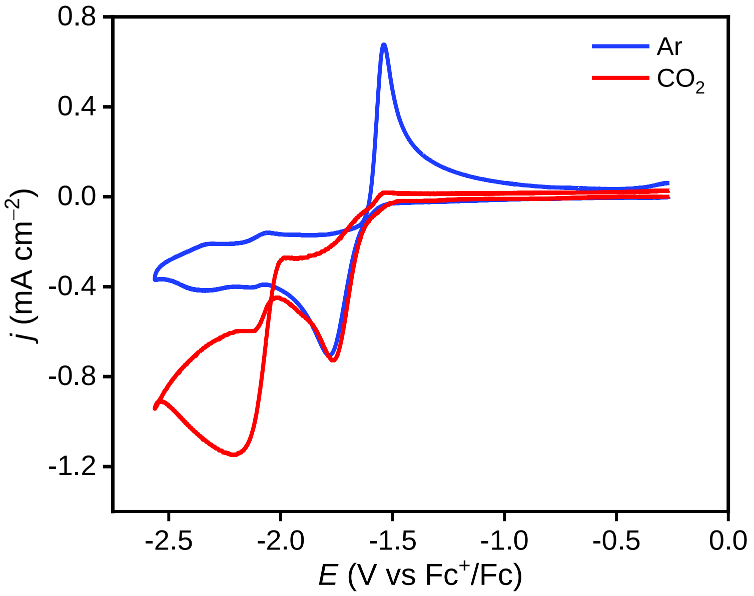


**Figure S3.** Cyclic voltammograms recorded on 1.5 mM **1b** at a GC electrode (diameter = 1 mm) using $\nu = 0.1$ V s$^{-1}$ in Ar- or $CO_2$-saturated 0.1 M $Bu_4NBF_4$/MeCN.

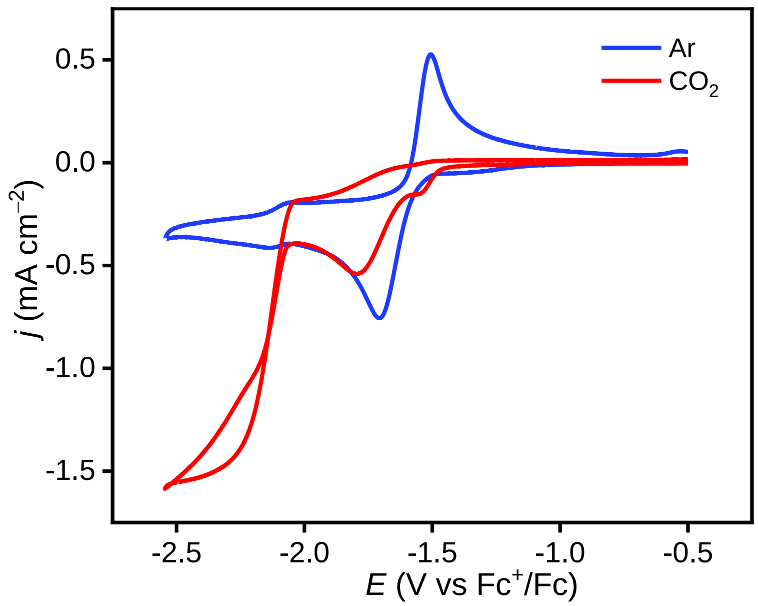


**Figure S4.** Cyclic voltammograms recorded on 1.5 mM **1c** at a GC electrode (diameter = 1 mm) using $\nu = 0.1$ V s$^{-1}$ in Ar- or $CO_2$-saturated 0.1 M $Bu_4NBF_4$/MeCN.

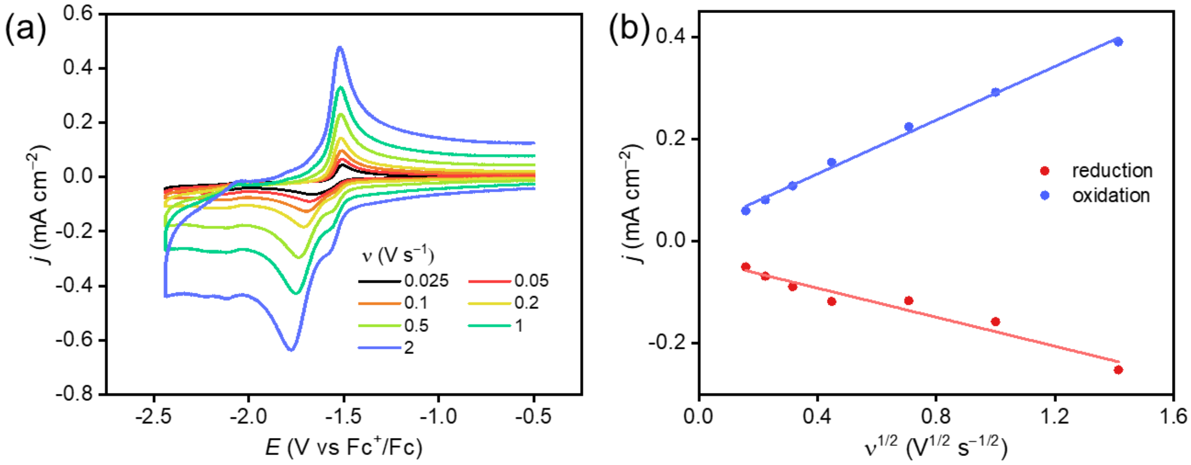


**Figure S5.** (a) Cyclic voltammograms recorded on ~0.5 mM **1a** at a GC electrode (diameter = 1 mm) using various sweep rates in Ar-saturated 0.1 M $Bu_4NBF_4$/MeCN. (b) Linear fitting the peak current densities vs square root of scan rates.

**Tables S3 and S4. Spin Population Analysis of 1a, 1a⁻, 4a, and 4a⁻**

The spin population change in the reduction of **1a** to **1a⁻** was analyzed to determine if the low $\pi^*$ orbital of the bpy-ligand contributes to its redox non-innocent character. It was found that in **1a⁻** the spin is located in the bpy, with a local density of 1.0 and a natural charge of –0.44 (Table S3), which is consistent with the delocalization of one unpaired electron over the ligand. Similar results were found for the reduction of the hydride species **4a** to **4a⁻** (Table S4).

**Table S3. Spin Population Analysis of Different Fragments in 1a and 1a⁻ to Determine the Location of the Electron upon Reduction (Hydrogens are Hidden in the 3D Models for Clarity).**

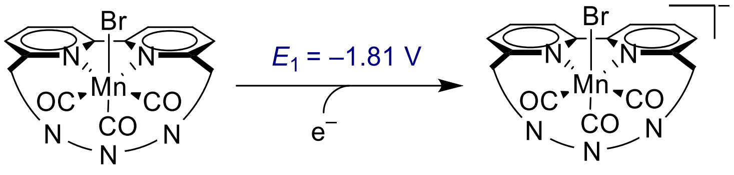


**1a** **1a⁻**

| | Fragment | Spin Density | | Natural Charge | |
|---|---|---|---|---|---|
| | | **1a** | **1a⁻** | **1a** | **1a⁻** |
| 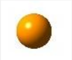 | **Mn** | 0.6 | 0 | –0.31 | –0.45 |
| 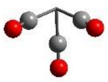 | **3CO** | 0 | 0.01 | 0.61 | 0.52 |
| 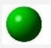 | **Bromide** | 0 | 0.02 | –0.53 | –0.57 |
| 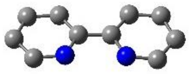 | **Bipyridine** | 0 | 1.0 | 0.35 | –0.44 |
| 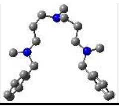 | **Other Fragments** | 0 | 0.04 | 0.51 | 0.40 |

**Table S4. Spin Population Analysis of Different Fragments in 4a and 4a⁻ to Determine the Location of the Electron upon Reduction (Hydrogens are Hidden in the 3D Models for Clarity).**

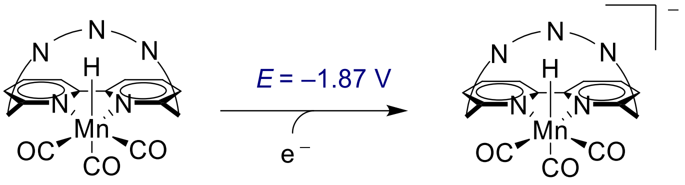


**4a** → **4a⁻**

| | Fragment | Spin Density | | Natural Charge | |
|---|---|---|---|---|---|
| | | **4a** | **4a⁻** | **4a** | **4a⁻** |
| 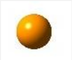 | **Mn** | 0 | 0 | –0.71 | –0.71 |
| 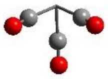 | **3CO** | 0 | 0.01 | 0.44 | 0.39 |
| 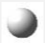 | **Hydride** | 0 | 0.02 | –0.15 | –0.18 |
| 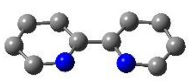 | **Bipyridine** | 0 | 0.98 | 0.32 | –0.50 |
| 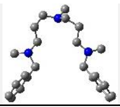 | **Other Fragments** | 0 | 0.02 | 0.08 | 0.001 |

**Figures S6-S13. Cyclic voltammograms and IR-SEC Figures**

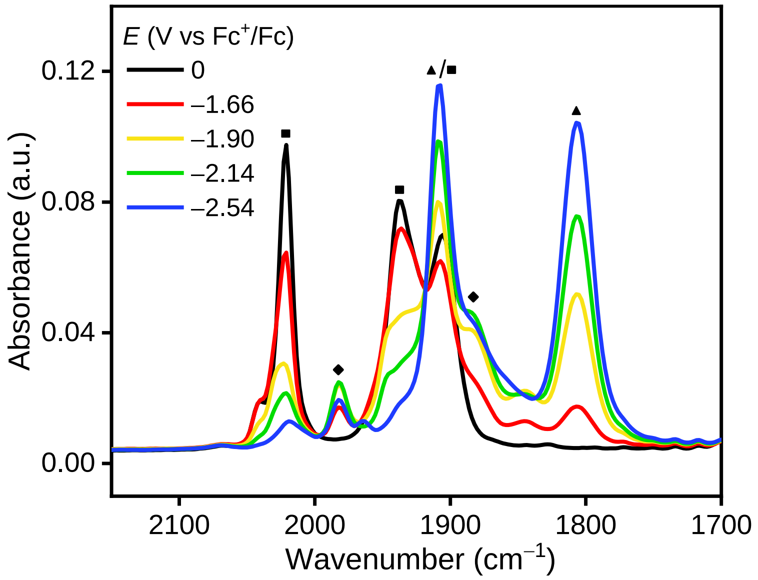


**Figure S6.** IR-SEC spectra ($\nu_{CO}$ region) recorded during voltammetric sweeping on 1.5 mM **1a** (using $v$ = 0.025 V s$^{-1}$) at specified potentials in the range from −2.54 to 0 V vs Fc$^+$/Fc in Ar-saturated 0.1 M $Bu_4NBF_4$/MeCN (■ = complex **1a**, ▲ = **2a**$^-$, ♦ = **4a**).

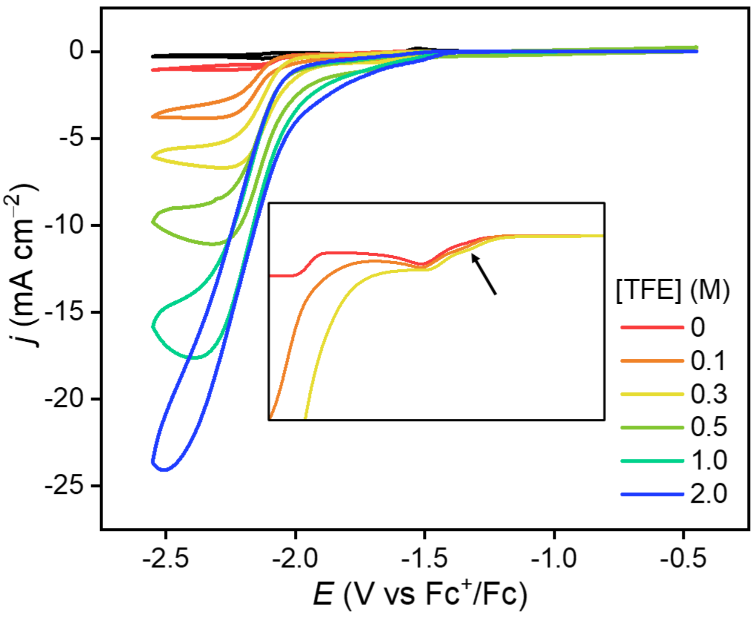


**Figure S7.** Cyclic voltammograms recorded on 1.5 mM **1a** at a GC electrode (diameter = 1 mm) using $\nu = 0.1$ V $s^{-1}$ in $CO_2$-saturated 0.1 M $Bu_4NBF_4$/MeCN with different [TFE]. A voltammogram recorded under Ar without TFE (black) is included as reference. Inset highlights the pre-wave assigned to the reduction of the solvent coordinated complex (**2a$^+$-MeCN**).[S36–S38]

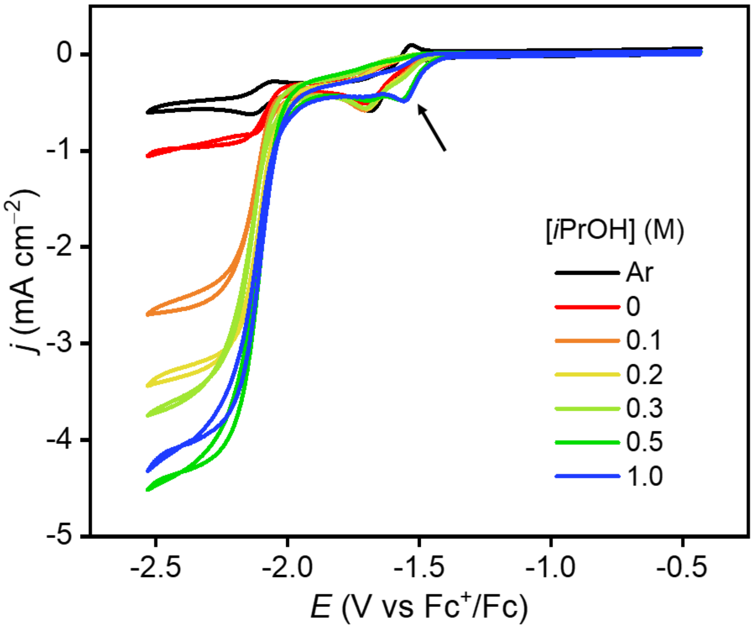


**Figure S8.** Cyclic voltammograms recorded on 1.5 mM **1a** at a GC electrode (diameter = 1 mm) using $\nu = 0.1$ V $s^{-1}$ in $CO_2$-saturated 0.1 M $Bu_4NBF_4$/MeCN with different [*i*PrOH]. A voltammogram recorded under Ar without *i*PrOH (black) is included as reference.

Pre-waves (marked by black arrows) at around –1.55 V vs $Fc^+$/Fc in Figures S7 and S8 are associated with reduction of the partially solvolyzed complex **2a$^+$-MeCN**.[S36–S38] This assignment agrees with DFT calculations, in which **2a$^+$-MeCN** shows a reduction potential of –1.36 V vs $Fc^+$/Fc (see Scheme 3).

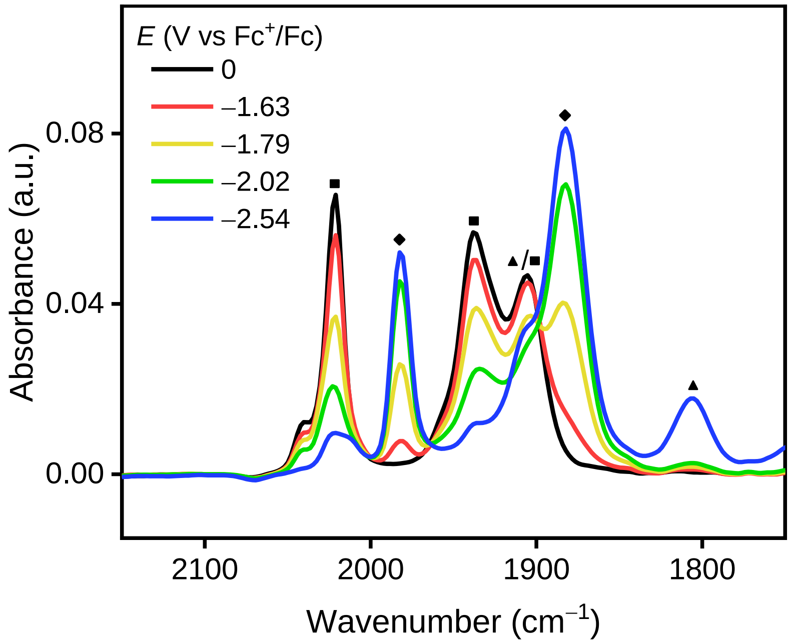


**Figure S9.** IR-SEC spectra ($\nu_{CO}$ region) recorded during voltammetric sweeping on 1.5 mM **1a** (using $v = 0.025$ V s$^{-1}$) at specified potentials in the range from –2.54 to 0 V vs Fc$^{+}$/Fc in 50% $CO_2$-saturated 0.1 M $Bu_4NBF_4$/MeCN (■ = complex **1a**, ▲ = **2a**$^{-}$, ♦ = **4a**).

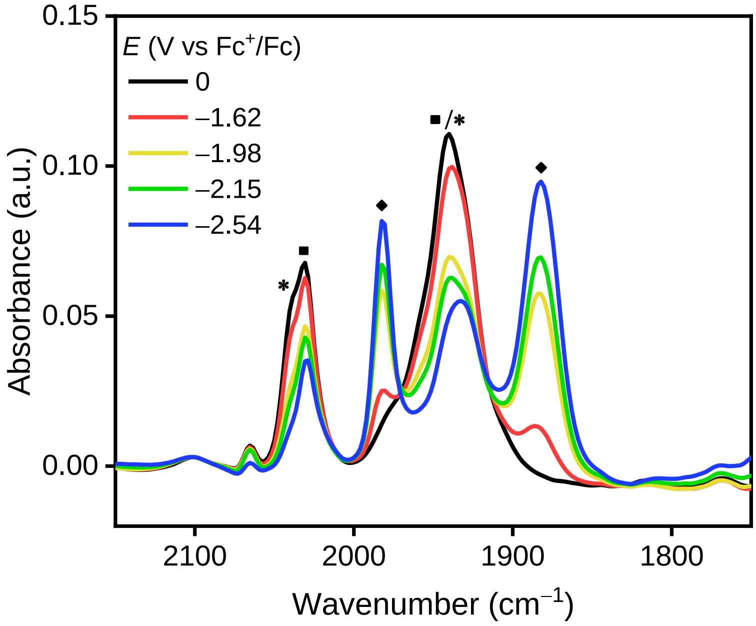


**Figure S10.** IR-SEC spectra ($\nu_{CO}$ region) recorded during voltammetric sweeping on a mixture of 1.5 mM **1a** and 2.0 M TFE (using $v = 0.025$ V s$^{-1}$) at specified potentials in the range from –2.54 to 0 V vs Fc$^{+}$/Fc in 50% $CO_2$-saturated 0.1 M $Bu_4NBF_4$/MeCN (■ = complex **1a**, ♦ = **4a**, ∗ = solvent-coordinated complex **2a**$^{+}$**-MeCN**).

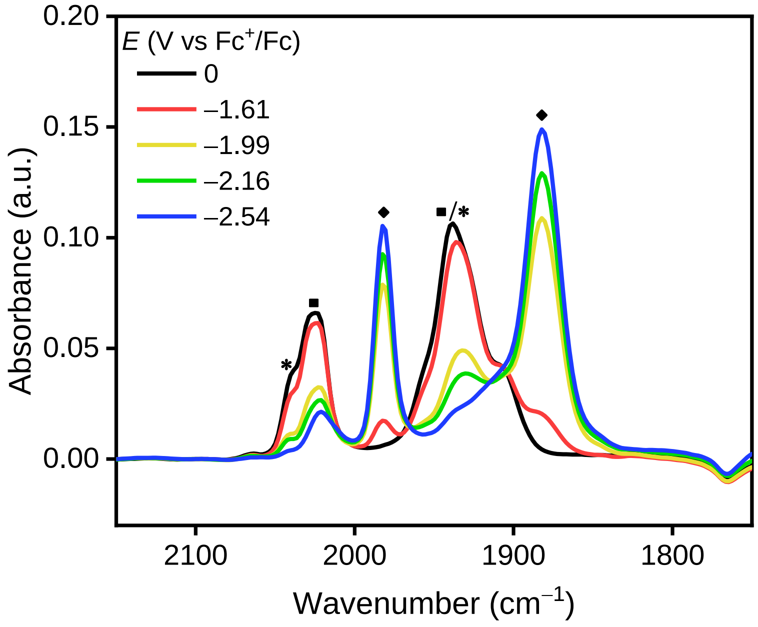


**Figure S11.** IR-SEC spectra ($\nu_{CO}$ region) recorded during voltammetric sweeping on a mixture of 1.5 mM **1a** and 1.0 M *i*PrOH (using $v = 0.025$ V s$^{-1}$) at specified potentials in the range from −2.54 to 0 V vs Fc$^+$/Fc in 50% $CO_2$-saturated 0.1 M $Bu_4NBF_4$/MeCN (■ = complex **1a**, ♦ = **4a,** ∗ = solvent-coordinated complex **2a**$^+$**-MeCN**).

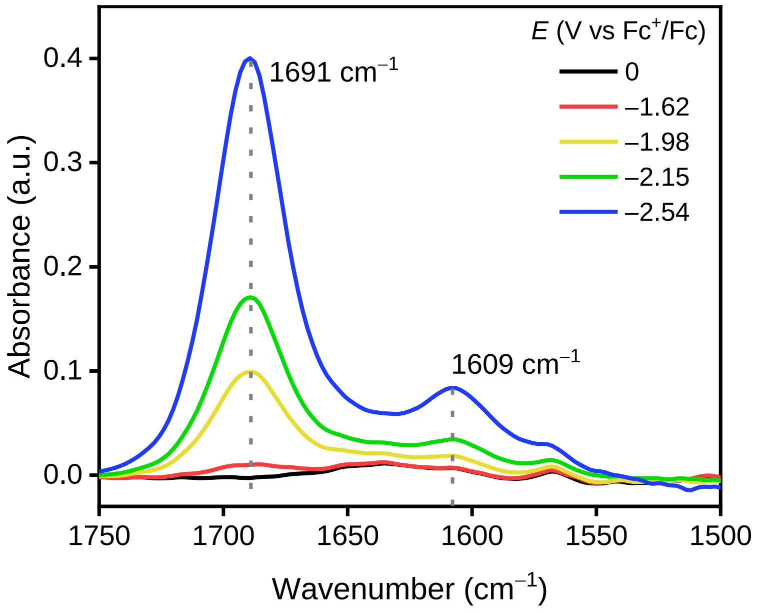


**Figure S12.** IR-SEC spectra ($\nu_{COO}$ region) recorded during voltammetric sweeping on a mixture of 1.5 mM **1a** and 2.0 M TFE (using $v = 0.025$ V s$^{-1}$) at specified potentials in the range from −2.54 to 0 V vs Fc$^+$/Fc in 50% $CO_2$-saturated 0.1 M $Bu_4NBF_4$/MeCN.

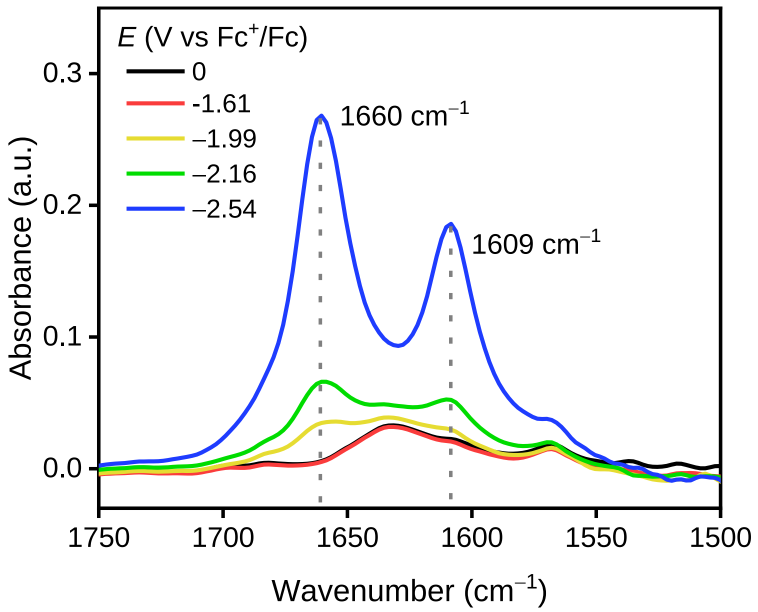


**Figure S13.** IR-SEC spectra ($\nu_{COO}$ region) recorded during voltammetric sweeping on a mixture of 1.5 mM **1a** and 1.0 M *i*PrOH (using $v$ = 0.025 V s$^{-1}$) at specified potentials in the range from −2.54 to 0 V vs Fc$^{+}$/Fc in 50% $CO_2$-saturated 0.1 M $Bu_4NBF_4$/MeCN.

**Table S5-S7. Controlled Potential Electrolysis Results**

**Table S5. Product Distributions Obtained after 1 h Electrolysis (at −2.25 V vs Fc$^{+}$/Fc) of 1.5 mM 1a–c in $CO_2$-saturated 0.2 M $Bu_4NBF_4$/MeCN Containing either 2.0 M TFE or 1.0 M *i*PrOH as Proton Source.**

| Complex | Proton donor | FE (%) | | |
|---|---|---|---|---|
| | | $H_2$ | HCOOH | CO |
| **1a** | 2.0 M TFE | 66 ± 5 | 11 ± 1 | 3 ± 0 |
| **1b** | 2.0 M TFE | 66 ± 3 | 3 ± 0 | 3 ± 0 |
| **1c** | 2.0 M TFE | 73 ± 1 | 3 ± 0 | 6 ± 1 |
| **1a** | 1.0 M *i*PrOH | 48 ± 3 | 29 ± 5 | 4 ± 0 |
| **1b** | 1.0 M *i*PrOH | 49 ± 1 | 28 ± 0 | 2 ± 0 |
| **1c** | 1.0 M *i*PrOH | 64 ± 4 | 14 ± 3 | 4 ± 1 |

**Table S6. $H_2$ Quantification Obtained after 1 h Electrolysis (at −2.25 V vs Fc$^{+}$/Fc) in the Absence or Presence of 1.5 mM 1a in $CO_2$-saturated 0.2 M $Bu_4NBF_4$/MeCN Containing 2.0 M TFE as Proton Source.**

| Entry | Catalyst | Atmosphere | Charge (C) | $H_2$ in the headspace (μmol) | $FE_{H2}$ (%) |
|---|---|---|---|---|---|
| 1 | --[a] | $CO_2$ | 1.3 ± 0.3 | 2.9 ± 0.3 | 47 ± 7 |
| 2 | **1a** | $CO_2$ | 16.6 ± 0.8 | 56.7 ± 0.3 | 66 ± 5 |

[a]no catalyst was present in this entry.

**Table S7. $H_2$ Quantification in Headspace after 1 h Electrolysis (at −2.25 V vs $Fc^+$/Fc) of 1.0 mM 1a* or 1a–c in Ar-saturated 0.2 M $Bu_4NBF_4$/MeCN Containing 2.0 M TFE as Proton Source.**

| Entry | Catalyst | Atmosphere | $H_2$ in headspace (μmol) | $FE_{H2}$ (%) |
|---|---|---|---|---|
| 1 | **1a*** | Ar | 132.5 ± 27.0 | 77 ± 13 |
| 2 | **1a** | Ar | 116.5 ± 3.5 | 73 ± 7 |
| 3 | **1b** | Ar | 141.5 ± 0.5 | 79 ± 3 |
| 4 | **1c** | Ar | 149.5 ± 2.5 | 85 ± 1 |

**Table S8. Benchmarking for Open System 1a***

The benchmarking was done for the open system **1a*** and the method most suitable for it was also applied for the closed system **1a** as it has the same metal, solvent, and similar ligand system. Three functionals were tested, B3LYP, TPSS, and TPSSh with two types of basis set combinations: def2SVP/def2-TZVP for Mn in combination with def2SVP/def2-TZVPD for rest of the atoms (denoted as **I** in Table S8) and def2SVP/def2-TZVP for Mn in combination with def2SVP/def2-TZVP for rest of the atoms (denoted as **II** in Table S8). The experimental redox potential of $E^0$ = −2.03V vs $Fc^+$/Fc for **4a*** reduction to **4a*⁻** was used for the benchmarking. Computational calculation of the redox potentials was done following the procedure shown in Section 3.3 (*vide infra*). The method showing the least error in experimental and calculated values was TPSSh with basis set combination **I**.

**Table S8. Benchmarking for the Open System 1a* with Different Functional and Basis Set Combinations to Find a Suitable Method for Static DFT Calculations.**

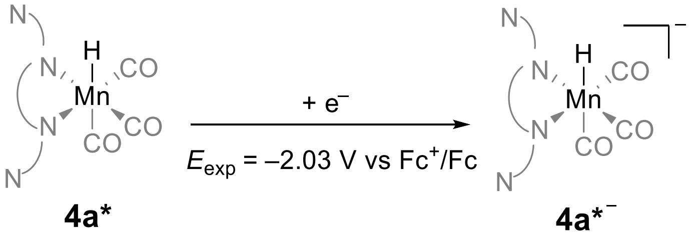


| Functional | Basis Set | $E^0$ (V vs $Fc^+$/Fc) Experimental | $E^0$ (V vs $Fc^+$/Fc) Calculated | Error (Exp-Cal) |
|---|---|---|---|---|
| B3LYP | I | −2.03 | −1.71 | −0.32 |
| | II | −2.03 | −2.32 | +0.29 |
| TPSS | I | −2.03 | −1.80 | −0.23 |
| | II | −2.03 | −1.81 | −0.22 |
| TPSSh | I | −2.03 | −1.84 | −0.19 |
| | II | −2.03 | −2.42 | +0.39 |

**Table S9. Table with Spin States of All Computed Stationary Points (X Refers to Middle or Side Amine).**

| Stationary Points | Charge | Spin Multiplicity |
|---|---|---|
| **1a, 1a*, 1b, 1c, 3a-N$_X$, 4a, 4a*, 5a, 5a*, 7a-N$_X$, 8a, 10a, 4a$_{ex}$, 5a$_{ex}$, 8a$_{ex}$, 10a$_{ex}$, TS-(2-7, 9, 17-20, 22, 28, 29, 31, 32, 37, 38)** | 0 | 1 |
| **2a$^–$, 2a*$^–$, 11a$_{ex}^–$, 11a$^–$, 11a*$^–$, TS-(1, 1', 16, 26, 27, 30, 33, 36)** | –1 | 1 |
| **2a, 6a-N$_X$, 9a-N$_X$, 12a, 13a, TS-(15, 25, 35)** | 0 | 2 |
| **1a$^–$, 4a$^–$,4a$_{ex}^–$, 5a$^–$, 5a$_{ex}^–$, 8a$^–$, 8a$_{ex}^–$, 10a$^–$, 10a$_{ex}^–$, TS-(11-14, 24, 34)** | –1 | 2 |
| **2a$^+$, 2a$^+$-MeCN, 6a$^+$-N$_X$, 6a*$^+$, 9a$^+$-N$_X$ , 12a$^+$, 12a$_{ex}^+$, 13a$^+$, 13a$_{ex}^+$, TS-(8, 10, 21, 23)** | 1 | 1 |

## Scheme S2-S9. DFT Calculations for the Energy Profile Diagrams

## Scheme S2. Computational Protocol Followed for AIMD Simulations and Static DFT Calculations.

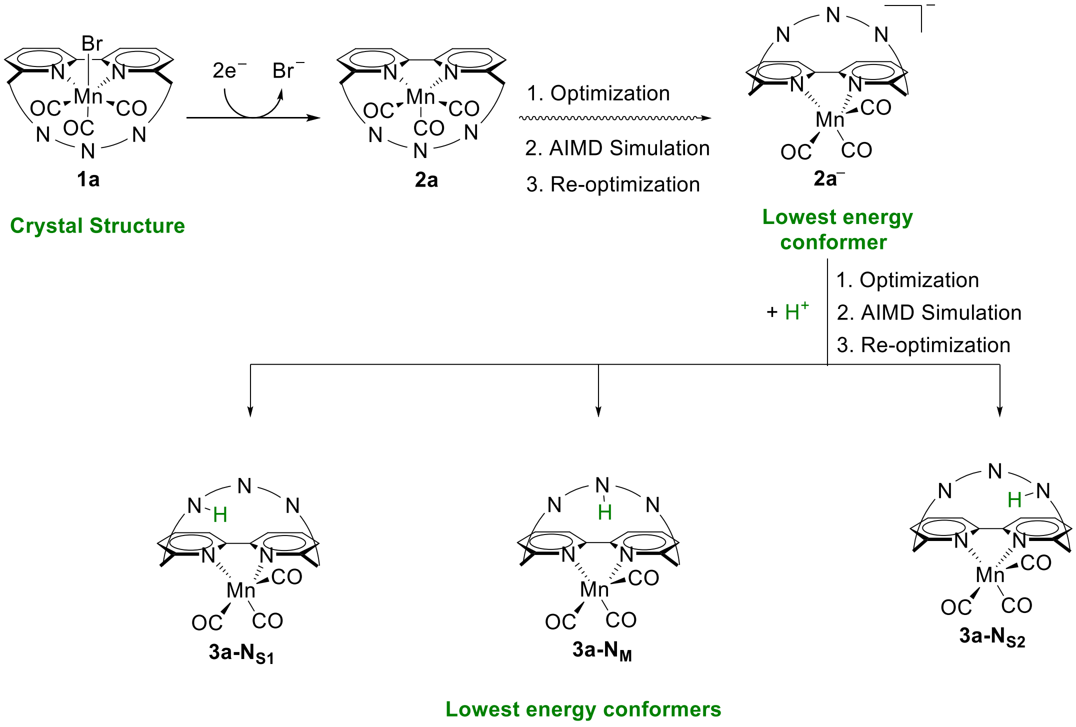

**Scheme S3. DFT-calculated Energy Profile for the Reduction of $CO_2$ to Three Different Products, i.e. $H_2$ (green), $HCOO^-$ (red), and CO (blue) Using the *endo* Conformation[a] and Side ($N_S$)[b] Amine Moiety as Proton Shuttle.[c]**

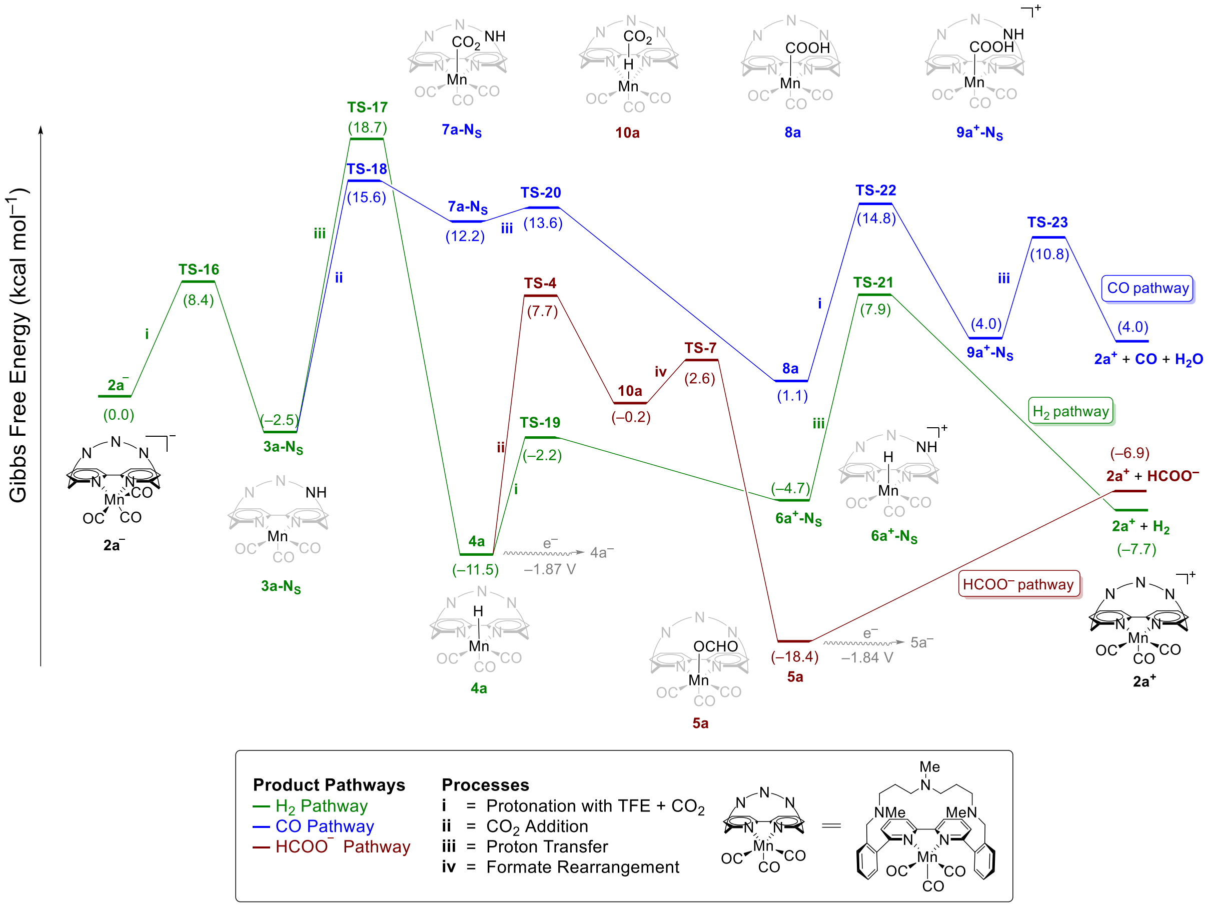


[a]*endo* profile refers to the pentacoordinate complex with ligand in the same direction as the metal vacant site.

[b]The subscript on N is used to denote the side ($N_S$) and middle ($N_M$) amine moieties. Redox potentials are referenced against $Fc^+$/Fc couple.

[c]All energies are relative to a redox potential of –1.60 V vs $Fc^+$/Fc.

**Scheme S4. DFT-calculated Energy Profile Starting from the Reduced Mn-Hydride 4a⁻ to Form $H_2$ (green) and $HCOO^-$ (red) Using the *endo* Conformation and the Side ($N_S$) Amine Moiety as Proton Shuttle.**[a]

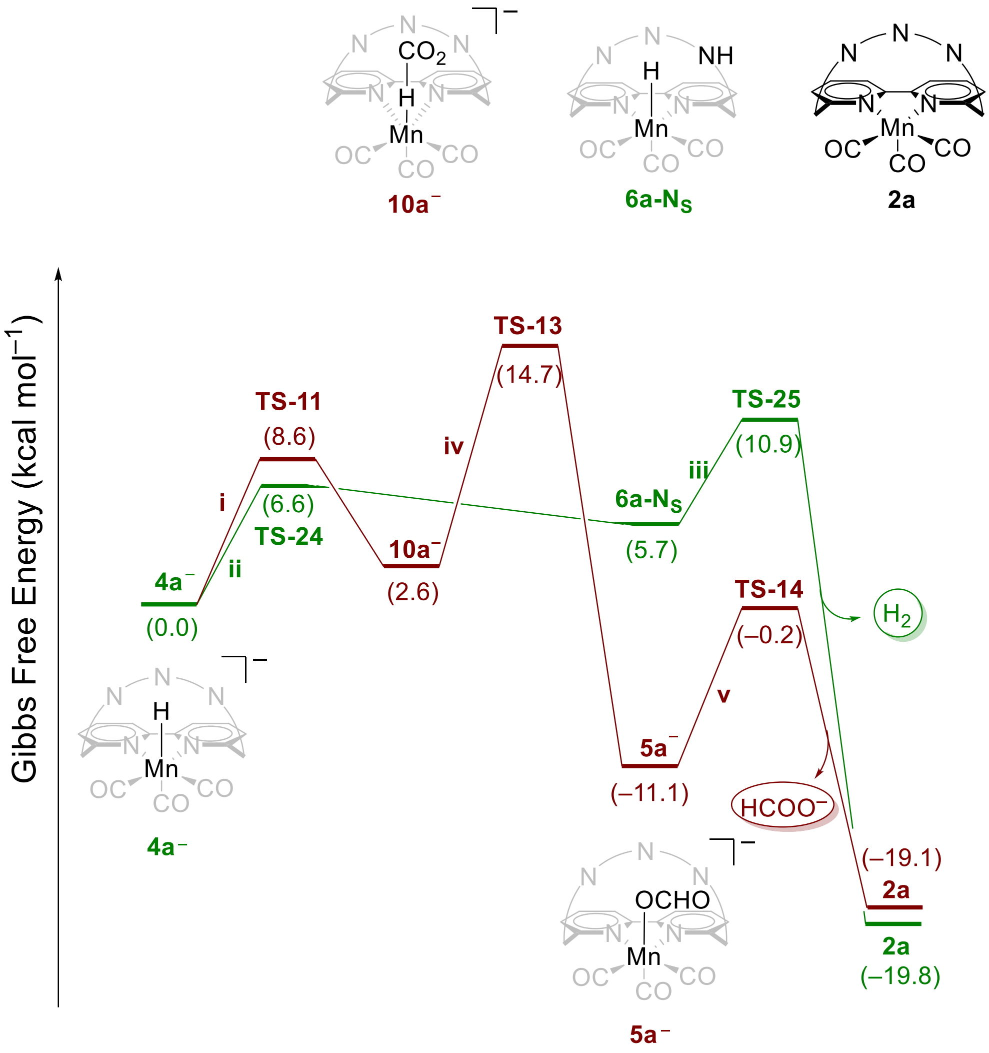


| **Product Pathways** | **Processes** |
|---|---|
| — $H_2$ Pathway | **i** = $CO_2$ Addition |
| — $HCOO^-$ Pathway | **ii** = Protonation with TFE + $CO_2$ |
| | **iii** = Proton Transfer |
| | **iv** = Formate Rearrangement |
| | **v** = Bond Elongation |

[a]All energies are relative to the calculated redox potential of –1.87 V vs $Fc^+/Fc$.

**Scheme S5. DFT-calculated Energy Profile for the Reduction of $CO_2$ to Three Different Products, i.e. $H_2$ (green), $HCOO^-$ (red), and CO (blue) Using the *exo* Conformation.[a, b]**

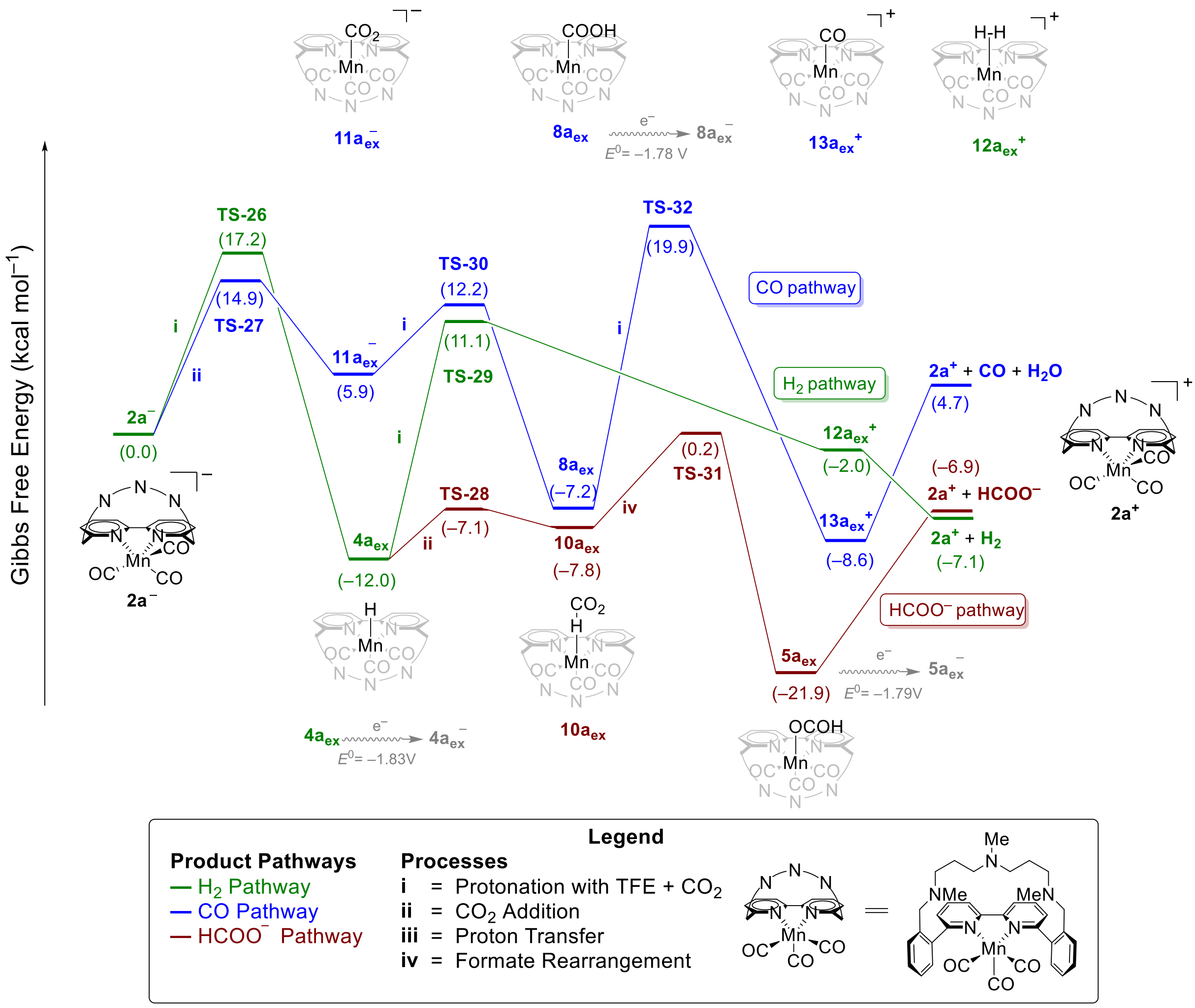


[a]*exo* profile refers to the pentacoordinate complex with ligand in the opposite direction of the metal vacant site. [b]All energies are relative to a redox potential of –1.60 V vs $Fc^+$/Fc.

**Scheme S6. DFT-calculated Energy Profile for CO Formation through Direct $CO_2$ Addition to the Metal Center (grey) and $N_M$ Assisted $CO_2$ Addition (blue) Considered before and after the Reduction of 8a to 8a⁻.[a]**

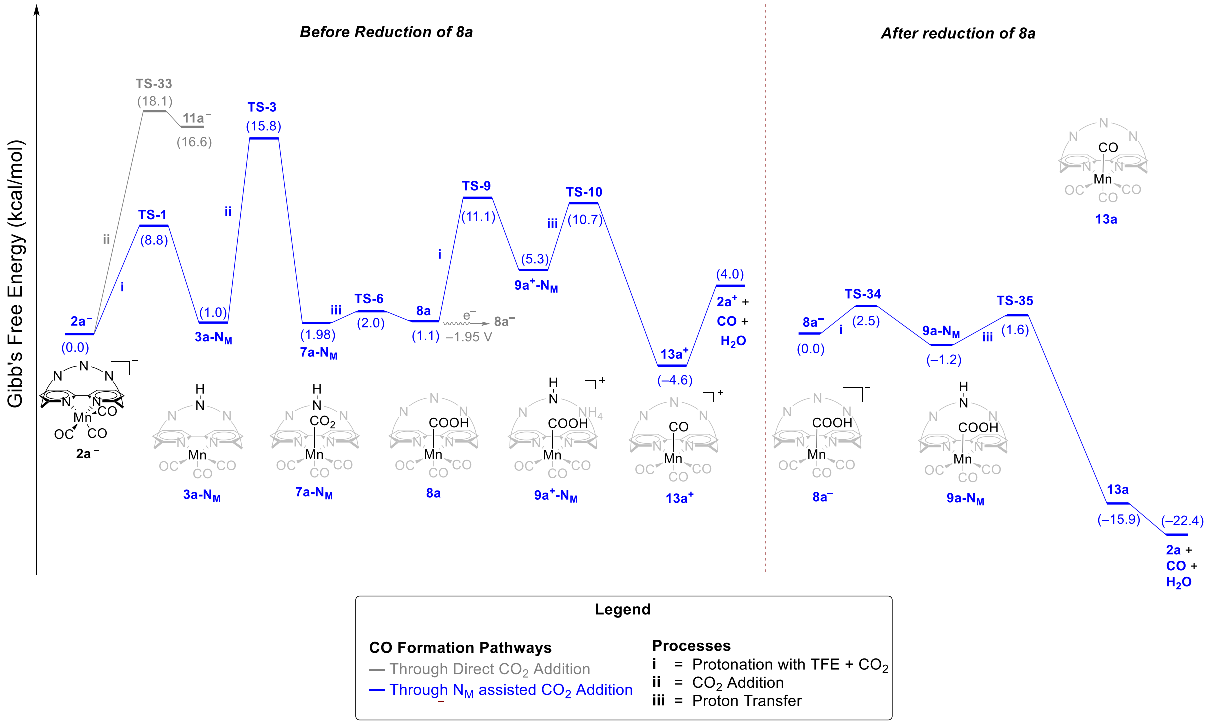


[a]All energies before reduction of **8a** are relative to a redox potential of –1.60 V vs Fc⁺/Fc. After reduction of **8a**, they are considered at a calculated redox potential of –1.95 V vc Fc⁺/Fc.

**Scheme S7. DFT-calculated Energy Barriers of (a) Closed 1a and (b) Open 1a* Complex for the Key Steps in $H_2$ (green), $HCOO^-$ (red), and CO (blue) Formations.**

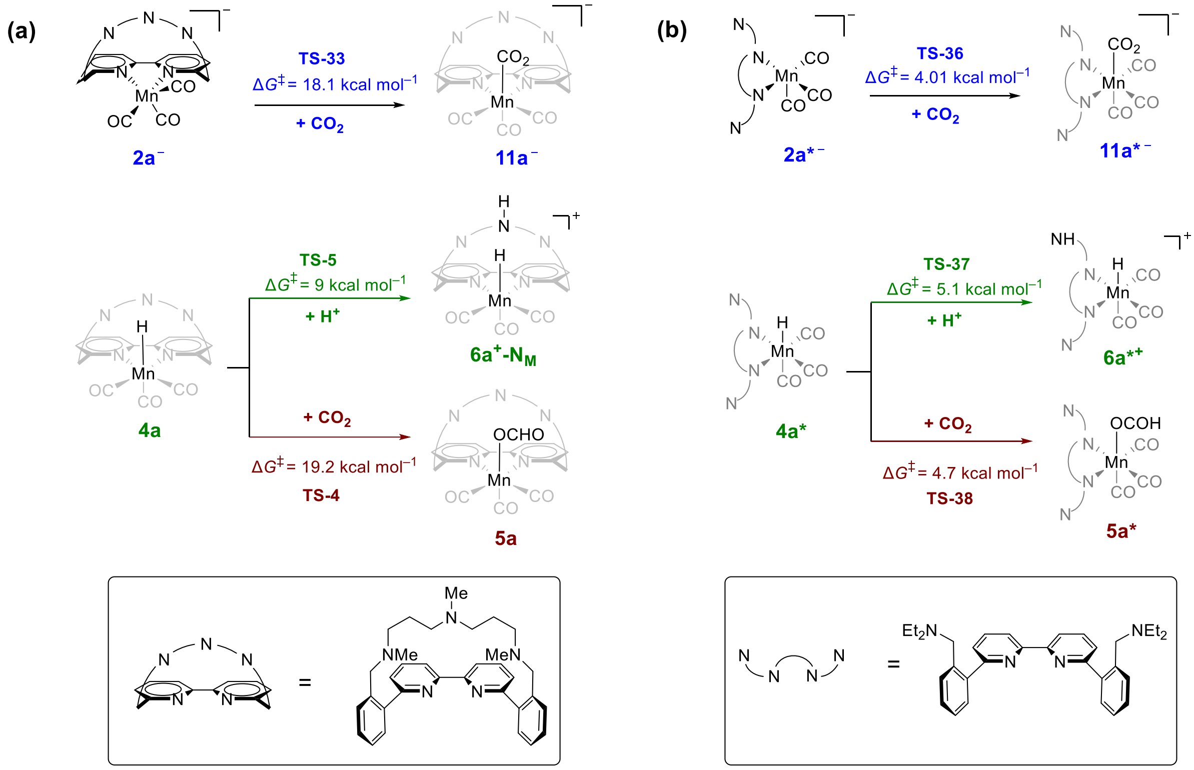

**Scheme S8. DFT-calculated Thermochemistry of Protonation with $CO_2$ + TFE, $H_2O$ + $CO_2$, and $H_2O$ as Proton Sources.[a] Protonation Steps Take Place from (a) $2a^-$ Followed by Proton Transfer to the Metal, (b) 4a, and (c) 8a.**

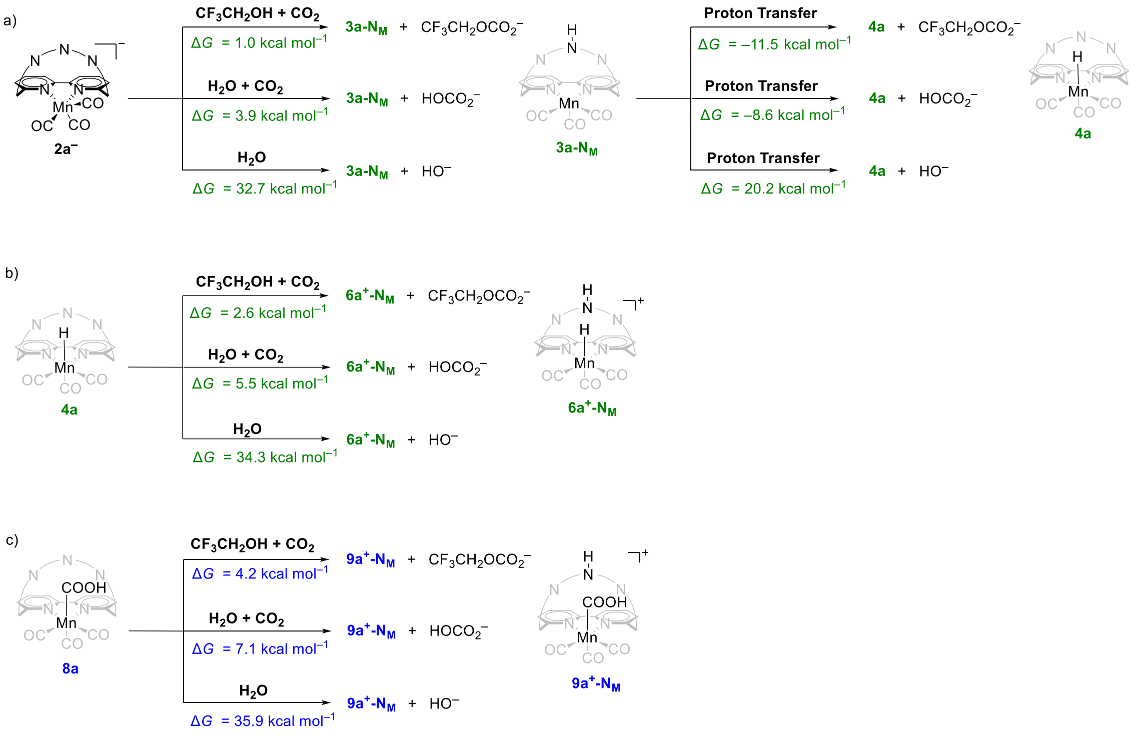


[a]Energies in a), b) and c) are calculated by taking **$2a^-$**, **4a**, and **8a** as the zero of energy, respectively.

**Scheme S9. DFT-calculated Energy Profile for the Reduction of $CO_2$ to Three Different Products: $H_2$ (green), $HCOO^-$ (red), and CO (blue) Using the *endo* Conformation[a] and Middle ($N_M$)[b] Amine Moiety as a Proton Shuttle[c] Including All Intermediates.** [b, c, d, e]

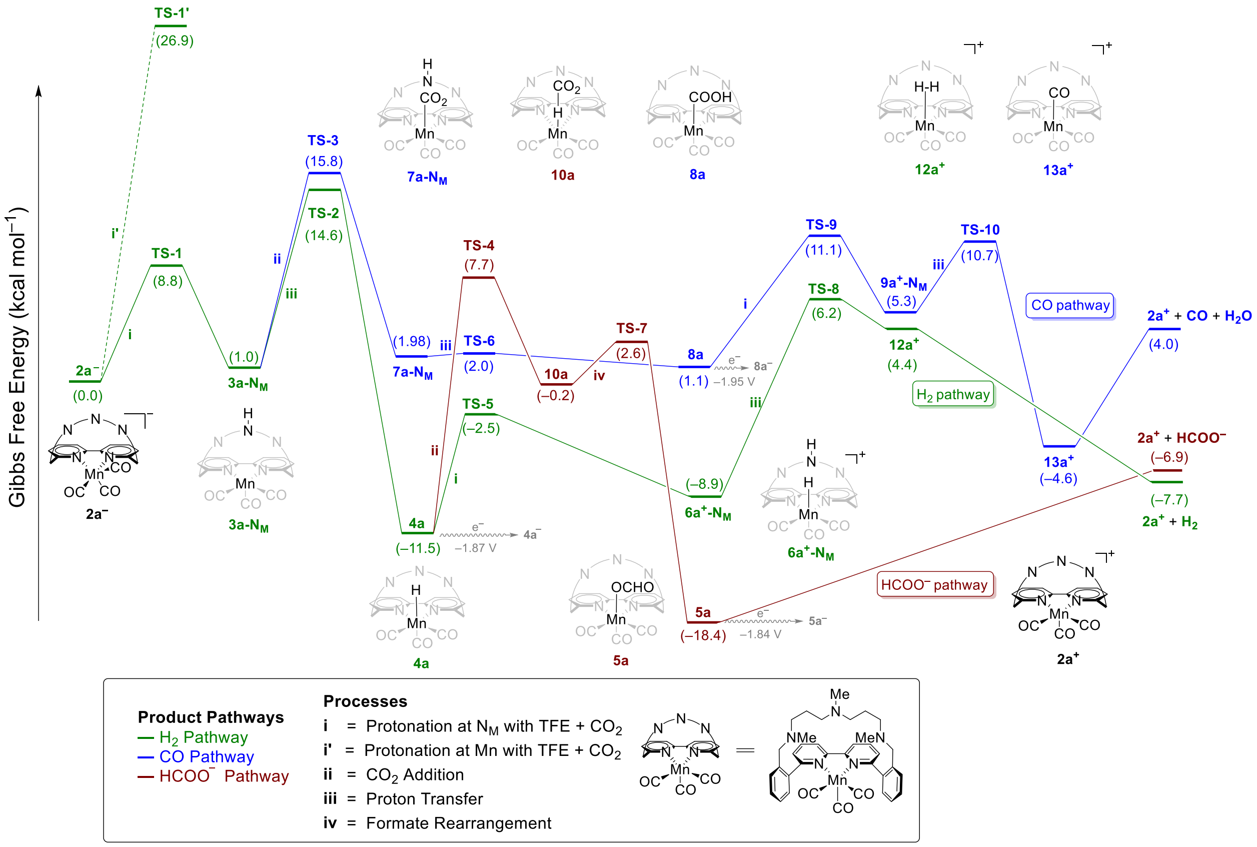


[a]*Endo* profile refers to the pentacoordinate complex with ligand in the same direction as the metal vacant site.

[b]All energies are relative to the redox potential of –1.60 V vs $Fc^+/Fc$.

[c]Wiggly arrows show the calculated reduction potentials for **4a**, **5a**, and **8a**. Energy profiles after reduction are displayed in Scheme 5 (at –1.87 V vs $Fc^+/Fc$) and Scheme S6 (at –1.95 V vs $Fc^+/Fc$).

[d]Numbers in energy profile indicate relative Gibbs free energy in kcal $mol^{-1}$.

## 5. Trace Crossing (Figures 4, S3, and S4)

In Figures 4, S3, and S4 (red curves), trace crossing appears with an increase in the catalytic current response on the reverse scan in the voltammograms for catalysts **1a–c** under $CO_2$ atmosphere. The fact that trace crossing appears in the catalytic region indicates an acceleration of the catalytic behavior. As discussed in the main text (see also Scheme 1), **2**$^{-}$ is generated because of a 2-electron reduction reaction of **1** at around –1.69 V vs $Fc^+$/Fc, after which a proton can be shuttled from residual water to the metal center via the amine-bearing ligand. This generates the manganese hydride (**4**), which after further reduction enters the catalytic cycle, resulting in the increasing catalytic current. To explain trace crossing, we hypothesize that as the potential is shifted toward more negative values, a further ligand-based reduction of **2**$^{-}$ to **2**$^{2-}$ can take place at –2.3 V vs $Fc^+$/Fc. In fact, the reduction of the bpy-ligand is reported to occur at this potential.[S39] The basicity of **2**$^{2-}$ can be assumed to be higher than that of **2**$^{-}$, implying that the former will become protonated relatively faster by $CO_2$/$H_2O$. This will lead to the rapid formation of reduced manganese hydride (**4**$^{-}$), which as a strongly activated electrocatalyst causes the build-up of current on the reverse scan. Recently, a similar explanation was proposed by Wu et al. for a manganese terpyridine complex used for HER.[S40]

To exclude catalyst adsorption on the electrode surface as the cause of trace crossing, the electrode used for the voltametric experiments under $CO_2$ was transferred from the original catalyst-containing solution to another electrochemical cell containing only $CO_2$-saturated 0.1 M $Bu_4NBF_4$/MeCN (Figure S14). No catalytic response was observed in the voltammogram (green line) as opposed to the one recorded in the original solution (red line), demonstrating that no complex would be adsorbed on the electrode surface.

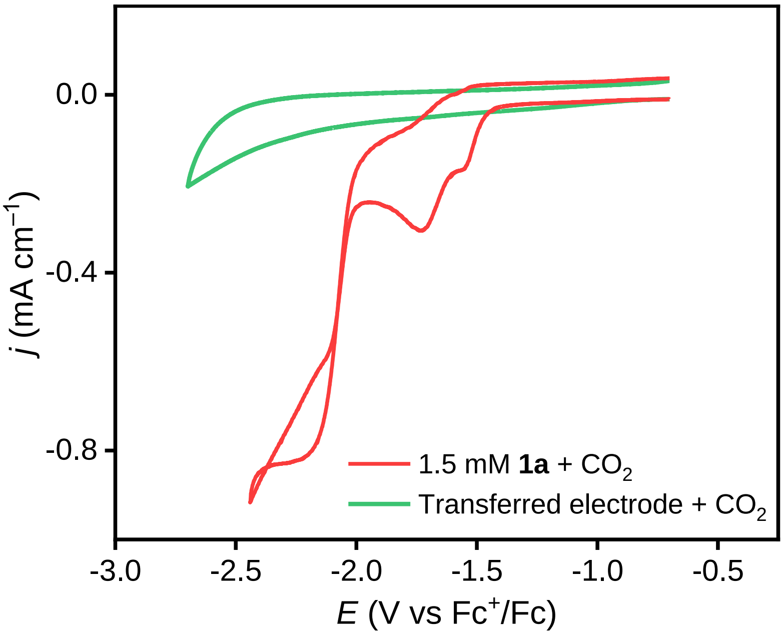


**Figure S14.** Cyclic voltammogram recorded on 1.5 mM **1a** at a GC electrode (diameter = 1 mm) using $v$ = 0.1 V $s^{-1}$ in $CO_2$-saturated 0.1 M $Bu_4NBF_4$/MeCN (red) together with a corresponding cyclic voltammogram recorded at the same electrode after being transferred to $CO_2$-saturated 0.1 M $Bu_4NBF_4$/MeCN (green).

## 6. Residual Water in Electrochemical System

The concentration of residual water and its effect on cyclic voltammograms (Figures 4, S3, and S4) and controlled potential electrolysis are addressed in this section.

### 6.1 Quantification of Residual Water in Electrochemical Cell

Karl-Fischer Titration was applied to measure the water content of various MeCN solutions. The water content of MeCN itself, collected from an MBRAUN MB SP-800 purification system, was measured to be 30–50 ppm. With 0.1 M $Bu_4NBF_4$ added, it increased to 300–400 ppm. After purging the solution with Ar or $CO_2$ for 15–20 min, it increased further to 650–750 ppm, indicating that a considerable amount of water is introduced by both the supporting electrolyte and the purging gas. Note that a water content of 750 ppm equals a concentration of 0.034 M, which is significant but, nevertheless, low compared with the high concentrations employed of the external added proton sources, i.e. TFE (2.0 M) and *i*PrOH (1.0 M).

Based on cyclic voltammograms, the catalytic effect can be expressed by $i_{cat}/i_p$, where $i_{cat}$ is the catalytic plateau current and $i_p$ is the peak current under Ar. In Figure 4, the ratio of $i_{cat}/i_p$ equals 2.5 for **1a**, with residual water acting as proton source. Notably, in the presence of an externally added proton donor (2.0 M TFE), this ratio increases to as much as 61.5 (Figure S7), showing the tremendous impact of having both the stronger proton donor and the amine functional groups in the secondary sphere if an excellent catalytic efficiency is to be achieved.

### 6.2 Cyclic Voltammograms Recorded in Dried Electrolyte

To further prove that the catalytic current in the absence of added proton sources was originating from residual water, cyclic voltammograms were recorded for **1a** after adding freshly activated 3Å molecular sieves to the $CO_2$-saturated electrolyte (15–20 min prior to experiment). This resulted in a lowering of the water content to ~90 ppm (~0.004 M). Figure S15 shows cyclic voltammograms recorded on 1 mM **1a** under such drier conditions under both $CO_2$ and Ar. In contrast to the situation before (Figure 4), an oxidation wave of **2a**$^-$ starts to appear on the reverse scan under $CO_2$. At the same time, the reduction wave of **4a** at –2.1 V vs $Fc^+/Fc$ is small. These results support that higher stability of **2a**$^-$ can be achieved under drier conditions, while inhibiting the formation of **4a**, although it is difficult to completely avoid interfering effects of residual water in the absence of externally added proton donors.

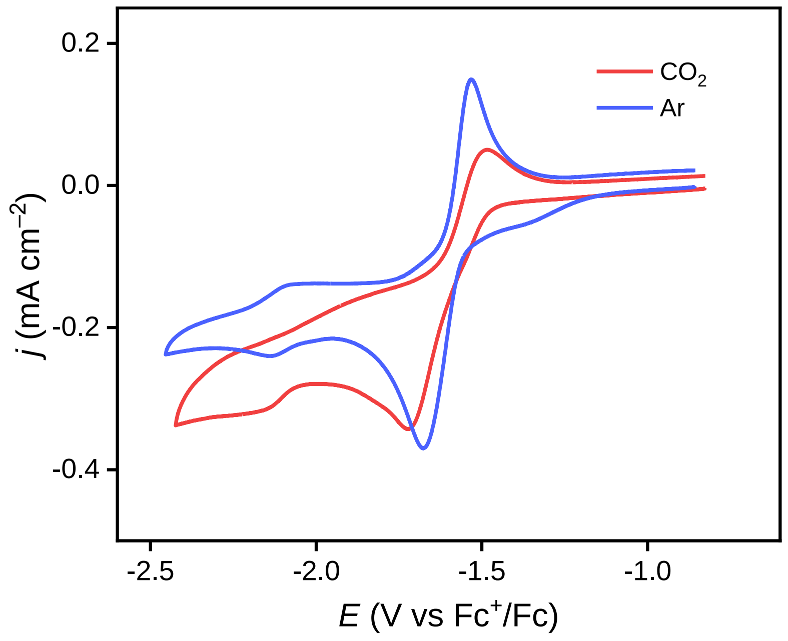


**Figure S15**. Cyclic voltammograms recorded on 1.0 mM **1a** at a GC electrode (diameter = 1 mm) using $\nu = 0.1\ \mathrm{V\ s^{-1}}$ in Ar- (blue) or $CO_2$-saturated (red) 0.1 M $Bu_4NBF_4$/MeCN, after adding freshly activated 3Å molecular sieves as drying agent to the electrolyte solution (15–20 min prior to experiment).

## 6.3 Control Electrolysis with Residual Water as Proton Source under $CO_2$

To uncover the influence of residual water on HER (see Figure 5), electrolysis was conducted in $CO_2$-saturated 0.2 M $Bu_4NBF_4$/MeCN. The charge consumed during 1 h electrolysis of **1a** was 1.0 C (Figure S16), with a yield of $H_2$ in the headspace of less than 1 μmol. In comparison, the charge consumption increased to 17.5 C if the electrolysis of **1a** was carried out in a $CO_2$-saturated 0.2 M $Bu_4NBF_4$/MeCN containing 2.0 M TFE. At the same time, the overall yield of products increased by two orders of magnitude. This significant difference corroborates the negligible influence residual water exerts once a high concentration of external proton donor is applied. The other manganese catalysts are expected to behave in the same manner.

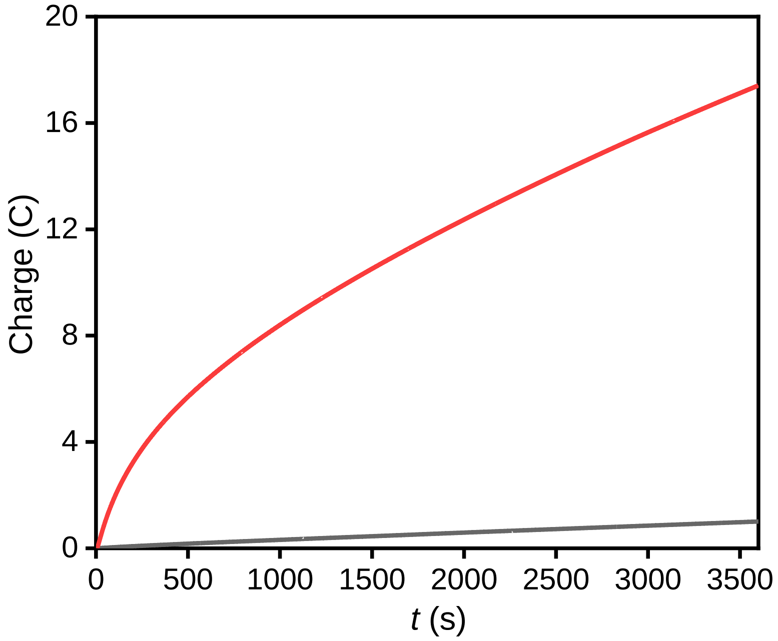


**Figure S16.** Consumed charge during 1 h electrolysis (at –2.25 V vs $Fc^+/Fc$) of 1.5 mM **1a** in $CO_2$-saturated 0.2 M $Bu_4NBF_4$/MeCN (grey) or $CO_2$ -saturated 0.2 M $Bu_4NBF_4$/MeCN containing 2.0 M TFE as proton source (red).

Based on these results, it is important to emphasize that although the concentration of residual water is appreciable (~0.034 M), its influence on our evaluation of the electrochemical performance of **1a–c** would be negligible once proton donors such as 2.0 M TFE or 1.0 *i*PrOH are applied.

# 7. Synthesis of Relevant Materials

## 7.1 Synthesis of Manganese Complexes

**Mn(6,10,14-trimethyl-6,10,14-triaza-2,3(2,6)-dipyridina-1,4(1,2) dibenzenacyclopentadecaphane)(CO)$_3$Br (1a)**

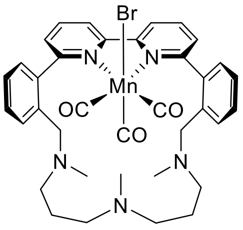


**1a**

Ligand **L1** (101 mg, 0.2 mmol, 1.0 equiv.) and Mn(CO)$_5$Br (55 mg, 0.2 mmol, 1.0 equiv.) were dissolved in THF (1.0 mL) in an Ar-filled glovebox. The solution was heated to 55 ºC and stirred under Ar, shielded from light. The suspension was filtered, and the solid was washed with pentane (5 × 1 mL) and Et$_2$O (3 × 1 mL) to give the product as a yellow powder (126 mg, 87 % yield).

**$^1$H NMR** (400 MHz, CD$_2$Cl$_2$) ($\delta$) ppm: 8.19 (d, $J$ = 8.1 Hz, 2H), 7.96 (t, $J$ = 7.9 Hz, 2H), 7.62 – 7.55 (m, 2H), 7.46 – 7.38 (m, 6H), 7.35 – 7.29 (m, 2H), 4.00 (d, $J$ = 12.8 Hz, 2H), 3.03 (d, $J$ = 12.8 Hz, 2H), 2.21 – 1.80 (m, 18H), 0.97 (bs, 3H). **$^{13}$C NMR** (101 MHz, (CD$_3$)$_2$SO) ($\delta$) ppm: 164.1 (2C), 156.6 (2C), 141.8 (2C), 138.0 (2C), 136.7 (2C), 130.99 (2C), 130.96 (2C), 129.4 (2C), 128.6 (2C), 127.6 (2C), 122.0 (2C), 60.5 (2C), 56.9 (2C), 54.9 (2C), 41.9, 41.3 (2C), 24.9 (2C). **IR** (ATR, cm$^{-1}$) $\tilde{\nu}$ = 2010 (s), 1919 (s), 1900 (s), 1458 (w), 796 (m), 766 (m), 753 (m), 734 (w), 629 (m), 530 (w). **HRMS** (ESI$^+$) m/z calcd. for $C_{36}H_{40}BrMnN_5O_3$ [M+H]$^+$: 724.1690 (100%), 726.1670 (97%); found: 724.1683, 726.1675; m(sigma) = 23.9.

**Mn(6,14-dimethyl-10-oxa-6,14-diaza-2,3(2,6)-dipyridina-1,4(1,2)-dibenzenacyclopentadecaphane)(CO)$_3$Br (1b)**

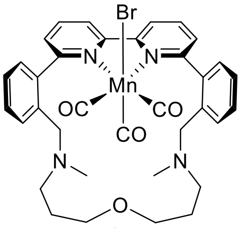


**1b**

Ligand **L2** (75 mg, 0.15 mmol, 1.0 equiv.) and Mn(CO)$_5$Br (50 mg, 0.18 mmol, 1.2 equiv.) were dissolved in THF (1.0 mL) in an amber reaction tube in an Ar-filled glovebox. The solution was heated to 55 ºC and stirred for 6 h. After this time, the reaction mixture was placed at –35 °C overnight. The solution was concentrated to approx. 0.5 mL and left in a large vial with pentane to crash out the complex by vapor diffusion. The mixture was filtered, and the solid was washed with pentane (3 × 1 mL) and Et$_2$O (3 × 1 mL) to give the product as a yellow powder (50 mg, 46 % yield).

**$^1$H NMR** (400 MHz, CD$_2$Cl$_2$) ($\delta$) ppm: 8.19 (dd, $J$ = 8.1, 1.3 Hz, 2H), 7.94 (t, $J$ = 7.9 Hz, 2H), 7.62 – 7.57 (m, 2H), 7.46 – 7.37 (m, 6H), 7.34 – 7.28 (m, 2H), 4.00 (d, $J$ = 13.0 Hz, 2H), 3.18 (d, $J$ = 13.1 Hz, 2H), 2.92 (t, $J$ = 6.2 Hz, 4H), 2.25 (ddd, $J$ = 12.3, 8.4, 5.5 Hz, 2H), 2.02 (ddd, $J$ = 12.3, 8.1, 6.4 Hz, 2H), 1.85 (s, 6H), 1.33 – 1.19 (m, 2H), 1.03 (dp, $J$ = 13.4, 6.4 Hz, 2H). **$^{13}$C NMR** (101 MHz, CD$_2$Cl$_2$) ($\delta$) ppm: 166.2 (2C), 157.4 (2C), 142.5 (2C), 137.8 (2C), 137.4 (2C), 132.1 (2C), 131.3 (2C), 130.0 (2C), 129.1 (2C), 128.2 (2C), 121.5 (2C), 68.7 (2C), 62.4 (2C), 56.4 (2C), 41.3 (2C), 28.3 (2C). **IR** (ATR, cm$^{-1}$) $\tilde{\nu}$ = 2009 (s), 1920 (s), 1893 (s), 1116 (w), 1028 (w), 795 (m), 765 (m), 751 (m), 630 (m), 529 (w). **HRMS** (ESI$^+$) m/z calcd. for $C_{35}H_{37}BrMnN_4O_4$ [M+H]$^+$: 711.1373 (100.0%), 713.1353 (97%); found: 711.1373, 713.1370; m(sigma) = 14.4.

**Mn(6,13-dimethyl-6,13-diaza-2,3(2,6)-dipyridina-1,4(1,2) dibenzenacyclotetradecaphane)(CO)$_3$Br (1c)**

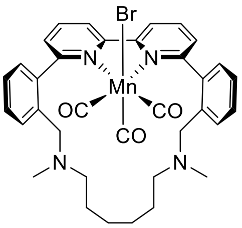


**1c**

Ligand **L3** (60 mg, 0.13 mmol, 1.0 equiv.) and Mn(CO)$_5$Br (35 mg, 0.13 mmol, 1.0 equiv.) were dissolved in THF (0.6 mL) in an amber reaction tube in an Ar-filled glovebox. The solution was heated to 55 ºC and stirred for 6 h under Ar, shielded from light. The suspension was filtered, and the solid was washed with pentane (5 × 1 mL) to give the product as a dark yellow powder (68 mg, 77 % yield).

**$^{1}$H NMR** (400 MHz, $CD_2Cl_2$) ($\delta$) ppm: 8.20 (dd, $J$ = 8.1, 1.3 Hz, 2H), 7.98 (t, $J$ = 7.9 Hz, 2H), 7.56 – 7.31 (m, 10H), 4.17 (d, $J$ = 12.6 Hz, 2H), 2.72 (d, $J$ = 12.5 Hz, 2H), 2.06 (td, $J$ = 11.9, 5.9 Hz, 2H), 1.94 – 1.84 (m, 8H), 1.10 – 0.98 (m, 2H), 0.97 – 0.81 (m, 2H), 0.75 – 0.62 (m, 2H), 0.47 – 0.33 (m, 2H). **$^{13}$C NMR** (101 MHz, $CD_2Cl_2$) ($\delta$) ppm: 165.8 (2C), 157.7 (2C), 142.3 (2C), 138.0 (2C), 137.8 (2C), 132.0 (2C), 131.7 (2C), 129.9 (2C), 129.3 (2C), 128.3 (2C), 121.3 (2C), 60.1 (2C), 58.8 (2C), 43.4 (2C), 25.5 (2C), 25.1 (2C). **IR** (ATR, $cm^{-1}$) $\tilde{\nu}$ = 2012 (s), 1922 (s), 1906 (s), 799 (m), 766 (m), 754 (m), 736 (w), 679 (w), 628 (m), 530 (w). **HRMS** ($ESI^{+}$) m/z calcd. for $C_{35}H_{37}BrMnN_4O_3$ $[M+H]^{+}$: 695.1425 (100%), 697.1404 (97%); found: 695.1429, 697.1416; m(sigma) = 24.8.

## $^{13}$C-NMR Characterization of 1a

Due to difficulties obtaining a high intensity $^{13}$C-NMR spectrum in $CD_2Cl_2$ and DMSO-$d_6$, the carbon atoms of complex **1a** have been assigned by comparison of a $^{13}$C-NMR spectrum and an Attached Proton Test (APT) NMR experiment as shown below.

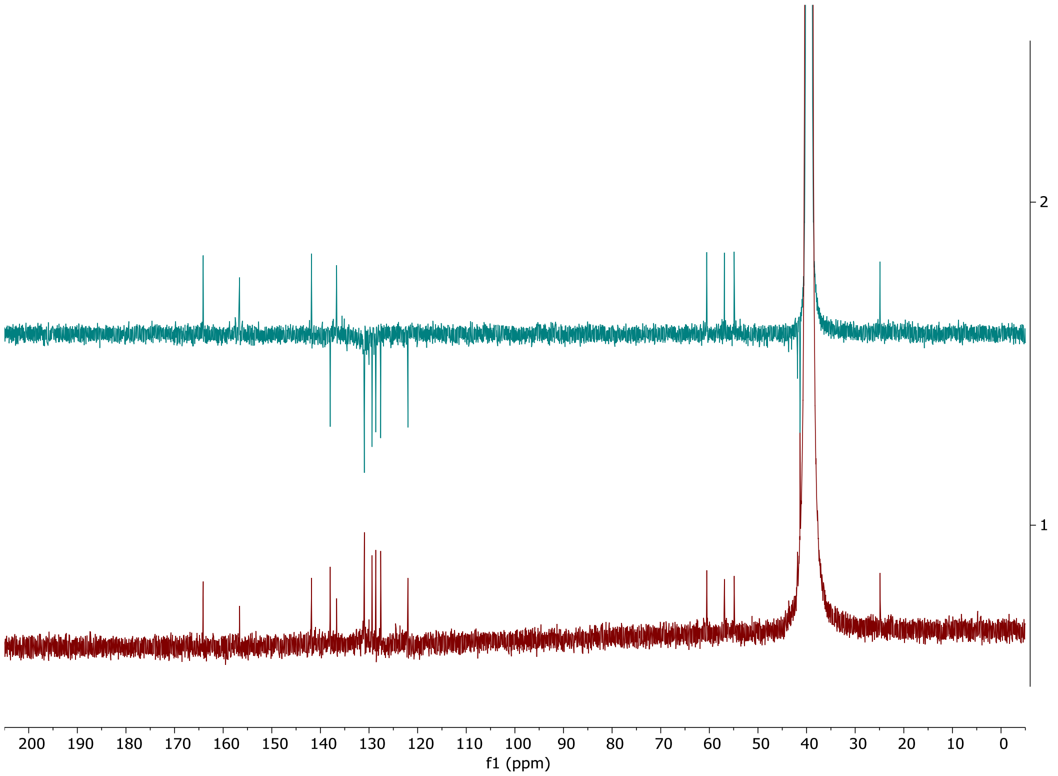


**Figure S17**. APT-NMR spectrum (top) compared to $^{13}$C-NMR experiment (bottom), of compound **1a** (101 MHz, DMSO-$d_6$).

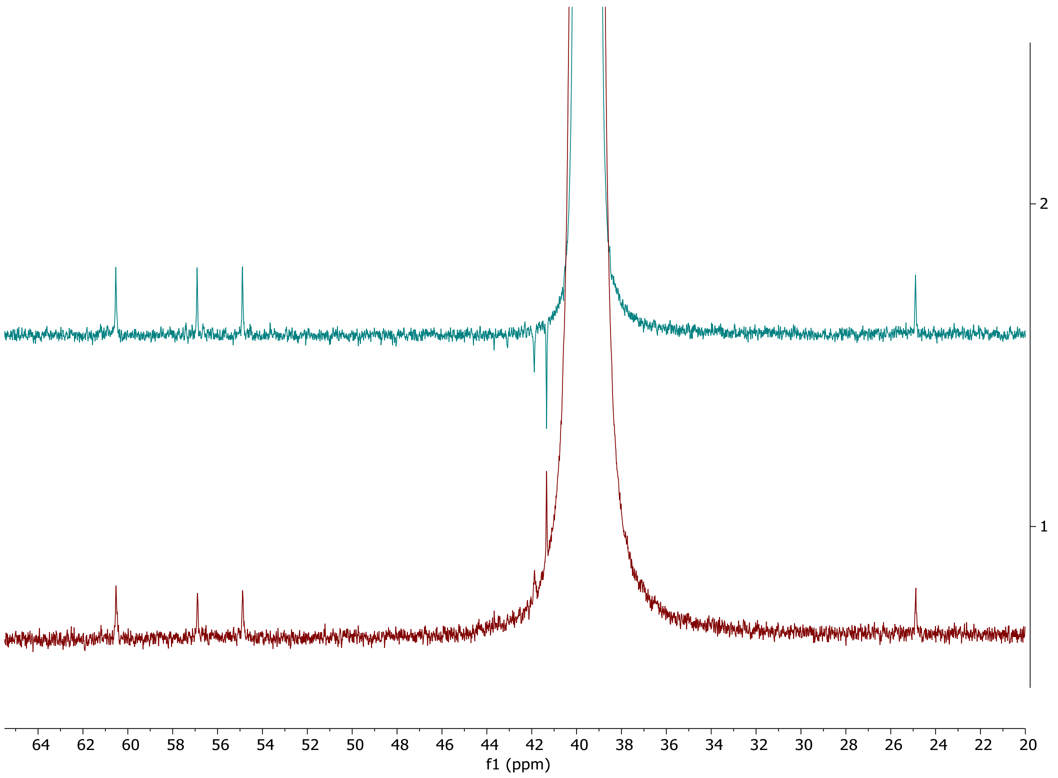


**Figure S18**. Upfield zoom of APT-NMR spectrum (top) compared to $^{13}C$-NMR experiment (bottom), of compound **1a** (101 MHz, DMSO-$d_6$)

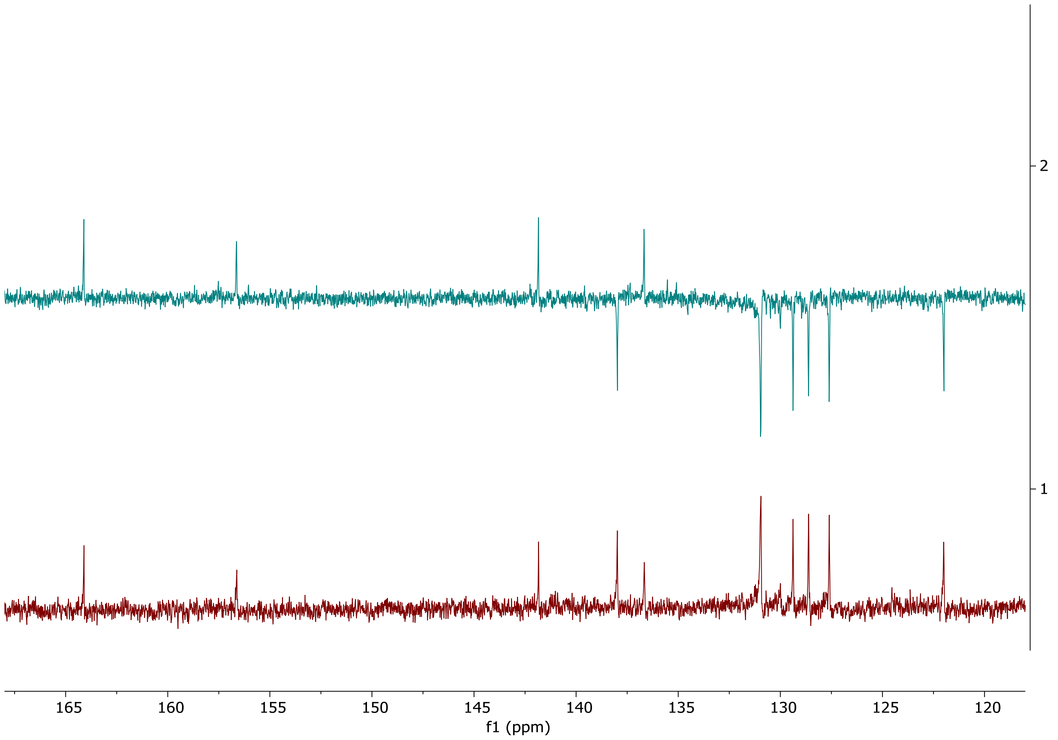


**Figure S19**. Downfield zoom of APT-NMR spectrum (top) compared to $^{13}C$-NMR experiment (bottom), of compound **1a** (101 MHz, DMSO-$d_6$)

The two NMR experiments correlate well, and the phases of the signals in the APT-experiment match our expectations.

## 7.2 Synthesis of Ligands

2,2'-([2,2'-Bipyridine]-6,6'-diyl)dibenzaldehyde (**A**) was synthesized according to a known procedure.[S2]

**6,10,14-Trimethyl-6,10,14-triaza-2,3(2,6)-dipyridina-1,4(1,2)-dibenzenacyclopentadecaphane (L1)**

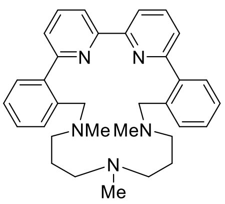


**L1**

2,2'-([2,2'-Bipyridine]-6,6'-diyl)dibenzaldehyde (**A**, 500 mg, 1.37 mmol, 1.0 equiv.) was suspended into THF (25 mL) in a round-bottom flask equipped with a large magnetic stir bar. The mixture was degassed by bubbling argon through, and sodium triacetoxyborohydride (929 mg, 4.38 mmol, 3.2 equiv.) was added**.** Then methylbis(3-methylaminopropyl)amin (309 mg, 1.78 mmol, 1.3 equiv.) in THF (5 mL) was added slowly using a syringe pump at 1 mL/h, whereafter the reaction mixture was allowed to stir at room temperature. After 45 h, a saturated solution of $NaHCO_3$ (15 mL) was added and allowed to stir for 1 h. Water (20 mL) and a 30% sodium hydroxide solution (5 mL) were added to the mixture. The corresponding mixture was extracted with EtOAc (4 × 30 mL). The combined organic layers were extracted with aqueous HCl (4 M, 4 × 10 mL), and the combined aqueous HCl layers were adjusted to pH 14 by the addition of a 30% sodium hydroxide solution followed by extraction with EtOAc (4 × 30 mL). The combined organic layers were dried over $Na_2SO_4$, filtered, and evaporated to give the crude ligand as a pale-yellow oil, which crystallized upon standing at 2 °C. The crude product was purified by recrystallization in EtOH to give the product as a colorless solid (246 mg, 35% yield). An additional crop of ligand, which was nearly as pure, could be obtained by crystallization and an additional recrystallization of the mother liquor.

**$^1$H NMR** (400 MHz, $CDCl_3$) ($\delta$) ppm: 8.48 (d, $J$ = 7.9 Hz, 2H), 7.85 (t, $J$ = 7.8 Hz, 2H), 7.45 – 7.33 (m, 10H), 3.96 (s, 4H), 2.16 (t, $J$ = 7.2 Hz, 4H), 2.02 (s, 3H), 1.89 (s, 6H), 1.88 – 1.77 (m, 4H), 1.22 – 1.10 (m, 4H). **$^{13}$C NMR** (101 MHz, $CDCl_3$) ($\delta$) 160.6 (2C), 155.0 (2C), 141.3 (2C), 138.0 (2C), 137.4 (2C), 131.4 (2C), 130.7 (2C), 128.0 (2C), 127.5 (2C), 123.7(2C), 118.8 (2C), 61.2 (2C), 57.2 (2C), 53.6 (2C), 42.8, 40.7 (2C), 23.7 (2C). **HRMS** (ESI$^+$) m/z calcd. for $C_{33}H_{40}N_5$ $[M+H]^+$: 506.3278, found: 506.3293.

**6,14-Dimethyl-10-oxa-6,14-diaza-2,3(2,6)-dipyridina-1,4(1,2)-dibenzenacyclopentadecaphane (L2)**

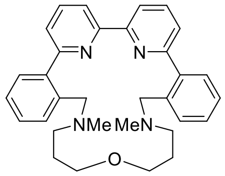


**L2**

2,2'-([2,2'-Bipyridine]-6,6'-diyl)dibenzaldehyde (**A**, 269 mg, 0.74 mmol, 1.0 equiv.) was suspended into THF (11 mL) in a round-bottom flask equipped with a large magnetic stir bar. The mixture was degassed by bubbling argon through, and sodium triacetoxyborohydride (500 mg, 2.36 mmol, 3.2 equiv.) was added**.** Then 3,3'-oxybis(*N*-methylpropan-1-amine) (4.17, 130 mg, 0.81 mmol, 1.1 equiv.) in THF (5 mL) was added slowly using a syringe pump at 1 mL/h, whereafter the reaction mixture was allowed to stir at room temperature. After 72 h, $H_2O$ (10 mL) was added, and the mixture was allowed to stir for 1 h. Water (20 mL) and an aqueous 30% sodium hydroxide solution (5 mL) were added to the mixture. The corresponding mixture was extracted with $CH_2Cl_2$ (5 × 20 mL). The combined organic layers were dried over $Na_2SO_4$, filtered, and evaporated to give the crude ligand as a pale-yellow solid. The crude product was purified by FCC on silica gel (40 g) eluting with a gradient from 10% $CH_2Cl_2$ and 1% $Et_3N$ in pentane to 30% $CH_2Cl_2$ and 1% $Et_3N$ in pentane, yielding the pure product as a colorless solid (173 mg, 48% yield).

**$^1$H NMR** (400 MHz, $CDCl_3$) ($\delta$) ppm: 8.48 (dd, $J$ = 7.9, 1.1 Hz, 2H), 7.82 (t, $J$ = 7.8 Hz, 2H), 7.46 (dd, $J$ = 7.1, 1.7 Hz, 2H), 7.42 – 7.30 (m, 8H), 3.87 (s, 4H), 2.79 (t, $J$ = 7.0 Hz, 4H), 2.15 (t, $J$ = 7.0 Hz, 4H), 1.90 (s, 6H), 1.26 (p, $J$ = 7.0 Hz, 4H). **$^{13}$C NMR** (101 MHz, $CDCl_3$) ($\delta$) ppm: 160.2 (2C), 155.4 (2C), 141.7 (2C), 137.7 (2C), 136.9 (2C), 131.2 (2C), 130.7 (2C), 127.9 (2C), 127.6 (2C), 123.5 (2C), 118.9 (2C), 68.5 (2C), 61.4 (2C), 55.0 (2C), 40.4 (2C), 27.3 (2C). **HRMS** (ESI$^+$) m/z calcd. for $C_{32}H_{37}N_4O$ $[M+H]^+$: 493.2962, found: 493.2965.

**6,13-Dimethyl-6,13-diaza-2,3(2,6)-dipyridina-1,4(1,2)-dibenzenacyclotetradecaphane (L3)**

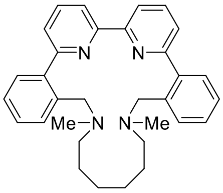


L3

2,2'-([2,2'-Bipyridine]-6,6'-diyl)dibenzaldehyde (**A**, 364 mg, 1.0 mmol, 1.0 equiv.) was suspended into THF (15 mL) in a round-bottom flask equipped with a large magnetic stir bar. The mixture was degassed by bubbling argon through, and sodium triacetoxyborohydride (678 mg, 3.2 mmol, 3.2 equiv.) was added. Then *N*,*N*'-dimethyl-1,6-hexanediamine (188 mg, 1.3 mmol, 1.3 equiv.) in THF (5 mL) was added slowly using a syringe pump at 1 mL/h, whereafter the reaction mixture was allowed to stir at room temperature. After 72 h, a saturated solution of $NaHCO_3$ (10 mL) was added and allowed to stir for 1 h. Water (20 mL) and a 30% sodium hydroxide solution (5 mL) were added the mixture. The corresponding mixture was extracted with EtOAc (4 × 30 mL). The combined organic layers were extracted with aqueous HCl (4 M, 4 × 10 mL), and the combined aqueous HCl layers were adjusted to pH 14 by the addition of a 30% sodium hydroxide solution followed by extraction with EtOAc (4 × 30 mL). The combined organic layers were dried over $Na_2SO_4$, filtered, and evaporated to give the crude ligand as a pale-yellow solid. The crude product was purified by FCC on silica gel (15 g) eluting with a gradient from 5% $CH_2Cl_2$ and 1% $Et_3N$ in pentane to 20% $CH_2Cl_2$ and 1% $Et_3N$ in pentane, yielding the pure product as a colorless solid (64 mg, 13% yield).

**$^1$H NMR** (400 MHz, $CDCl_3$) (*δ*) ppm: 8.54 (dd, *J* = 7.9, 1.0 Hz, 2H), 7.82 (t, *J* = 7.8 Hz, 2H), 7.49 (dd, *J* = 7.0, 1.6 Hz, 2H), 7.45 – 7.31 (m, 8H), 3.89 (bs, 2H), 2.05 (t, *J* = 7.2 Hz, 4H), 1.93 (s, 6H), 0.89 – 0.77 (m, 4H), 0.70 – 0.58 (m, 4H). **$^{13}$C NMR** (101 MHz, $CDCl_3$) (*δ*) ppm 160.1 (2C), 154.9 (2C), 141.5 (2C), 137.9 (2C), 137.1 (2C), 131.9 (2C), 130.7 (2C), 127.9 (2C), 127.6 (2C), 123.8 (2C), 118.6 (2C), 59.9 (2C), 58.9 (2C), 42.2 (2C), 27.8 (2C), 27.6 (2C). **HRMS** ($ESI^+$) m/z calcd. for $C_{32}H_{37}N_4$ $[M+H]^+$: 477.3013, found: 477.3020.

## 7.3 Synthesis of Starting Materials

1-Methylimidazolium tetrafluoroborate [$(HMIm)BF_4$] was synthesized according to a literature procedure from Holbrey and Seddon.[S41]

***tert*-Butyl (3-hydroxypropyl)(methyl)carbamate (B)**

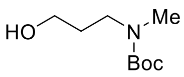


B

**B** was synthesized following a modified procedure from Prasad and co-workers.[S42] In a 50 mL round bottom flask equipped with a stir bar $(Boc)_2O$ (10.9 g, 50 mmol, 1.0 equiv.) and [$(HMIm)BF_4$] (85 mg, 0.5 mmol, 1.0 mol%) were mixed under an inert atmosphere at 35 °C. 3-Methylamino-1-propanol (4.86 mL, 4.46 g, 50 mmol, 1.0 equiv.) was added dropwise, and the mixture was stirred for 10 min. The mixture was transferred to a larger flask and diluted with $Et_2O$ (100 mL). The corresponding cloudy solution was filtered and concentrated to give the crude product as a slightly yellow oil. The crude product was purified by FCC on silica gel using an eluent from 25% EtOAc in pentane to 30% EtOAc in pentane to give the product as a colorless oil (9.19 g, 97% yield).

**$^1$H NMR** (400 MHz, $CDCl_3$) (*δ*) ppm: 3.87 – 3.47 (m, 3H), 3.48 – 3.24 (m, 2H), 2.82 (d, *J* = 3.5 Hz, 3H), 1.67 (bs, 2H), 1.45 (d, *J* = 5.1 Hz, 9H). **$^{13}$C NMR** (101 MHz, $CDCl_3$) (*δ*) ppm: 157.4, 80.1, 58.2, 44.3, 34.3, 29.8, 28.5 (3C). **HRMS** ($ESI^+$) m/z calcd. for $C_9H_{19}NNaO_3$ $[M+Na]^+$: 212.1257, found: 212.1267.

Spectroscopic data were in accordance with the literature.[S42]

**3-((*tert*-Butoxycarbonyl)(methyl)amino)propyl methanesulfonate (C)**

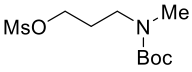


C

To a stirred solution of **B** (2.50 g, 13.2 mmol, 1.0 equiv.) in dry $CH_2Cl_2$ (15 mL) under an inert atmosphere at 0 °C, triethylamine (3.7 mL, 2.67 g, 26.4 mmol, 2.0 equiv.) was added followed by the dropwise addition of methanesulfonyl chloride (1.0 mL, 1.51 g, 13.2 mmol, 1.0 equiv.). After the reaction was stirred for 4 h at RT,

full conversion was observed by TLC (1:1, EtOAc : pentane), and a saturated aqueous solution of $NaHCO_3$ (20 mL) was added. The layers were separated, and the aqueous layer was extracted with $CH_2Cl_2$ (3 × 20 mL). The combined organic layers were dried over $Na_2SO_4$, filtered, and concentrated in vacuo to give the product as a colorless oil (3.17g, 90% yield).

**$^1$H NMR** (400 MHz, $CDCl_3$) (*δ*) ppm: 4.25 (t, *J* = 6.2 Hz, 2H), 3.35 (t, *J* = 6.8 Hz, 2H), 3.02 (s, 3H), 2.87 (s, 3H), 1.98 (p, *J* = 6.5 Hz, 2H), 1.46 (s, 9H). **$^{13}$C NMR** (101 MHz, $CDCl_3$) (*δ*) ppm: 155.9, 79.9, 67.7, 45.3, 37.5, 34.7, 28.5 (3C), 27.8.
Spectroscopic data were in accordance with the literature.[S43]

**Di-*tert*-butyl (oxybis(propane-3,1-diyl))bis(methylcarbamate) (D)**

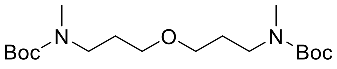


**D**

In a 25 mL reaction flask with a PTFE-inlay and screw cap equipped with a magnetic stir bar, **B** (708 mg, 3.74 mmol, 1.0 equiv.), **C** (1.0 g, 3.74 mmol, 1.0 equiv.), and $Bu_4NBr$ (362 mg, 1.12 mmol, 0.3 equiv.) was solubilized in $Et_2O$ (15 mL). To this solution, an aqueous solution of NaOH (7.5 mL, 50% W/V) was added, and the flask was flushed with Ar. The flask was closed and allowed to stir vigorously. After 72 h, full consumption of the mesylate was observed (TLC, 20% EtOAc in pentane) and the mixture was extracted with EtOAc (3 × 15 mL). The combined organic layers were dried over $Na_2SO_4$, filtered, and concentrated in vacuo to yield the crude product as a slightly yellow oil. The product was purified by FCC on silica gel (110 g), eluting with a gradient from 5% EtOAc in pentane to 20% EtOAc in pentane, yielding the product as a colorless oil (1.19 g, 88% yield).

**$^1$H NMR** (400 MHz, $CDCl_3$) (*δ*) ppm: 3.40 (t, *J* = 6.3 Hz, 4H), 3.33 – 3.22 (m, 4H), 2.84 (s, 6H), 1.77 (p, *J* = 6.7 Hz, 4H), 1.45 (s, 18H). **$^{13}$C NMR** (101 MHz, $CDCl_3$) (*δ*) ppm: 155.9 (2C), 79.3 (2C), 68.6 (2C), 46.4 (2C), 46.1 (2C), 34.6 (2C), 28.6 (6C). **HRMS** ($ESI^+$) m/z calcd. for $C_{18}H_{37}N_2O_5$ $[M+H]^+$: 361.2697, found: 361.2699.

**3,3'-Oxybis(N-methylpropan-1-amine) (E)**

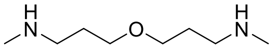


**E**

In a flame-dried flask equipped with a stir bar, **D** (360 mg, 1.0 mmol, 1.0 equiv.) was dissolved in dry $CH_2Cl_2$ (15 mL) under an atmosphere of Ar. The solution was cooled to 0 °C, and TFA (1.16 mL, 15 mmol, 15 equiv.) was added dropwise, and the mixture was allowed to warm to RT ON. The mixture was evaporated under reduced pressure to remove excess TFA and the residue was taken up in $CH_2Cl_2$ and washed with aq. 4 M NaOH. The aqueous phases were extracted with $CH_2Cl_2$, and the combined organic layers were dried over $Na_2SO_4$, filtered, and concentrated in vacuo to yield the crude product as a colorless oil which was used without purification (102 mg, 64% yield).

**$^1$H NMR** (400 MHz, $CDCl_3$) (*δ*) ppm: 3.46 (t, *J* = 6.2 Hz, 4H), 2.64 (t, *J* = 6.8 Hz, 4H), 2.41 (s, 6H), 1.74 (p, *J* = 6.6 Hz, 4H), 1.49 (s, 2H). **$^{13}$C NMR** (101 MHz, $CDCl_3$) (*δ*) ppm: 69.6 (2C), 49.7 (2C), 36.7 (2C), 29.9 (2C). **HRMS** ($ESI^+$) m/z calcd. for $C_8H_{21}N_2O$ $[M+H]^+$: 161.1648, found: 161.1655.

**Tetrabutylammonium formate (F)**

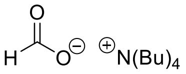


**F**

A schlenk tube charged with a stir bar was flame dried and cycled into the Schlenk line. A solution of tetrabutylammonium hydroxide (1 M in MeOH, 5 mL, 5 mmol, 1.0 equiv.) was added to the tube, followed by dropwise addition of degassed formic acid (0.190 mL, 5 mmol, 1.0 equiv.). After stirring overnight, the voatiles were removed under vaccum first at room temperature and then at 60 °C. The compound was dried under high vacuum at 60 °C for 72 h to give the dry product as a colorless solid (1.43 g, quant.).

Note: the compound is deliquescent and is only handled under an inert atmosphere using standard Schlenk techniques or in an argon-filled glovebox.

**$^1$H NMR** (400 MHz, $CDCl_3$) ($\delta$) ppm: 8.76 (s, 1H), 3.36 – 3.25 (m, 8H), 1.63 (p, $J$ = 7.8 Hz, 8H), 1.42 (h, $J$ = 7.4 Hz, 8H), 0.98 (t, $J$ = 7.3 Hz, 12H). **$^{13}$C NMR** (101 MHz, $CDCl_3$) (δ) ppm: 167.6, 58.9 (4C), 24.1 (4C), 19.8 (4C), 13.8 (4C). **IR** (ATR, $cm^{-1}$) $\tilde{\nu}$ = 2960 (m), 2935 (w), 1599 (s), 1464 (w), 1331 (m), 1057 (w), 1028 (w), 883 (m), 746 (m), 615 (m). Spectroscopic data is in agreement with literature data.[S44,S45]

## 8. References


S1. Gottlieb, H. E.; Kotlyar, V.; Nudelman, A., NMR Chemical Shifts of Common Laboratory Solvents as Trace Impurities. *J. Org. Chem.* **1997,** *62*, 7512–7515.

S2. Rønne, M. H.; Cho, D.; Madsen, M. R.; Jakobsen, J. B.; Eom, S.; Escoudé, É.; Hammershøj, H. C. D.; Nielsen, D. U.; Pedersen, S. U.; Baik, M.-H.; Skrydstrup, T.; Daasbjerg, K., Ligand-Controlled Product Selectivity in Electrochemical Carbon Dioxide Reduction Using Manganese Bipyridine Catalysts. *J. Am. Chem. Soc.* **2020,** *142*, 4265–4275.

S3. *CrysAlisPRO, Oxford Diffraction /Agilent Technologies UK Ltd, Yarnton, England*

S4. Dolomanov, O. V.; Bourhis, L. J.; Gildea, R. J.; Howard, J. A.; Puschmann, H., OLEX2: A Complete Structure Solution, Refinement and Analysis Program. *J. Appl. Cryst.* **2009,** *42*, 339–341.

S5. Sheldrick, G. M., SHELXT–Integrated Space-Group and Crystal-Structure Determination. *Acta. CrysT.* **2015,** *A71*, 3–8.

S6. Sheldrick, G., A short History of SHELX. *Acta. Cryst.* **2008,** *A64*, 112–122.

S7. Iftimie, R.; Minary, P.; Tuckerman, M. E., *Ab initio* Molecular Dynamics: Concepts, Recent Developments, and Future Trends. *Proc. Natl. Acad. Sci.* **2005,** *102*, 6654–6659.

S8. Kühne, T. D.; Iannuzzi, M.; Ben, M. D.; Rybkin, V. V.; Seewald, P.; Stein, F.; Laino, T.; Khaliullin, R. Z.; Schütt, O.; Schiffmann, F., CP2K: An Electronic Structure and Molecular Dynamics Software Package - Quickstep: Efficient and Accurate Electronic Structure Calculations. *J. Chem. Phys.* **2020,** *152*, 194103.

S9. Perdew, J. P.; Burke, K.; Ernzerhof, M., Generalized Gradient Approximation Made Simple. *Phys. Rev. Lett.* **1996,** *77*, 3865–3868.

S10. Godbout, N.; Salahub, D. R.; Andzelm, J.; Wimmer, E., Optimization of Gaussian-Type Basis Sets for Local Spin Density Functional Calculations. Part I. Boron through Neon, Optimization Technique and Validation. *Can. J. Chem.* **1992,** *70*, 560–571.

S11. Bussi, G.; Donadio, D.; Parrinello, M., Canonical Sampling through Velocity Rescaling. *J. Chem. Phys.* **2007,** *126*, 014101.

S12. Goedecker, S.; Teter, M.; Hutter, J., Separable Dual-space Gaussian Pseudopotentials. *Phys. Rev. B* **1996,** *54*, 1703–1710.

S13. Grimme, S.; Antony, J.; Ehrlich, S.; Krieg, H., A Consistent and Accurate *ab initio* Parametrization of Density Functional Dispersion Correction (DFT-D) for the 94 Elements H-Pu. *J. Chem. Phys.* **2010,** *132*, 154104.

S14. Martínez, L.; Andrade, R.; Birgin, E. G.; Martínez, J. M., PACKMOL: A Package for Building Initial Configurations for Molecular Dynamics Simulations. *J. Comput. Chem.* **2009,** *30*, 2157–2164.

S15. Parr, R. G., Density Functional Theory of Atoms and Molecules. In *Horizons of Quantum Chemistry*, Springer Netherlands: Dordrecht, 1980; pp 5–15.

S16. Frisch, M.; Trucks, G.; Schlegel, H.; Scuseria, G.; Robb, M.; Cheeseman, J.; Scalmani, G.; Barone, V.; Petersson, G.; Nakatsuji, H., Gaussian 16 Rev. C. 01, Wallingford, CT. 2016.

S17. Staroverov, V. N.; Scuseria, G. E.; Tao, J.; Perdew, J. P., Comparative Assessment of a New Nonempirical Density Functional: Molecules and Hydrogen-bonded Complexes. *J. Chem. Phys.* **2003,** *119*, 12129–12137.

S18. Weigend, F.; Ahlrichs, R., Balanced Basis Sets of Split Valence, Triple Zeta Valence and Quadruple Zeta Valence Quality for H to Rn: Design and Assessment of Accuracy. *Phys. Chem. Chem. Phys.* **2005,** *7*, 3297–3305.

S19. Weigend, F., Accurate Coulomb-fitting Basis Sets for H to Rn. *Phys. Chem. Chem. PhyS.* **2006,** *8*, 1057–1065.

S20. Grimme, S.; Ehrlich, S.; Goerigk, L., Effect of the Damping Function in Dispersion Corrected Density Functional Theory. *J. Comput. Chem.* **2011,** *32*, 1456–1465.

S21. Johnson, B. G.; Fisch, M. J., An Implementation of Analytic Second Derivatives of the Gradient-corrected Density Functional Energy. *J. Chem. Phys.* **1994,** *100*, 7429–7442.

S22. Stratmann, R. E.; Burant, J. C.; Scuseria, G. E.; Frisch, M. J., Improving Harmonic Vibrational Frequencies Calculations in Density Functional Theory. *J. Chem. Phys.* **1997,** *106*, 10175–10183.

S23. Marenich, A. V.; Cramer, C. J.; Truhlar, D. G., Universal Solvation Model Based on Solute Electron Density and on a Continuum Model of the Solvent Defined by the Bulk Dielectric Constant and Atomic Surface Tensions. *J. Phys. Chem. B* **2009,** *113*, 6378–6396.
S24. Baerends, E. J. ADF 2019, SCM, Theoretical Chemistry, Vrije Universiteit, Amsterdam, The Netherlands, http://www.scm.com.
S25. Keal, T. W.; Tozer, D. J., The Exchange-correlation Potential in Kohn–Sham Nuclear Magnetic Resonance Shielding Calculations. *J. Chem. Phys.* **2003,** *119*, 3015–3024.
S26. Chong, D. P.; Van Lenthe, E.; Van Gisbergen, S.; Baerends, E. J., Even-tempered Slater-type Orbitals Revisited: From Hydrogen to Krypton. *J. Comput. Chem.* **2004,** *25*, 1030–1036.
S27. Klamt, A.; Schüürmann, G., COSMO: a New Approach to Dielectric Screening in Solvents with Explicit Expressions for the Screening Energy and Its Gradient. *J. Chem. Soc., Perkin Trans. 2* **1993**, 799–805.
S28. Klamt, A., Calculation of UV/Vis Spectra in Solution. *J. Phys. Chem. B* **1996,** *100*, 3349–3353.
S29. Pye, C. C.; Ziegler, T., An Implementation of the Conductor-like Screening Model of Solvation within the Amsterdam Density Functional Package. *Theor. Chem. Acc.* **1999,** *101*, 396–408.
S30. Lenthe, E. v.; Baerends, E.-J.; Snijders, J. G., Relativistic Regular Two-component Hamiltonians. *J. Chem. Phys.* **1993,** *99*, 4597–4610.
S31. Van Lenthe, E.; Van Leeuwen, R.; Baerends, E.; Snijders, J., Relativistic Regular Two-component Hamiltonians. *Int. J. Quantum Chem.* **1996,** *57*, 281–293.
S32. Van Lenthe, E.; Ehlers, A.; Baerends, E.-J., Geometry Optimizations in the Zero Order Regular Approximation for Relativistic Effects. *J. Chem. Phys.* **1999,** *110*, 8943–8953.
S33. Van Lenthe, E.; Baerends, E.-J.; Snijders, J. G., Relativistic Total Energy Using Regular Approximations. *J. Chem. Phys.* **1994,** *101*, 9783–9792.
S34. Van Lenthe, E. v.; Snijders, J.; Baerends, E., The Zero-order Regular Approximation for Relativistic Effects: The Effect of Spin–orbit Coupling in Closed Shell Molecules. *J. Chem. Phys.* **1996,** *105*, 6505–6516.
S35. Ditchfield, R., Self-consistent Perturbation Theory of Diamagnetism: I. A Gauge-invariant LCAO Method for NMR Chemical Shifts. *Mol. Phys.* **1974,** *27*, 789–807.
S36. Walsh, J. J.; Smith, C. L.; Neri, G.; Whitehead, G. F.; Robertson, C. M.; Cowan, A. J., Improving the Efficiency of Electrochemical $CO_2$ Reduction Using Immobilized Manganese Complexes. *Faraday Discuss.* **2015,** *183*, 147–160.
S37. Bourrez, M.; Molton, F.; Chardon-Noblat, S.; Deronzier, A., [Mn(bipyridyl)$(CO)_3$Br]: an Abundant Metal Carbonyl Complex as Efficient Electrocatalyst for $CO_2$ Reduction. *Angew. Chem. Int. Ed.* **2011,** *50*, 9903–9906.
S38. Smieja, J. M.; Sampson, M. D.; Grice, K. A.; Benson, E. E.; Froehlich, J. D.; Kubiak, C. P., Manganese as a Substitute for Rhenium in $CO_2$ Reduction Catalysts: the Importance of Acids. *Inorg. Chem.* **2013,** *52*, 2484–2491.
S39. Sampson, M. D.; Nguyen, A. D.; Grice, K. A.; Moore, C. E.; Rheingold, A. L.; Kubiak, C. P., Manganese Catalysts with Bulky Bipyridine Ligands for the Electrocatalytic Reduction of Carbon Dioxide: Eliminating Dimerization and Altering Catalysis. *J. Am. Chem. Soc.* **2014,** *136*, 5460–5471.
S40. Wu, Q.; Li, M.; He, S.; Xiong, Y.; Zhang, P.; Huang, H.; Chen, L.; Huang, F.; Li, F., The Hangman Effect Boosts Hydrogen Production by a Manganese Terpyridine Complex. *Chem Commun* **2022,** *58*, 5128–5131.
S41. Holbrey, J.; Seddon, K.; Welton, T.; Earle, M.; McCormac, P.; Seddon, K.; Reetz, M.; Wiesenhöfer, W.; Franciò, G.; Leitner, W., Ionic liquids: Perspectives for Organic and Catalytic Reactions. *J. Chem. Soc., Dalton Trans.* **1999,** *99*, 2133–2140.
S42. Sunitha, S.; Kanjilal, S.; Reddy, P. S.; Prasad, R. B. N., An Efficient and Chemoselective Brønsted Acidic Ionic Liquid-catalyzed N-Boc Protection of Amines. *Tetrahedron Lett.* **2008,** *49*, 2527–2532.
S43. Bing, W.; Qi, C., *Biomarin Pharm. Inc.* **2019-07-04**, WO2018US67784 20181228.
S44. Martin, K.; Nõges, J.; Haav, K.; Kadam, S. A.; Pung, A.; Leito, I., Exploring Selectivity of 22 Acyclic Urea-, Carbazole- and Indolocarbazole-Based Receptors towards 11 Monocarboxylates. *Eur. J. Org. Chem.* **2017,** *2017*, 5231–5237.

S45. Murata, T.; Hiyoshi, M.; Ratanasak, M.; Hasegawa, J.-y.; Ema, T., Synthesis of Silyl Formates, Formamides, and Aldehydes via Solvent-free Organocatalytic Hydrosilylation of $CO_2$. *Chem. Commun.* **2020,** *56*, 5783–5786.

## 9. Spectroscopic Data

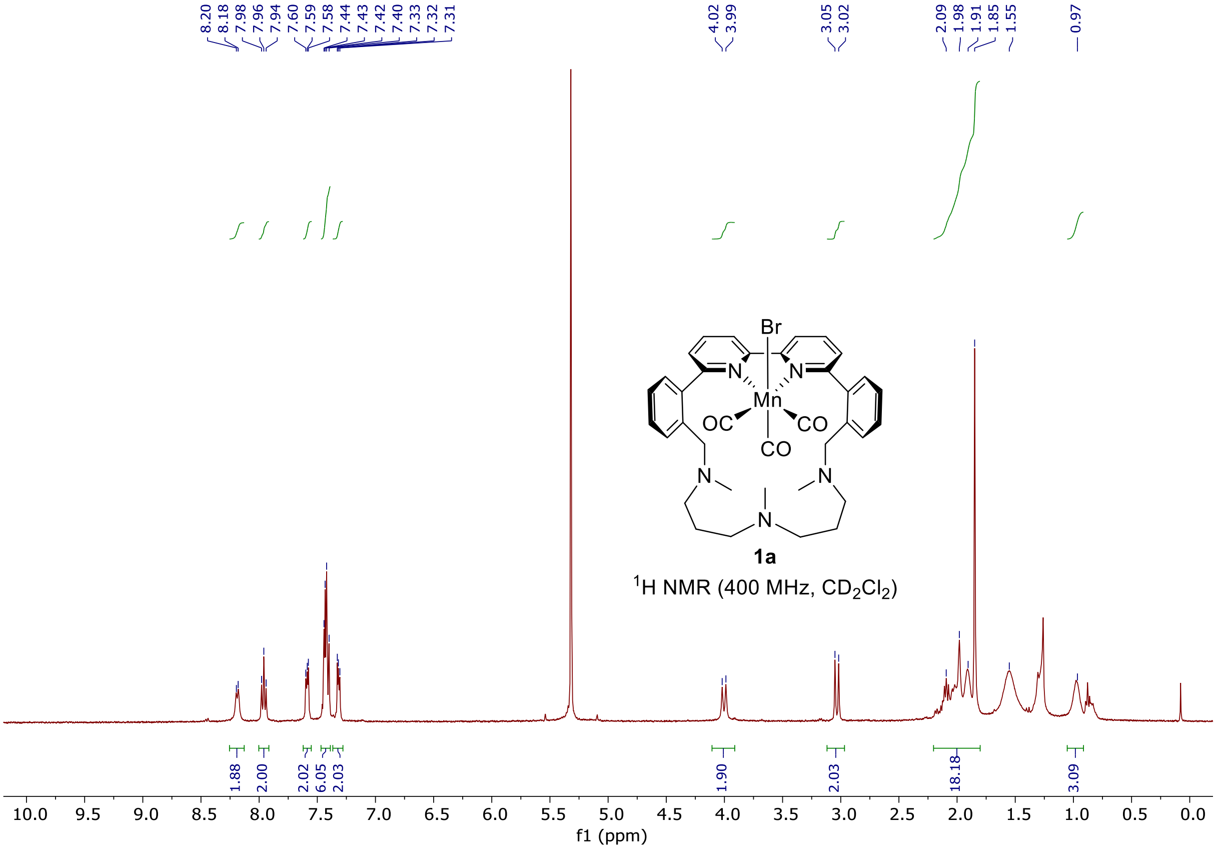


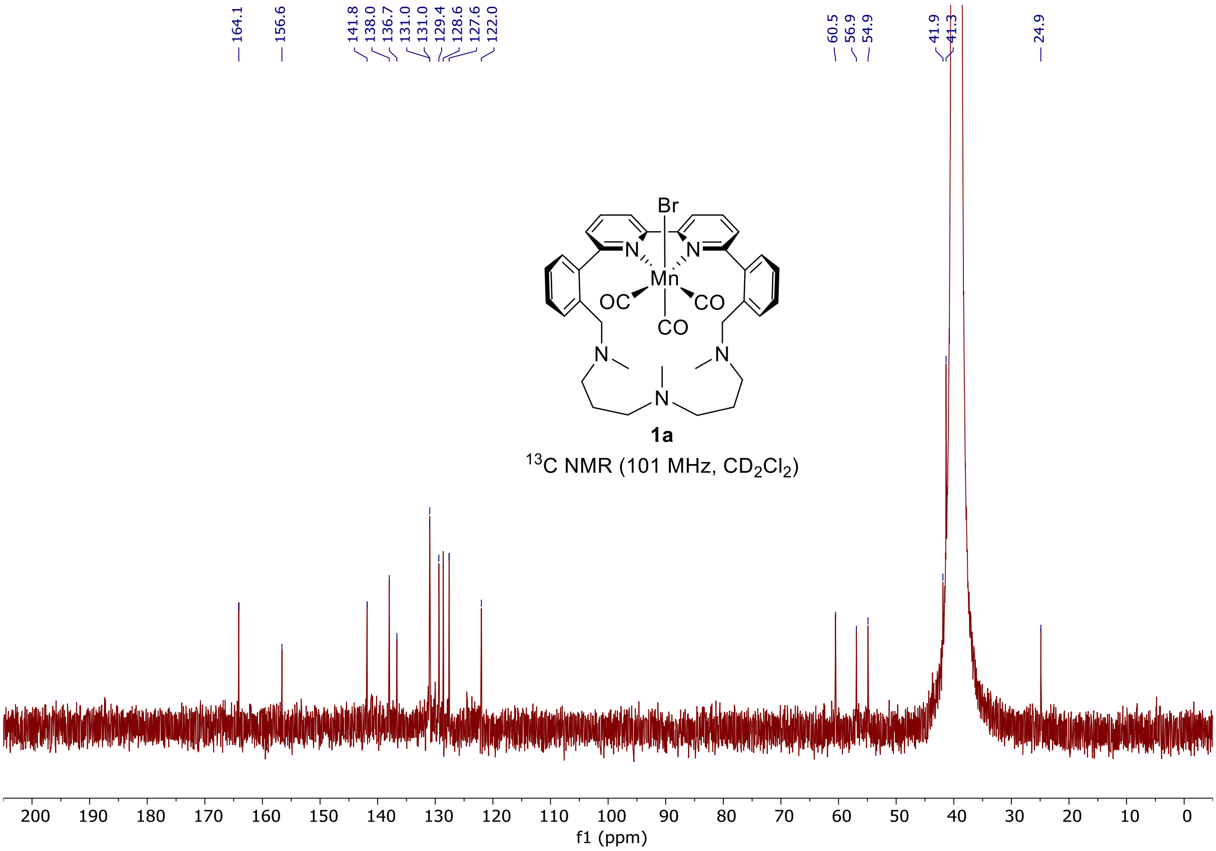

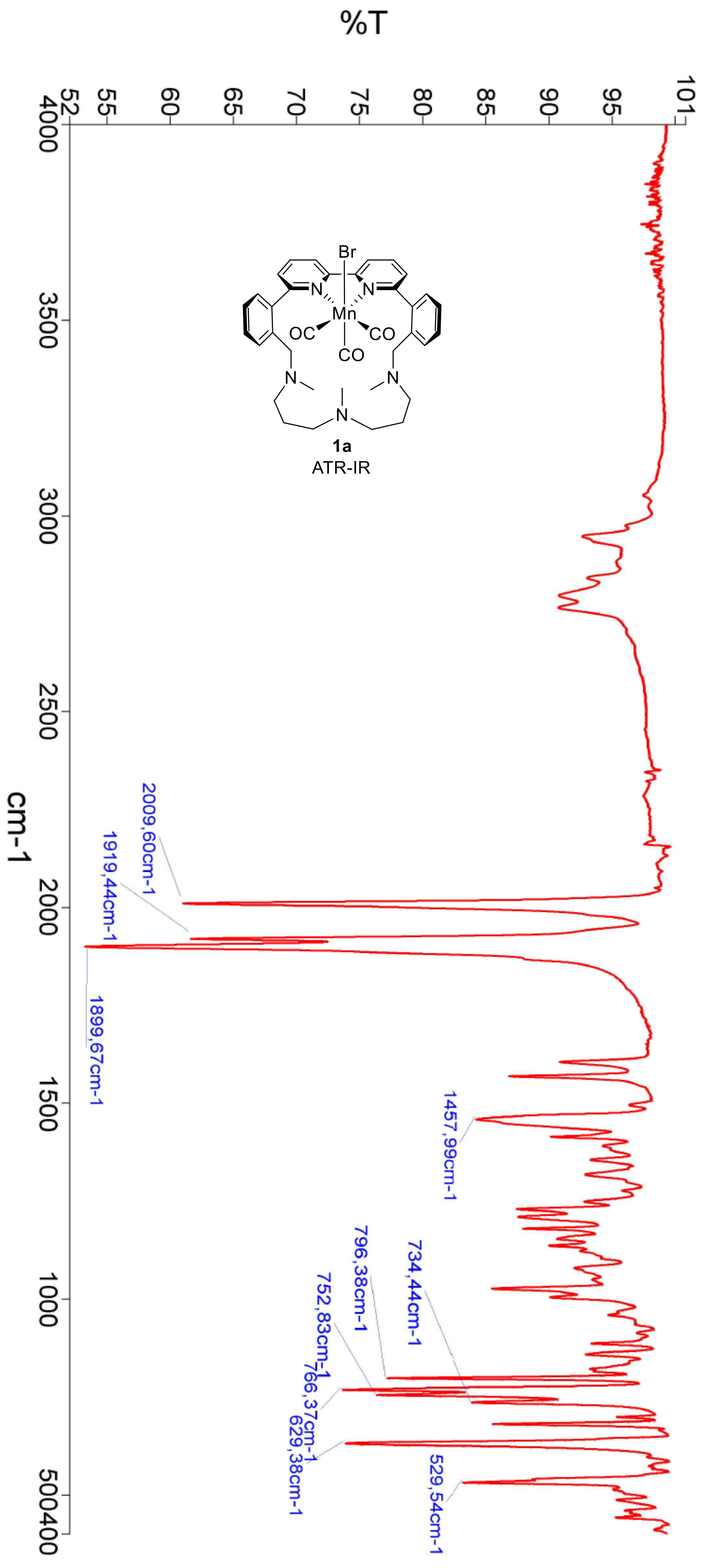
%T
cm-1
1a
ATR-IR
2009,60cm-1
1919,44cm-1
1899,67cm-1
1457,99cm-1
796,38cm-1
734,44cm-1
752,83cm-1
766,37cm-1
629,38cm-1
529,54cm-1

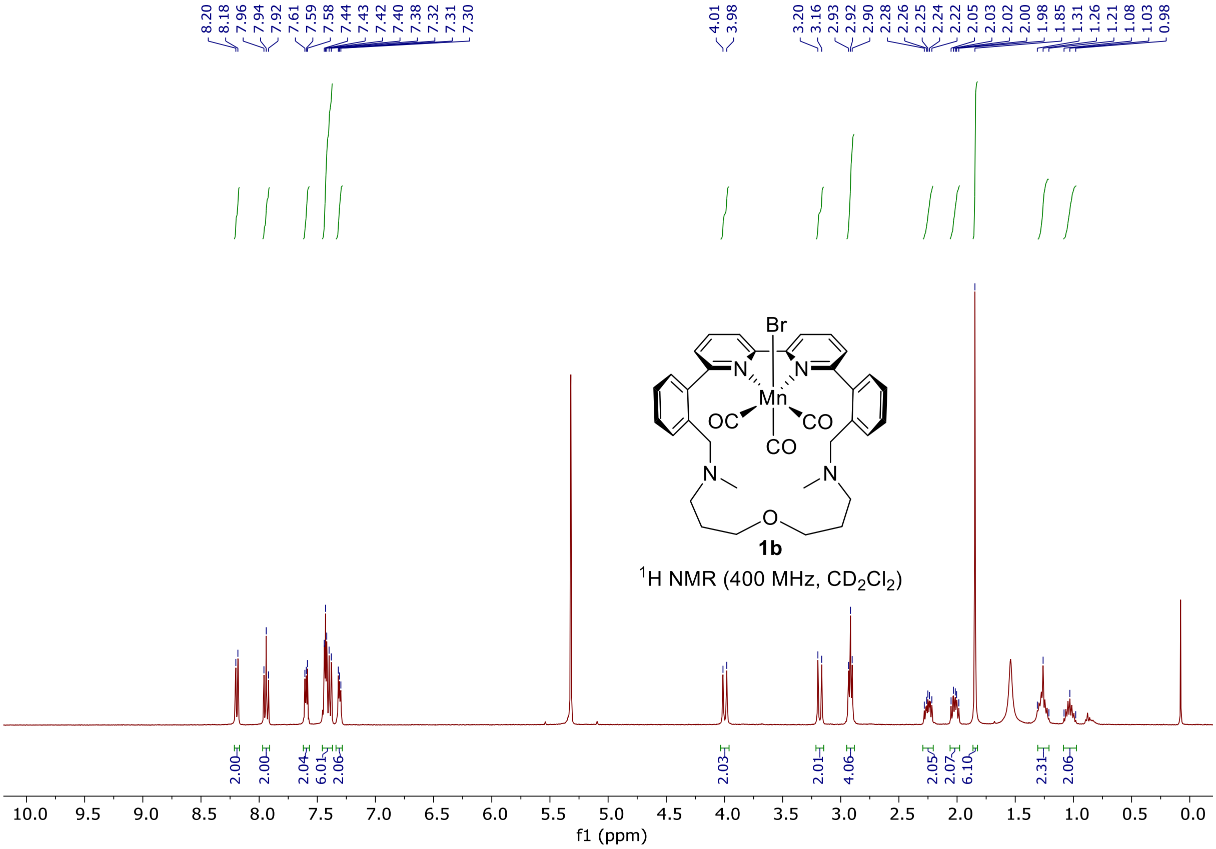
8.20
8.18
7.96
7.94
7.92
7.61
7.59
7.58
7.44
7.43
7.42
7.40
7.38
7.32
7.31
7.30
4.01
3.98
3.20
3.16
2.93
2.92
2.90
2.28
2.26
2.25
2.24
2.22
2.05
2.03
2.02
2.00
1.98
1.85
1.31
1.26
1.21
1.08
1.03
0.98
Br
N
N
Mn
OC
CO
CO
N
N
O
1b
1H NMR (400 MHz, CD2Cl2)
2.00
2.00
2.04
6.01
2.06
2.03
2.01
4.06
2.05
2.07
6.10
2.31
2.06
f1 (ppm)

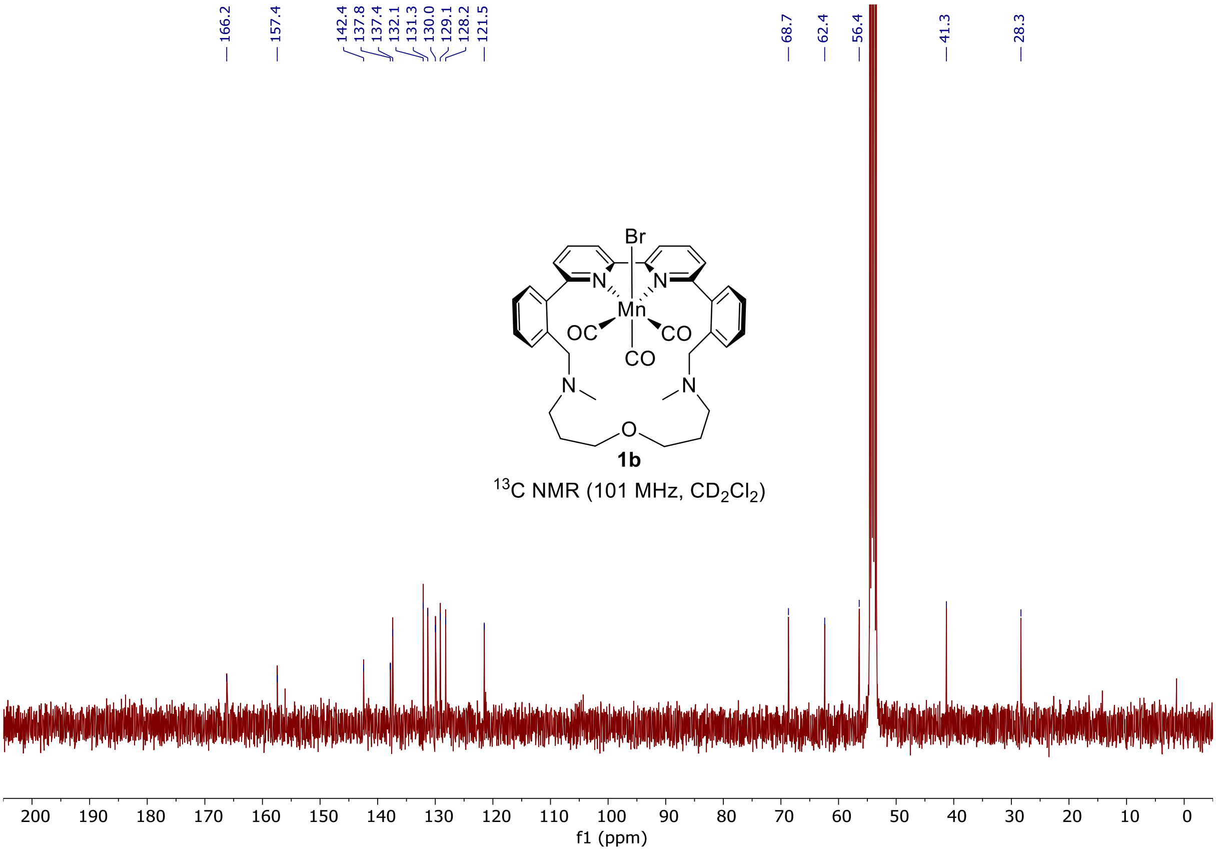
166.2
157.4
142.4
137.8
137.4
132.1
131.3
130.0
129.1
128.2
121.5
68.7
62.4
56.4
41.3
28.3
Br
N
N
Mn
OC
CO
CO
N
N
O
1b
13C NMR (101 MHz, CD2Cl2)
f1 (ppm)

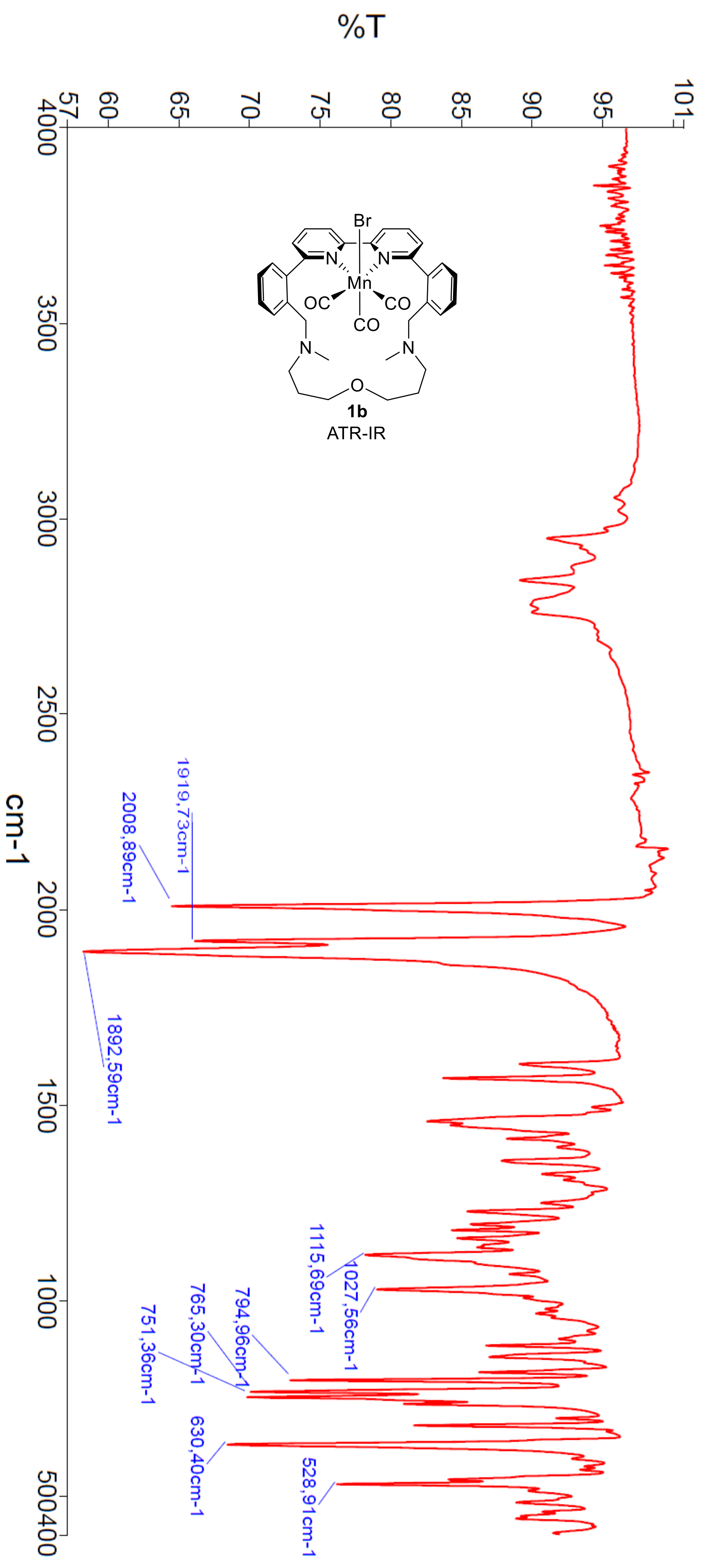
%T
cm-1
1b
ATR-IR
2008,89cm-1
1919,73cm-1
1892,59cm-1
1115,69cm-1
1027,56cm-1
794,96cm-1
765,30cm-1
751,36cm-1
630,40cm-1
528,91cm-1

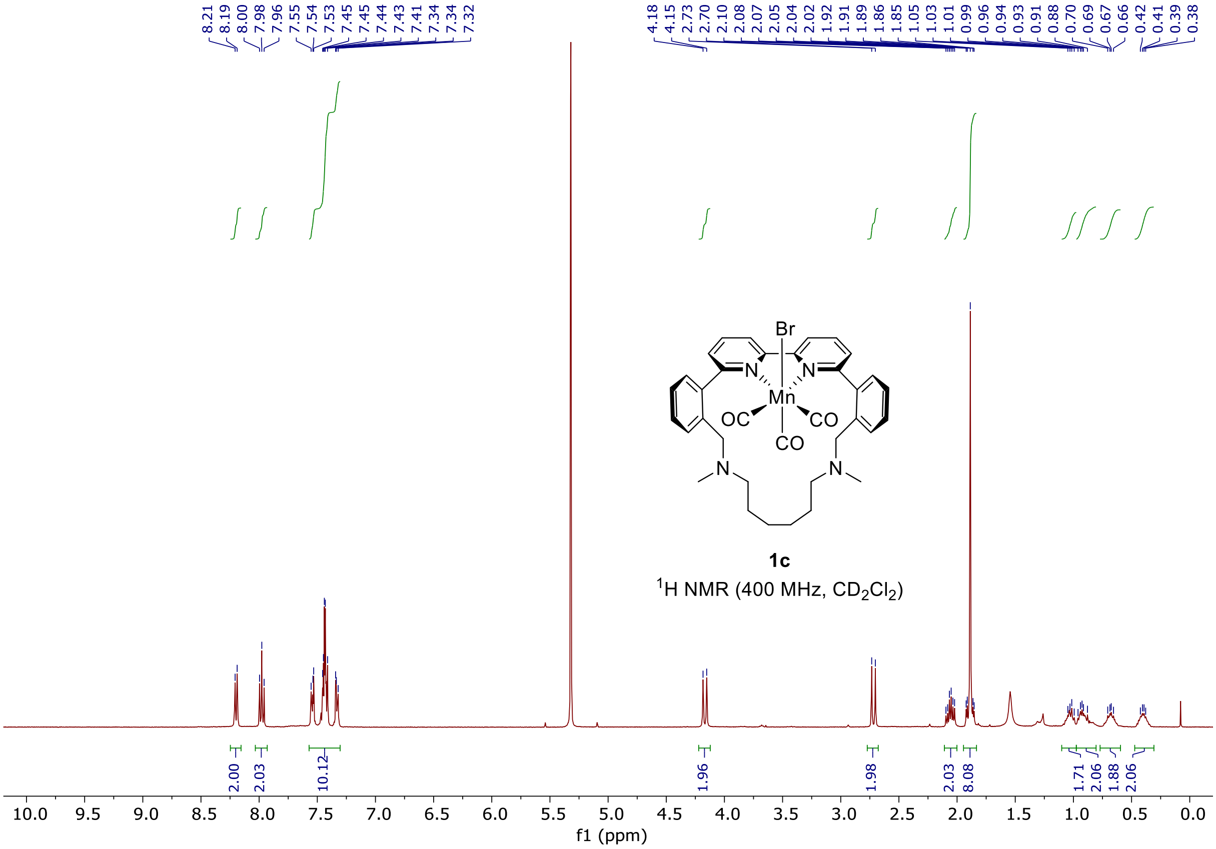
8.21 8.19 8.00 7.98 7.96 7.55 7.54 7.53 7.45 7.45 7.44 7.43 7.41 7.34 7.34 7.32
4.18 4.15 2.73 2.70 2.10 2.08 2.07 2.05 2.04 2.02 1.92 1.91 1.89 1.86 1.85 1.05 1.03 1.01 0.99 0.96 0.94 0.93 0.91 0.88 0.70 0.69 0.67 0.66 0.42 0.41 0.39 0.38
Br
N
N
Mn
OC
CO
CO
N
N
1c
$^{1}$H NMR (400 MHz, $CD_2Cl_2$)
2.00 2.03 10.12 1.96 1.98 2.03 8.08 1.71 2.06 1.88 2.06
10.0 9.5 9.0 8.5 8.0 7.5 7.0 6.5 6.0 5.5 5.0 4.5 4.0 3.5 3.0 2.5 2.0 1.5 1.0 0.5 0.0
f1 (ppm)

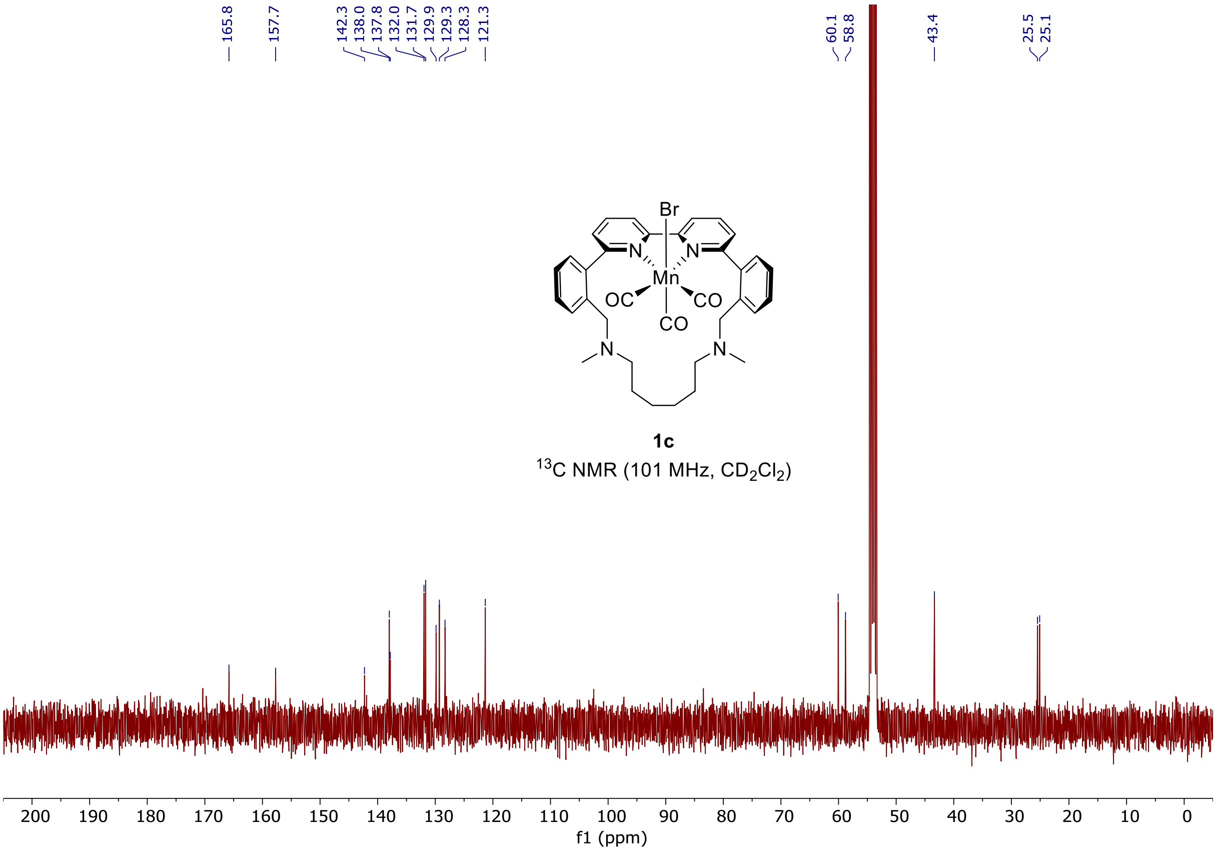
165.8 157.7 142.3 138.0 137.8 132.0 131.7 129.9 129.3 128.3 121.3 60.1 58.8 43.4 25.5 25.1
Br
N
N
Mn
OC
CO
CO
N
N
1c
$^{13}$C NMR (101 MHz, $CD_2Cl_2$)
200 190 180 170 160 150 140 130 120 110 100 90 80 70 60 50 40 30 20 10 0
f1 (ppm)

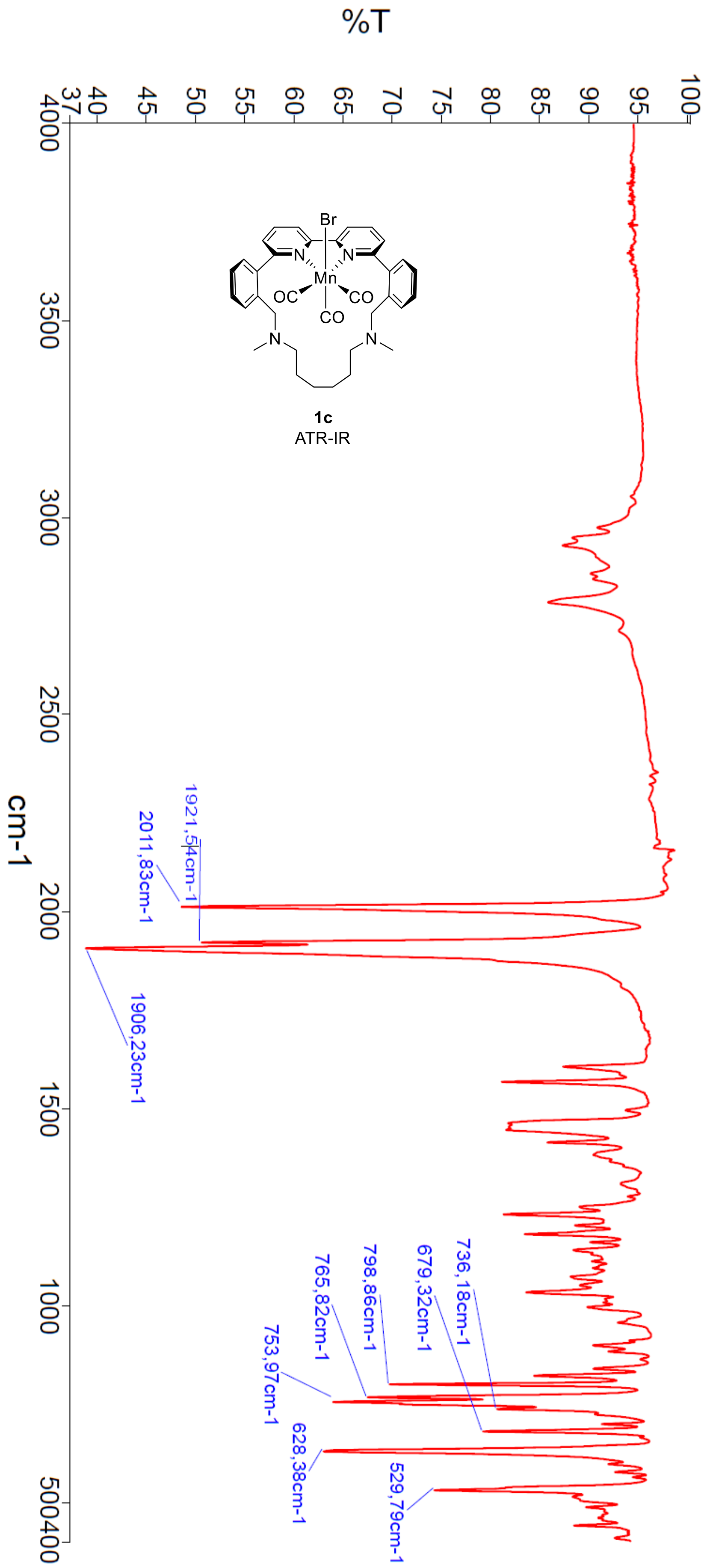
%T
cm-1
1c
ATR-IR
Br
Mn
N
OC
CO
2011,83cm-1
1921,54cm-1
1906,23cm-1
798,86cm-1
765,82cm-1
753,97cm-1
736,18cm-1
679,32cm-1
628,38cm-1
529,79cm-1

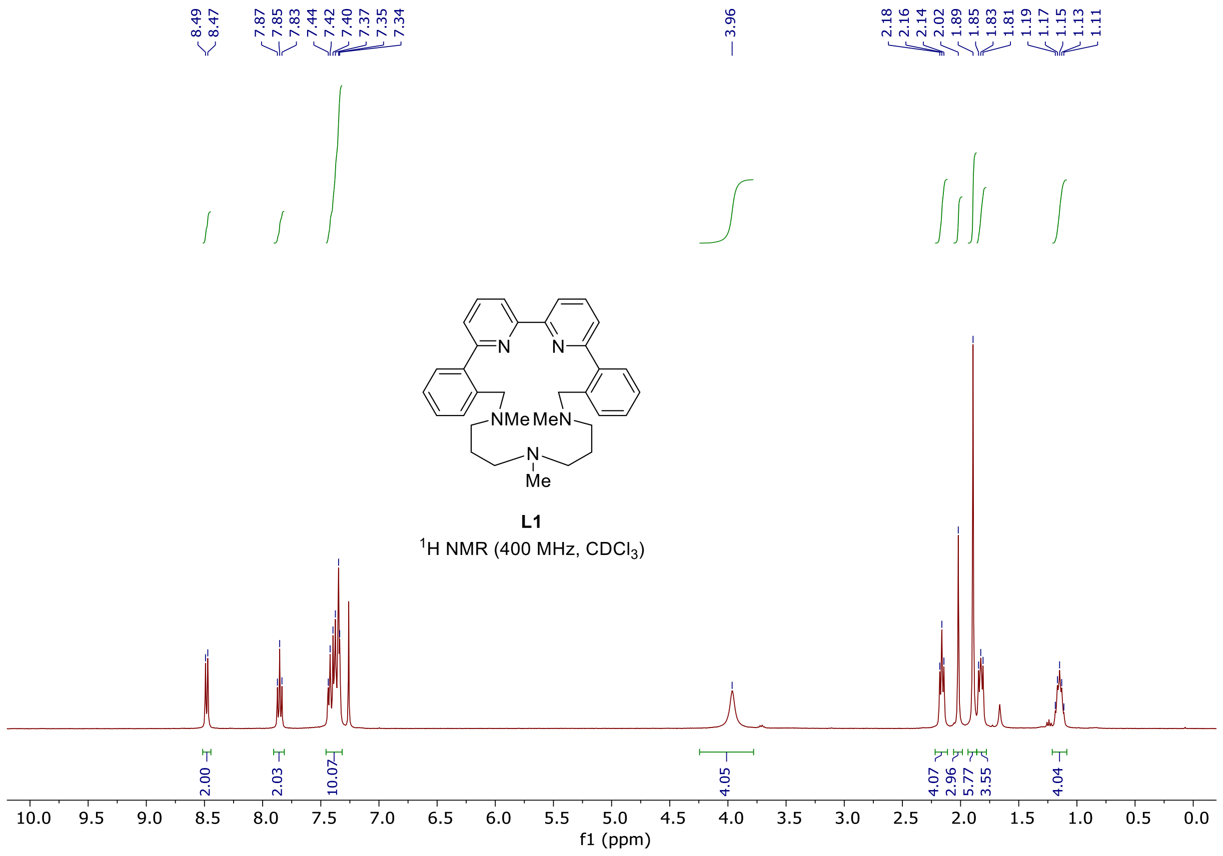
8.49
8.47
7.87
7.85
7.83
7.44
7.42
7.40
7.37
7.35
7.34
3.96
2.18
2.16
2.14
2.02
1.89
1.85
1.83
1.81
1.19
1.17
1.15
1.13
1.11
N
N
NMe MeN
N
Me
L1
$^{1}$H NMR (400 MHz, CDCl$_3$)
2.00
2.03
10.07
4.05
4.07
2.96
5.77
3.55
4.04
f1 (ppm)

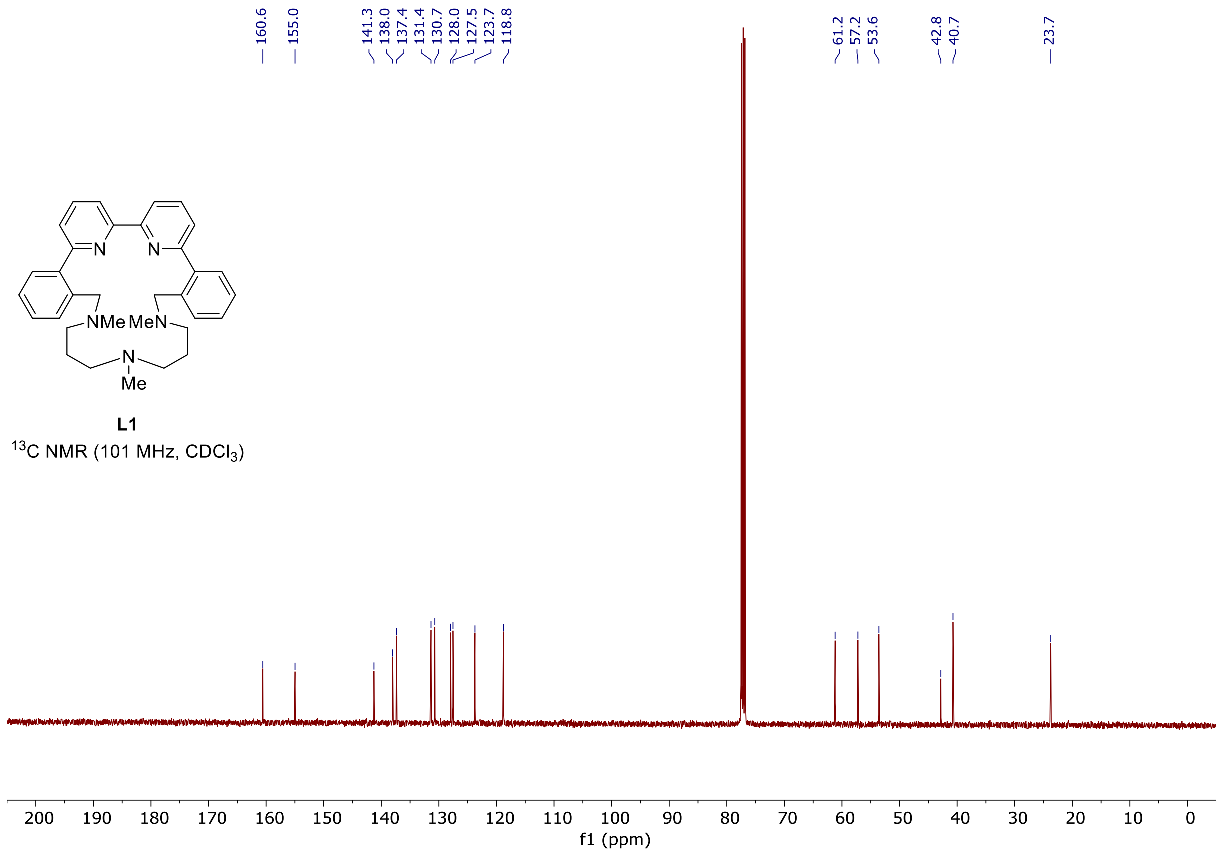
160.6
155.0
141.3
138.0
137.4
131.4
130.7
128.0
127.5
123.7
118.8
61.2
57.2
53.6
42.8
40.7
23.7
N
N
NMe MeN
N
Me
L1
$^{13}$C NMR (101 MHz, CDCl$_3$)
f1 (ppm)

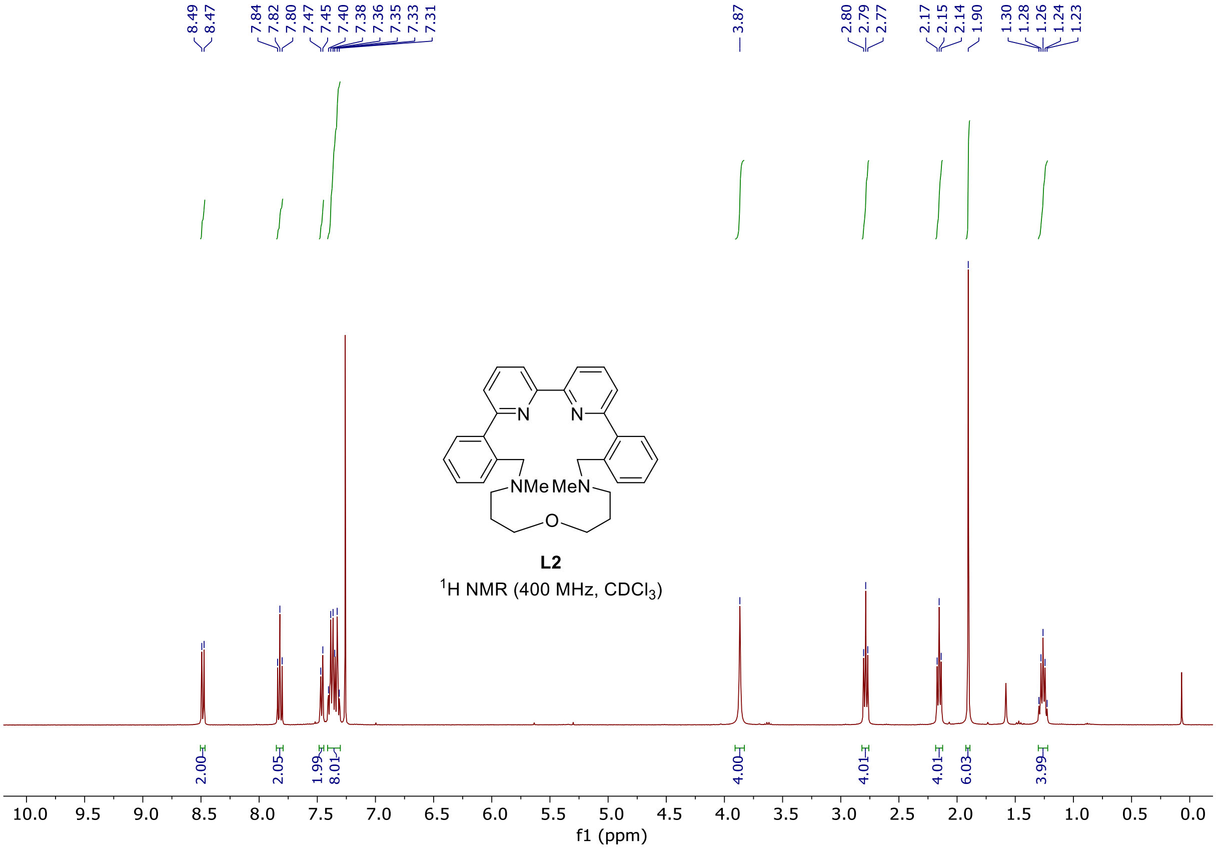
8.49
8.47
7.84
7.82
7.80
7.47
7.45
7.40
7.38
7.36
7.35
7.33
7.31
3.87
2.80
2.79
2.77
2.17
2.15
2.14
1.90
1.30
1.28
1.26
1.24
1.23
NMe MeN
N
N
O
L2
$^{1}$H NMR (400 MHz, $CDCl_3$)
2.00
2.05
1.99
8.01
4.00
4.01
4.01
6.03
3.99
f1 (ppm)

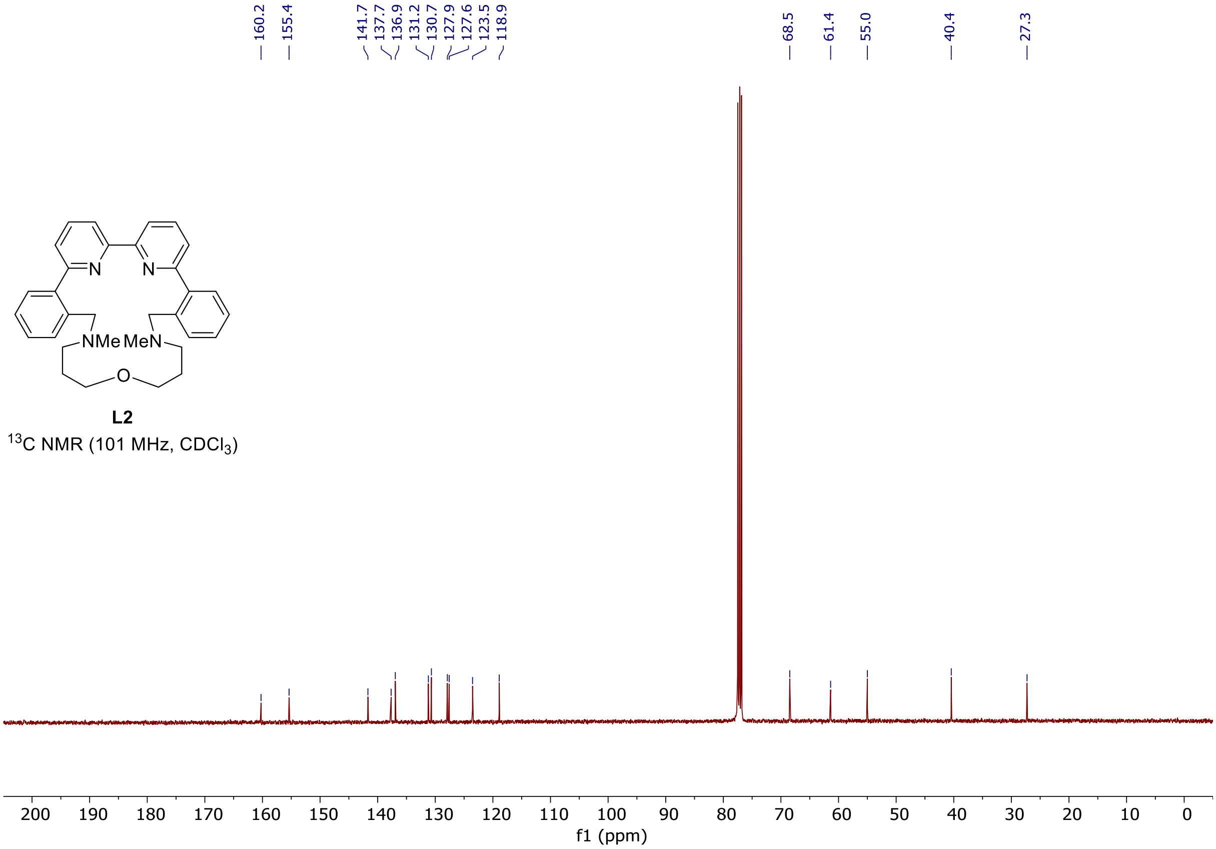
160.2
155.4
141.7
137.7
136.9
131.2
130.7
127.9
127.6
123.5
118.9
68.5
61.4
55.0
40.4
27.3
NMe MeN
N
N
O
L2
$^{13}$C NMR (101 MHz, $CDCl_3$)
f1 (ppm)

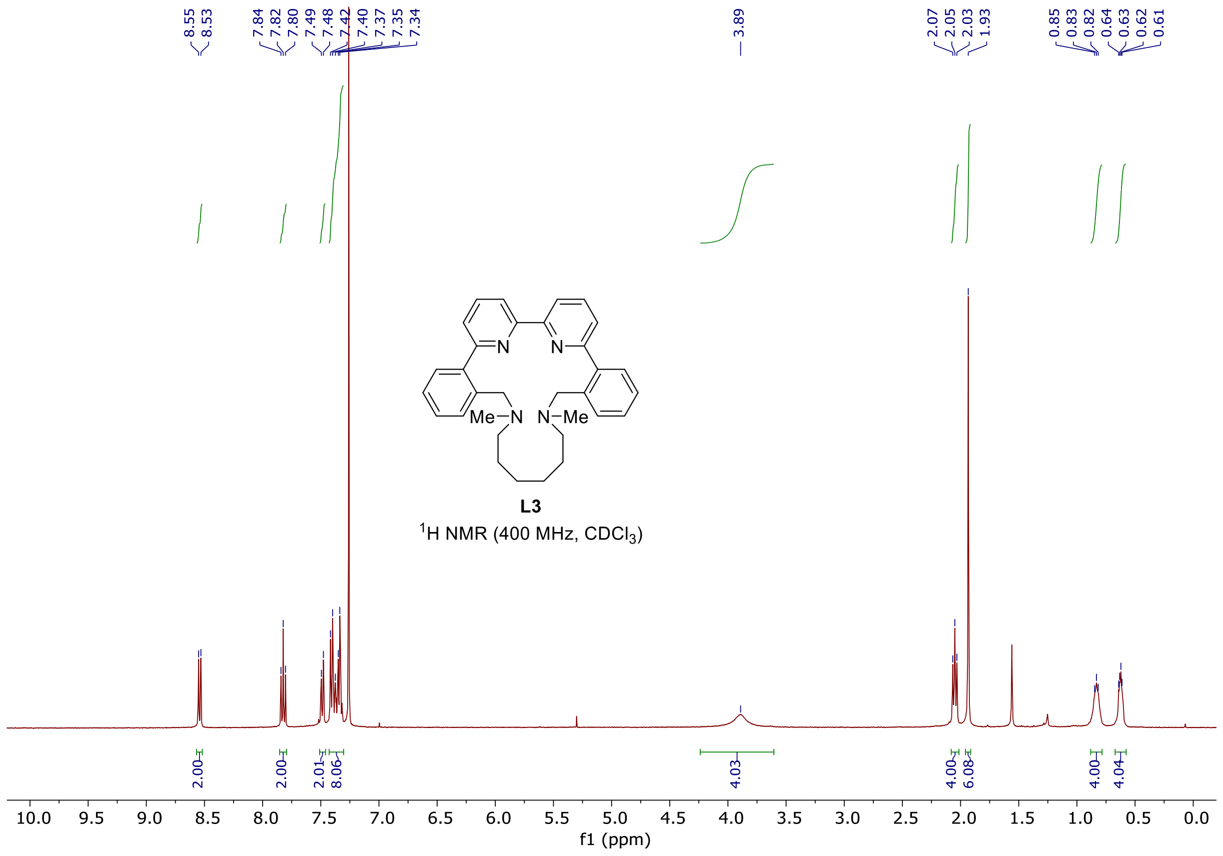
8.55
8.53
7.84
7.82
7.80
7.49
7.48
7.42
7.40
7.37
7.35
7.34
3.89
2.07
2.05
2.03
1.93
0.85
0.83
0.82
0.64
0.63
0.62
0.61
Me–N
N–Me
N
N
L3
1H NMR (400 MHz, CDCl3)
2.00
2.00
2.01
8.06
4.03
4.00
6.08
4.00
4.04
f1 (ppm)

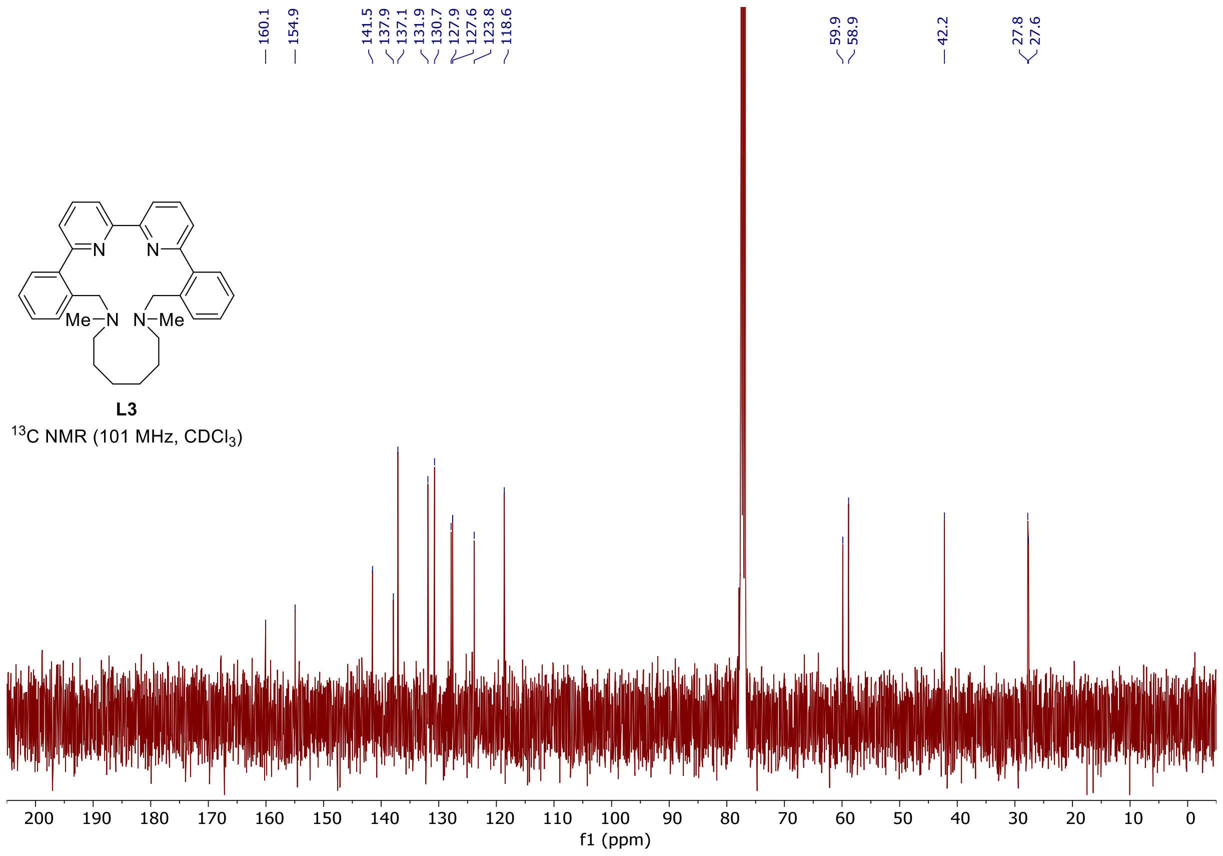
160.1
154.9
141.5
137.9
137.1
131.9
130.7
127.9
127.6
123.8
118.6
59.9
58.9
42.2
27.8
27.6
Me–N
N–Me
N
N
L3
13C NMR (101 MHz, CDCl3)
f1 (ppm)

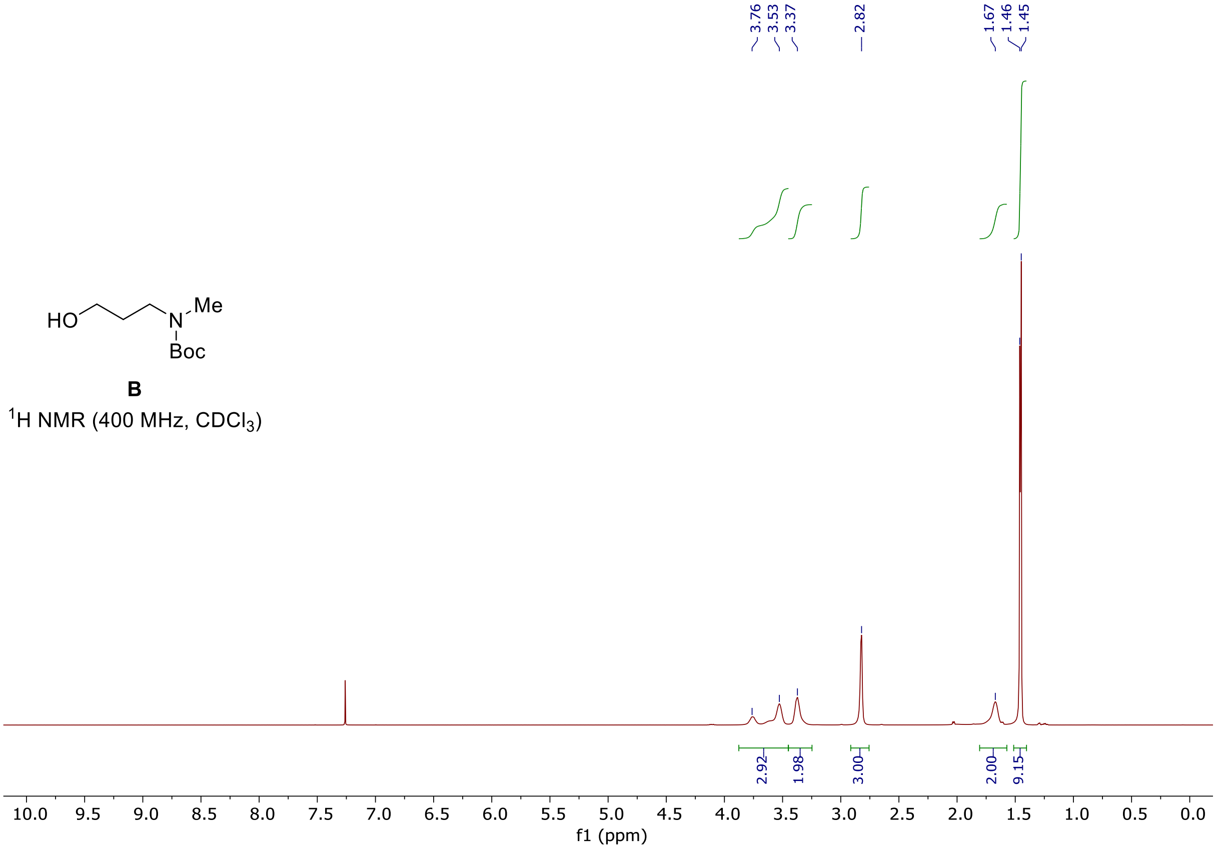


**B**

$^{1}H$ NMR (400 MHz, $CDCl_3$)

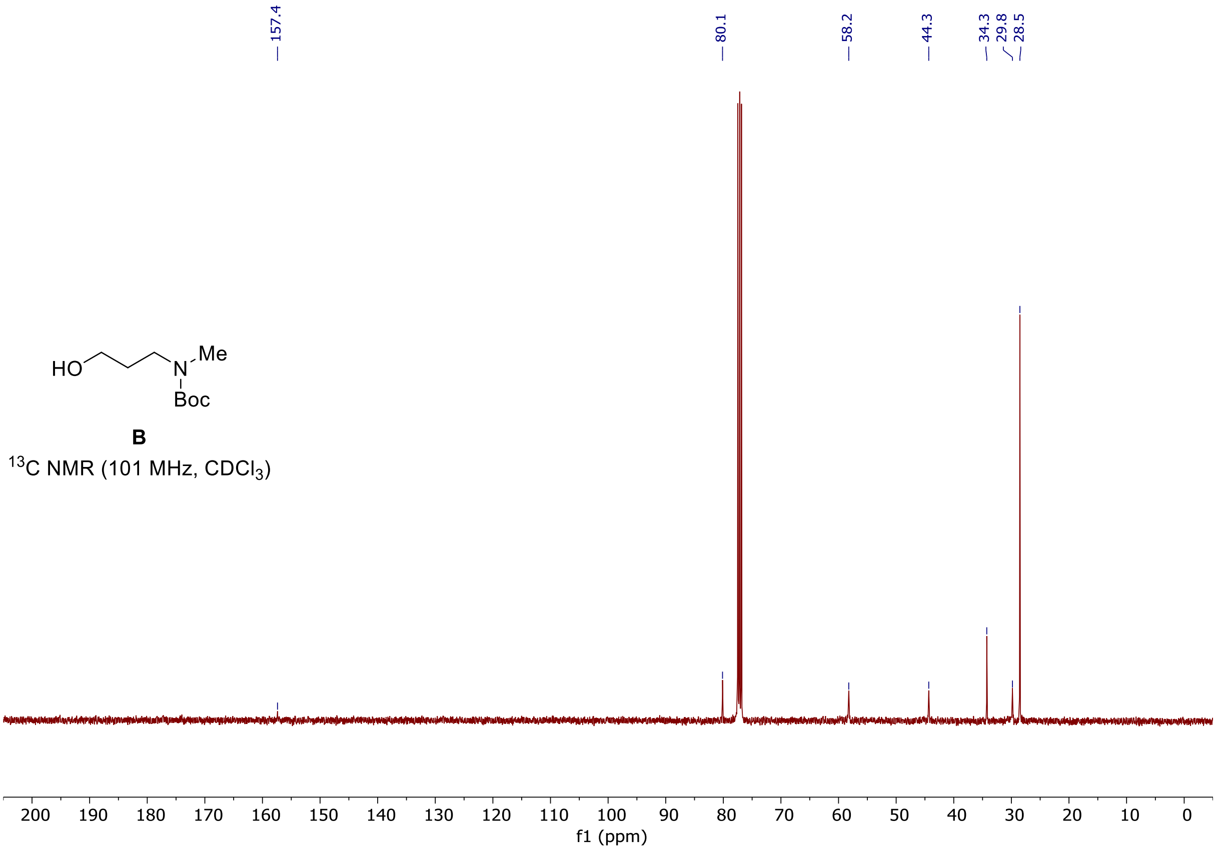


**B**

$^{13}C$ NMR (101 MHz, $CDCl_3$)

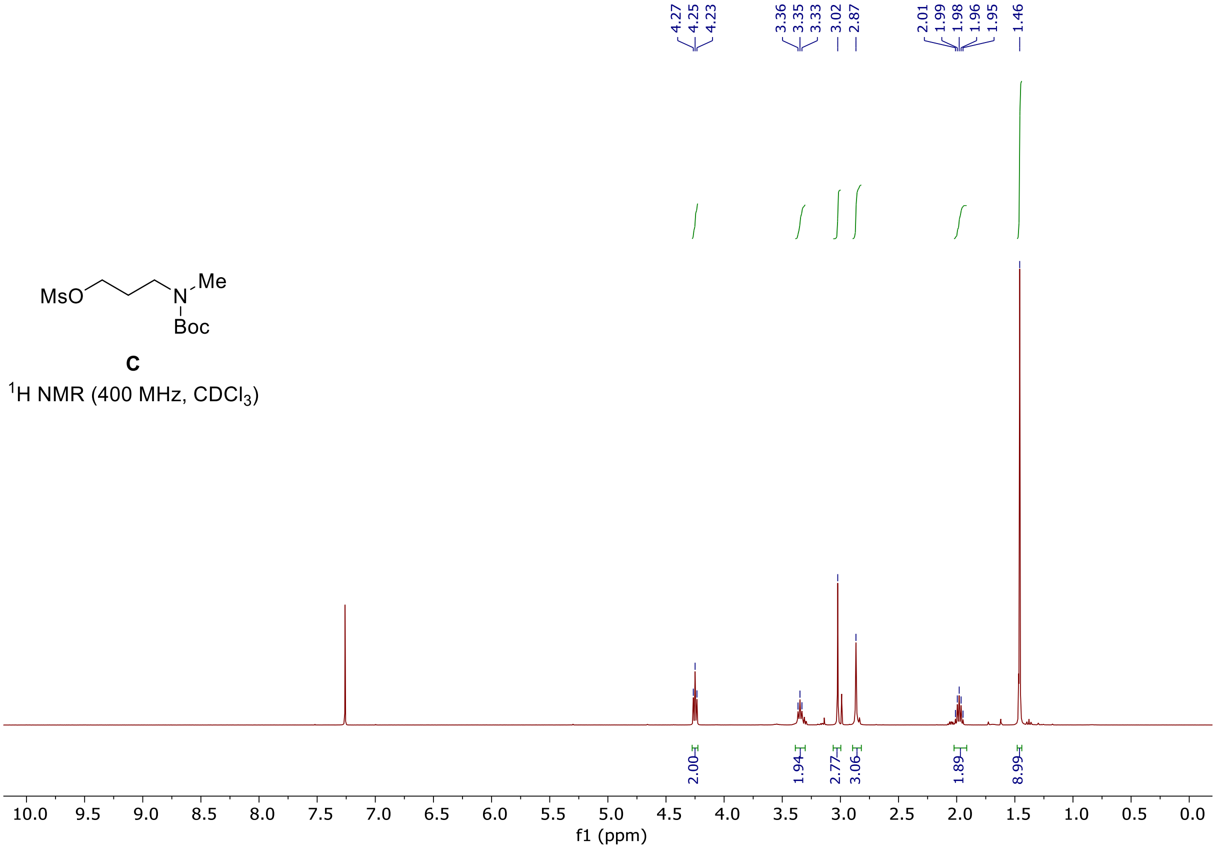

MsO
N
Me
Boc
C
1H NMR (400 MHz, CDCl3)
4.27
4.25
4.23
3.36
3.35
3.33
3.02
2.87
2.01
1.99
1.98
1.96
1.95
1.46
2.00
1.94
2.77
3.06
1.89
8.99
10.0 9.5 9.0 8.5 8.0 7.5 7.0 6.5 6.0 5.5 5.0 4.5 4.0 3.5 3.0 2.5 2.0 1.5 1.0 0.5 0.0
f1 (ppm)


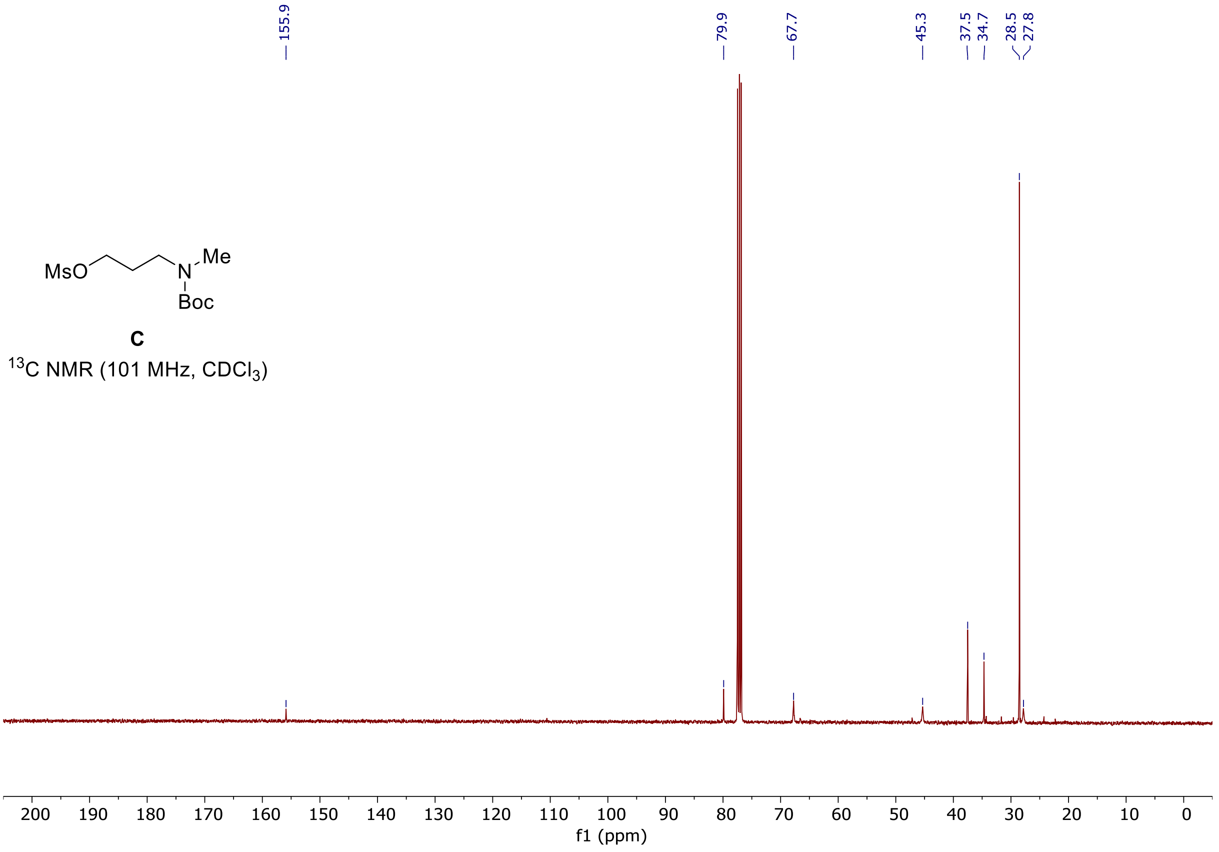

MsO
N
Me
Boc
C
13C NMR (101 MHz, CDCl3)
155.9
79.9
67.7
45.3
37.5
34.7
28.5
27.8
200 190 180 170 160 150 140 130 120 110 100 90 80 70 60 50 40 30 20 10 0
f1 (ppm)

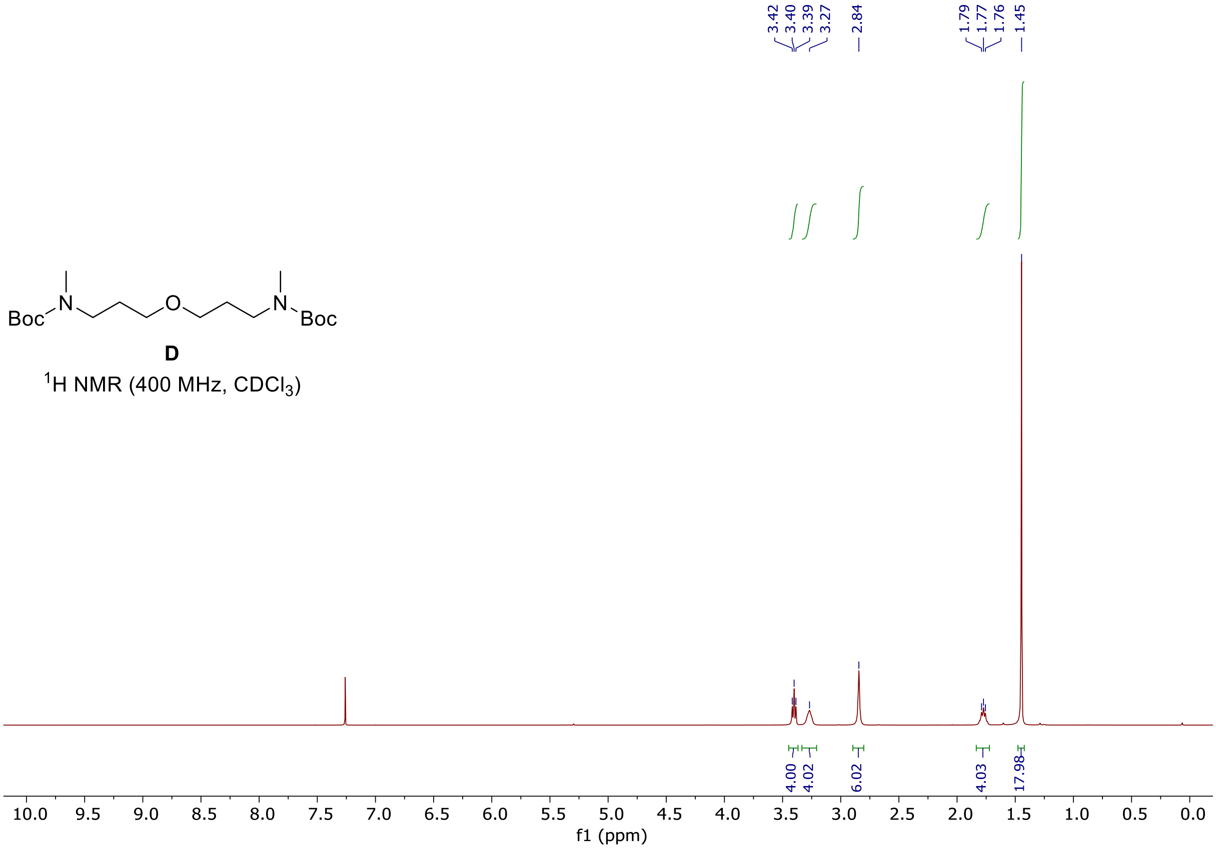
3.42
3.40
3.39
3.27
2.84
1.79
1.77
1.76
1.45
Boc
N
O
N
Boc
D
1H NMR (400 MHz, CDCl3)
4.00
4.02
6.02
4.03
17.98
10.0 9.5 9.0 8.5 8.0 7.5 7.0 6.5 6.0 5.5 5.0 4.5 4.0 3.5 3.0 2.5 2.0 1.5 1.0 0.5 0.0
f1 (ppm)

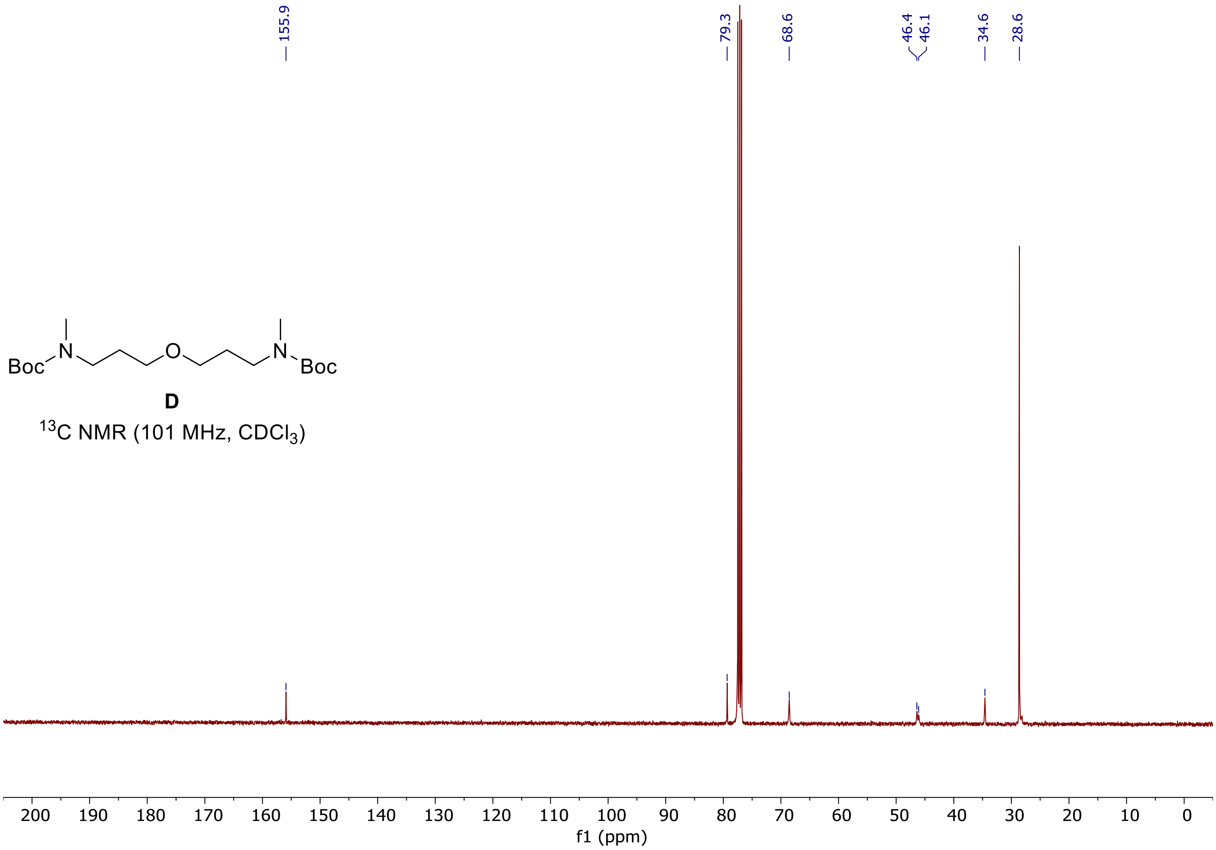
155.9
79.3
68.6
46.4
46.1
34.6
28.6
Boc
N
O
N
Boc
D
13C NMR (101 MHz, CDCl3)
200 190 180 170 160 150 140 130 120 110 100 90 80 70 60 50 40 30 20 10 0
f1 (ppm)

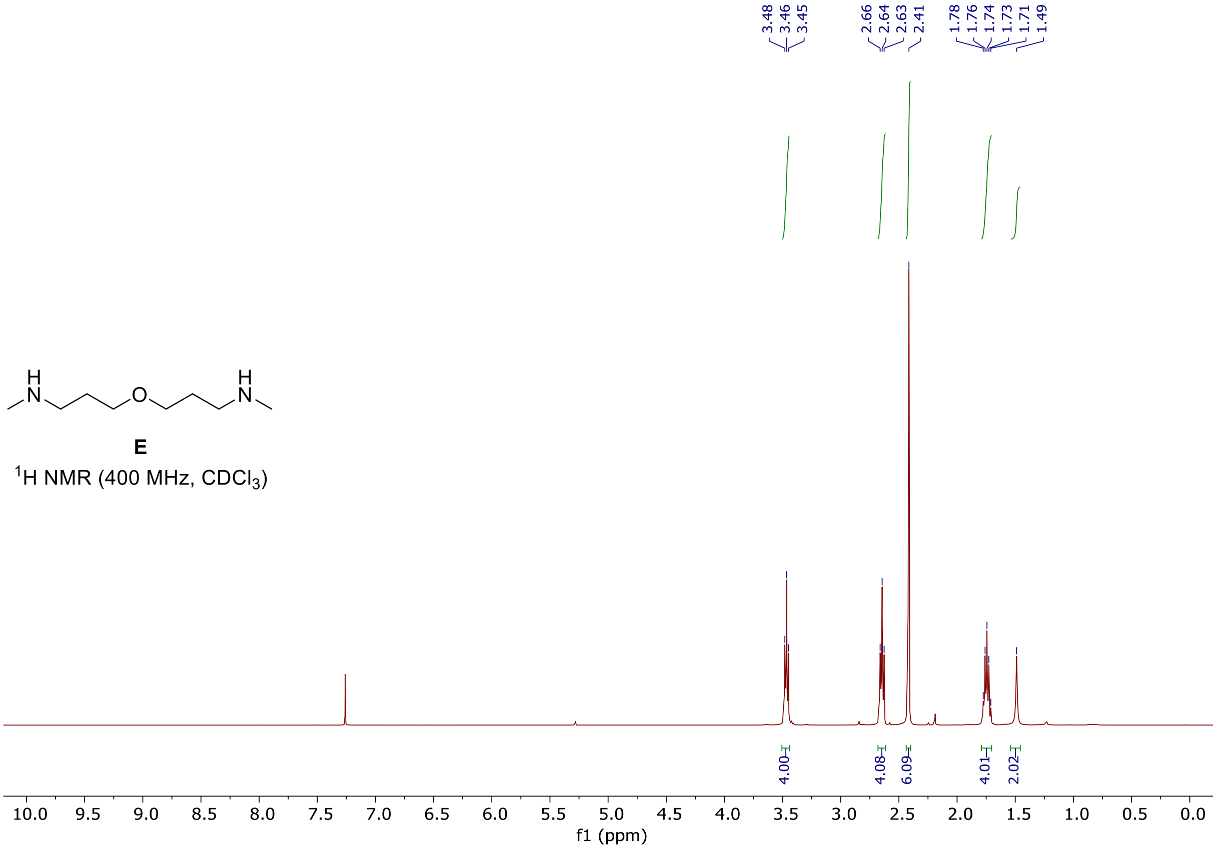
3.48
3.46
3.45
2.66
2.64
2.63
2.41
1.78
1.76
1.74
1.73
1.71
1.49
4.00
4.08
6.09
4.01
2.02
E
$^{1}$H NMR (400 MHz, $CDCl_3$)
f1 (ppm)

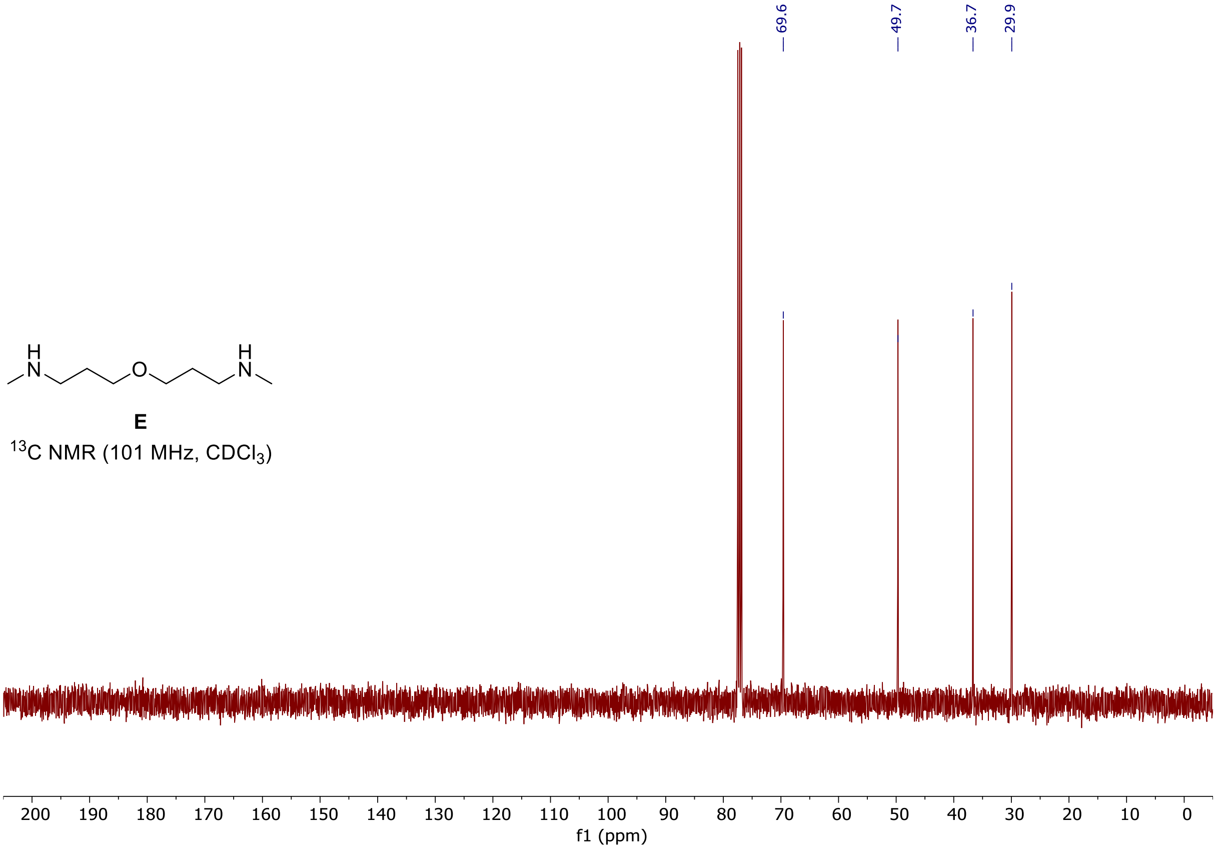
69.6
49.7
36.7
29.9
E
$^{13}$C NMR (101 MHz, $CDCl_3$)
f1 (ppm)

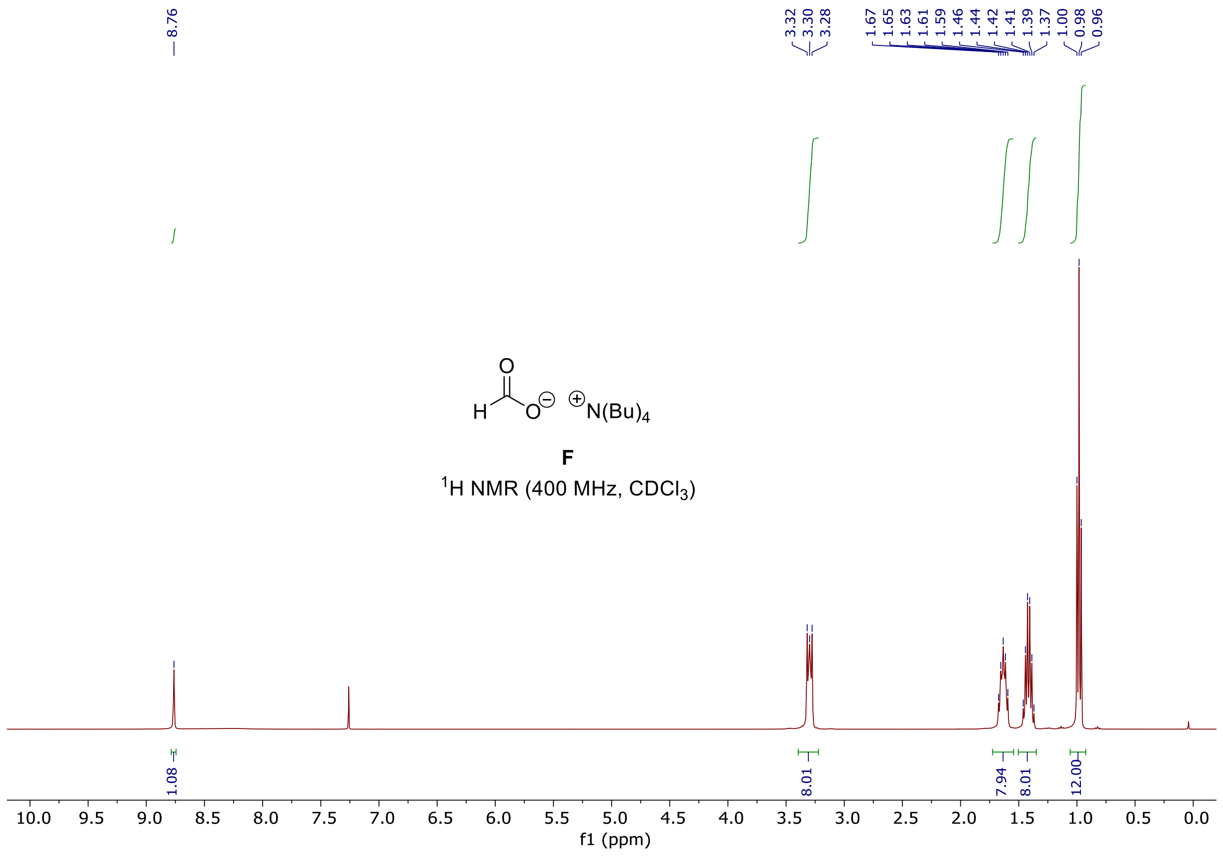
8.76
3.32
3.30
3.28
1.67
1.65
1.63
1.61
1.59
1.46
1.44
1.42
1.41
1.39
1.37
1.00
0.98
0.96
H
O
O
N(Bu)4
F
1H NMR (400 MHz, CDCl3)
1.08
8.01
7.94
8.01
12.00
10.0 9.5 9.0 8.5 8.0 7.5 7.0 6.5 6.0 5.5 5.0 4.5 4.0 3.5 3.0 2.5 2.0 1.5 1.0 0.5 0.0
f1 (ppm)

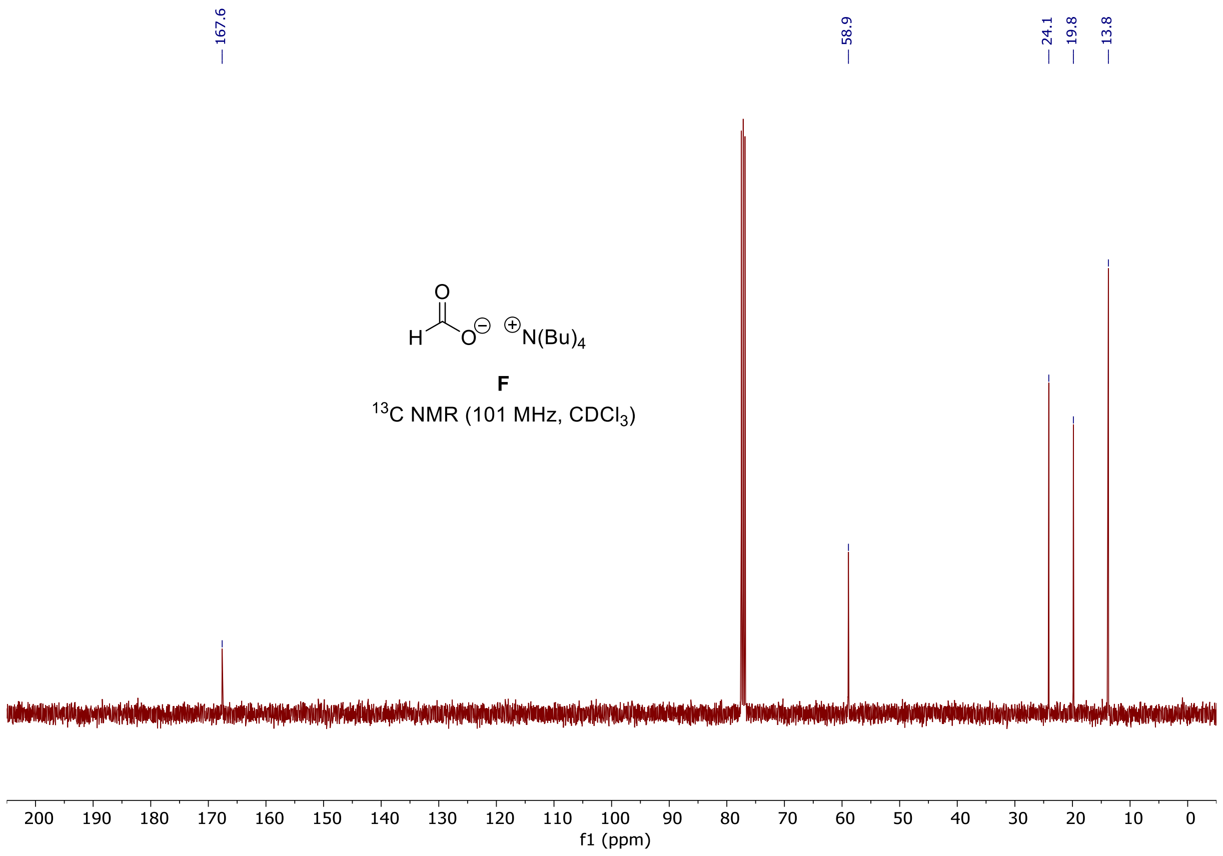
167.6
58.9
24.1
19.8
13.8
H
O
O
N(Bu)4
F
13C NMR (101 MHz, CDCl3)
200 190 180 170 160 150 140 130 120 110 100 90 80 70 60 50 40 30 20 10 0
f1 (ppm)

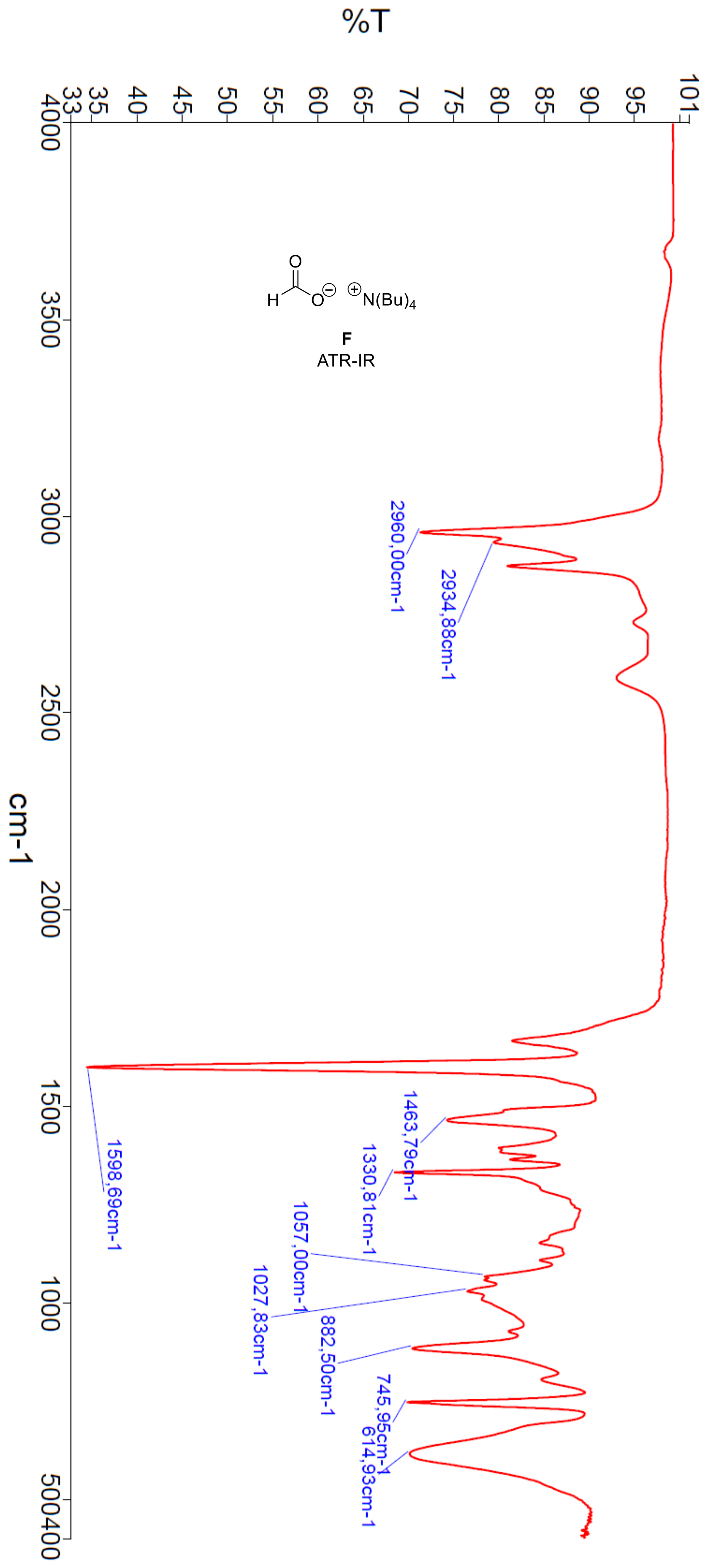
%T
cm-1
F
ATR-IR
2960,00cm-1
2934,88cm-1
1598,69cm-1
1463,79cm-1
1330,81cm-1
1057,00cm-1
1027,83cm-1
882,50cm-1
745,95cm-1
614,93cm-1
